# Genetic correlation between smoking behavior and gastroesophageal reflux disease: insights from integrative multi-omics data

**DOI:** 10.1186/s12864-024-10536-3

**Published:** 2024-06-27

**Authors:** Zhaoqi Yan, Yifeng Xu, Keke Li, Liangji Liu

**Affiliations:** 1 Jiangxi University of Traditional Chinese Medicine, Nanchang, Jiangxi China; 2 Affiliated Hospital of Jiangxi University of Chinese Medicine, Nanchang, Jiangxi China

**Keywords:** Gastroesophageal reflux disease, Smoking behavior, Genome-wide association study, Genetic correlation, Mendelian randomization

## Abstract

**Background:**

Observational studies have preliminarily revealed an association between smoking and gastroesophageal reflux disease (GERD). However, little is known about the causal relationship and shared genetic architecture between the two. This study aims to explore their common genetic correlations by leveraging genome-wide association studies (GWAS) of smoking behavior—specifically, smoking initiation (SI), never smoking (NS), ever smoking (ES), cigarettes smoked per day (CPD), age of smoking initiation(ASI) and GERD.

**Methods:**

Firstly, we conducted global cross-trait genetic correlation analysis and heritability estimation from summary statistics (HESS) to explore the genetic correlation between smoking behavior and GERD. Then, a joint cross-trait meta-analysis was performed to identify shared “pleiotropic SNPs” between smoking behavior and GERD, followed by co-localization analysis. Additionally, multi-marker analyses using annotation (MAGMA) were employed to explore the degree of enrichment of single nucleotide polymorphism (SNP) heritability in specific tissues, and summary data-based Mendelian randomization (SMR) was further utilized to investigate potential functional genes. Finally, Mendelian randomization (MR) analysis was conducted to explore the causal relationship between the smoking behavior and GERD.

**Results:**

Consistent genetic correlations were observed through global and local genetic correlation analyses, wherein SI, ES, and CPD showed significantly positive genetic correlations with GERD, while NS and ASI showed significantly negative correlations. HESS analysis also identified multiple significantly associated loci between them. Furthermore, three novel “pleiotropic SNPs” (rs4382592, rs200968, rs1510719) were identified through cross-trait meta-analysis and co-localization analysis to exist between SI, NS, ES, ASI, and GERD, mapping the genes MED27, HIST1H2BO, MAML3 as new pleiotropic genes between SI, NS, ES, ASI, and GERD. Moreover, both smoking behavior and GERD were found to be co-enriched in multiple brain tissues, with GMPPB, RNF123, and RBM6 identified as potential functional genes co-enriched in Cerebellar Hemisphere, Cerebellum, Cortex/Nucleus accumbens in SI and GERD, and SUOX identified in Caudate nucleus, Cerebellum, Cortex in NS and GERD. Lastly, consistent causal relationships were found through MR analysis, indicating that SI, ES, and CPD increase the risk of GERD, while NS and higher ASI decrease the risk.

**Conclusion:**

We identified genetic loci associated with smoking behavior and GERD, as well as brain tissue sites of shared enrichment, prioritizing three new pleiotropic genes and four new functional genes. Finally, the causal relationship between smoking behavior and GERD was demonstrated, providing insights for early prevention strategies for GERD.

**Supplementary Information:**

The online version contains supplementary material available at 10.1186/s12864-024-10536-3.

## Introduction

Gastroesophageal reflux disease (GERD) is defined as a condition that occurs when the reflux of stomach contents causes troublesome symptoms and/or complications [1]. The potential for GERD to precipitate a variety of disease conditions, such as reflux esophagitis, has been thoroughly demonstrated [2]. Additionally, GERD imposes significant direct and indirect costs on healthcare systems globally. Due to dietary habits, GERD represents one of the most common gastrointestinal issues in Western populations, with an average prevalence of 19.8% in North America and 15.2% in Europe [3].

It is well-established that smoking is a major risk factor for numerous health issues, including various diseases of the digestive system [4], among which GERD is notably affected. Epidemiological studies have shown a significant association between smoking and GERD or reflux symptoms [5–8], and cessation of smoking has been found to alleviate related symptoms [9, 10]. For instance, a large case–control study by Nilsson M [7] showed that individuals smoking more than 20 cigarettes daily had a 70% increased likelihood of reflux symptoms compared to non-smokers, and Fujiwara Y [8] found that those smoking more than one pack a day were more prone to a range of digestive diseases including GERD and functional dyspepsia. Additionally, Kohata Y [9] demonstrated that successful smoking cessation for one year significantly reduced the frequency of reflux symptoms and improved health-related quality of life (HR-QOL). However, these studies are observational in nature, with limited sample sizes and susceptible to various confounding factors (such as challenges in measuring differences in individuals' dietary habits). Importantly, they do not offer conclusive evidence for the causal relationship between smoking behavior and gastroesophageal reflux, leaving the genetic relationship between the two still unclear.

To date, few studies have investigated the potential genetic relationship between smoking behavior and GERD. With the rapid development of genomic sequencing technologies, linking traits to genetics has become an effective method to overcome the limitations of observational studies [11]. In this research, we, for the first time, examined the genetic correlation and potential causal relationship between various smoking behavior and GERD based on large-scale GWAS summary data. Specifically, we first quantified global and local genetic correlations to explore the shared genetic basis between smoking behavior and GERD. Then, we employed cross-trait meta-analysis and colocalization analysis to quantify precise genetic correlations, uncover new “pleiotropic SNPs,” and identify pleiotropic genes. Moreover, we analyzed the tissue-specific enrichment of genetic associations between smoking behavior and GERD and identified potential functional genes. Lastly, we conducted a Mendelian randomization (MR) analysis to infer their causal effects.

## Materials and methods

### Data Sources

Exposure Data—Smoking Behavior: Tobacco use data were extracted from GWAS and the Alcohol and Nicotine use Sequencing Consortium (GSCAN), including smoking initiation (SI), quantity of cigarettes smoked per day (CPD), and age of smoking initiation (ASI) [12]. An individual is only considered to have a history of smoking behavior if they have smoked more than 100 cigarettes in their lifetime. SI is a binary phenotype defined as regular smoking in daily life and continuing to smoke within the last month. This dataset includes 607,291 samples, with 311,629 cases and 321,173 controls, totaling 11,802,365 SNPs. CPD and ASI were encoded as continuous traits in GWAS, with sample sizes of 337,334 and 341,427, respectively, totaling 11,913,712 and 11,894,779 SNPs. Data for never-smoking were derived from the UK Biobank (UKB), where individuals were classified as never smoking (NS) if they reported “Current tobacco smoking” (Field 1239) as no (0) AND “Past tobacco smoking” (Field 1249) as never (4), comprising 195,068 cases and 164,638 controls. Ever smoking (ES) was derived from “Current tobacco smoking” (Field 1239) and “Past tobacco smoking” (Field 1249), considering individuals as ES if they denied current tobacco use but affirmed past frequency, including 280,508 cases and 180,558 controls. All of the above specific questionnaire scales were defined by Dr. Laura J. et al. (Supplementary Materials—Table S1) [12]. All exposure genetic IVs were selected within a 1 Mb region at a genome-wide significance level (*P* < 5E-08).

Outcome Data – GERD: Summary data for the GERD phenotype came from a multi-trait genetic association analysis conducted by Ong JS et al. [13], identifying 88 loci associated with GERD, including 129,080 cases and 473,524 controls, totaling 2,320,781 SNPs. The data used in this study were stripped of rare variants, including filtering to remove imputation information value < 0.90 and minor allele frequency < 0.01 SNPs. The analysis was referenced to the 1000 Genomes Project, excluding sex chromosomes and the human leukocyte antigen (HLA) region. All participants were of European ancestry.

### Genetic Correlation Analysis

To investigate the shared genetic basis between smoking behavior and GERD, we employed various methods for correlation analysis, including global cross-trait genetic correlation analysis and local genetic correlation analysis (Heritability Estimation from Summary Statistics, HESS), to study their genetic correlation and identify common genetic loci between them. For cross-trait linkage disequilibrium score regression (LDSC), we estimated the liability scale SNP heritability of smoking behavior and GERD using stratified LD score regression (S-LDSC) and the baseline-LD model [14]. S-LDSC allows for the verification of heritability explained by given genomic features by grouping SNPs into areas of interest based on linkage disequilibrium (LD). We then applied bivariate LD score regression [15], a method that uses the expected relationship between LD and GWAS association statistics to estimate genetic correlation between traits, considering potential sample overlap between studies. In this research, we primarily used bivariate LDSC with an unconstrained intercept to assess the genetic correlation (Rg) between smoking behavior and GERD. Despite efforts to minimize sample overlap in selecting GWAS data for the five smoking behavior and GERD, we also conducted LDSC with a constrained intercept as a sensitivity analysis, where the constrained intercept LDSC replaces χ^2^ with z-scores from two studies, then estimates genetic covariance using the regression slope of the two z-scores on LD scores, normalized by SNP heritability to produce genetic correlation [15]. It’s noteworthy that the baseline-LD model [16] used a method based on continuous rather than binary annotations to partition SNP heritability.

To precisely quantify which loci of smoking behavior contribute to the whole-genome genetic correlation with GERD, we employed the HESS method to estimate local SNP heritability for each trait and genetic covariance between traits [17]. The local genetic correlation estimates are then computed from the local single-tit SNP heritability and the local cross-tit genetic covariance estimates. The algorithm divides the whole genome into 1703 loci based on LD patterns in the European population, with an average size close to 1.5 Mb, and quantifies the trait correlations caused by genetic variation confined to a specific locus. Strict Bonferroni correction was applied for adjustment (i.e., ρ-HESS < 0.05/ (NUM_SNP_) where NUM_SNP_ is the number of SNPs per loci region).

### Cross-trait Meta-analysis

To identify new “pleiotropic SNPs” associated with the combined phenotype (smoking behavior and GERD), we also conducted two cross-trait meta-analyses, including Multi-trait analysis of GWAS (MTAG) [18] and Cross Phenotype Association (CPASSOC) [19]. MTAG is a generalized meta-analysis method that enhances the statistical power to estimate genotype–phenotype variance–covariance matrices, thereby generating trait-specific estimates for each SNP [20]. MTAG adjusts for possible errors brought by sample overlap using bivariate LD score regression. MTAG is appropriate when all variants have the same effect size across traits and generates trait-specific association statistics. We calculated the upper limit of the false discovery rate ('maxFDR') to test the equal variance–covariance assumption. Additionally, CPASSOC integrates association evidence from multiple traits to detect variants affecting at least one trait. CPASSOC assumes cross-trait heterogeneity of effects and estimates cross-trait statistical heterogeneity (SHet) and *p*-values through sample size-weighted meta-analysis of GWAS summary data. We prioritized independent SNPs with genome-wide significance in both MTAG and CPASSOC (*P* < 5E-08), and those SNPs not previously reported in GWAS for any of the six traits related to GERD or smoking behavior were considered as “novel SNPs” for the association between smoking behavior and GERD [21]. Lastly, these “novel SNPs” located within loci identified by HESS were termed “pleiotropic SNPs.”

Colocalization analysis was then used to verify whether these “pleiotropic SNPs” share common genetic variants between smoking behavior and GERD. We used the colco.abf function in the R package “Coloc.” “Coloc” uses a Bayesian algorithm to generate posterior probabilities for five mutually exclusive hypotheses, with posterior probabilities PPH_3_ + PPH_4_ > 0.8 typically interpreted as colocalization [22]. The combined significance of cross-trait meta-analysis, HESS, and colocalization analysis ensured the accuracy of the genetic influence of “pleiotropic SNPs” on the exposure-outcome relationship. We also employed Manhattan plots to visualize significant mapped genes on loci after HESS analysis (*P* < 5E-08) as well as genes mapped by these “pleiotropic SNPs.”

### Tissue-specific Enrichment of SNP Heritability

To establish the most relevant tissues between smoking behavior and GERD, we conducted SNP heritability enrichment analysis for different tissues using MAGMA (Multi-marker Analysis of GenoMic Annotation) with genotype-tissue expression (GTEx) data. GTEx (v.8) provides up to 53 tissue types [23], evaluating the association between genes specifically expressed in each tissue and smoking behavior and GERD through cell type-specific analysis, with Bonferroni correction applied for multiple testing to strengthen the reliability of these associations (*P* < 0.05/53 = 9.43E-04). Additionally, the Summary-data-based Mendelian Randomization (SMR) method integrates summary statistics from GWAS and Expression Quantitative Trait Loci (eQTL) consortium studies to test the correlation between gene expression and target phenotypes [24]. Thus, in tissues with shared heritability enrichment between smoking behavior and GERD, the SMR method allows us to further identify assumed functional genes with a statistical association between smoking behavior and GERD using genome-wide significant SNPs, while the heterogeneity in dependent instruments (HEIDI) test assesses linkage to distinguish causality or pleiotropy from linkage [24]. Significant shared functional genes between smoking behavior and GERD are defined as passing both the Benjamini–Hochberg FDR test and the HEIDI outlier test (*P* > 0.05, N > 10 SNPs) in SMR analysis for both traits.

### Mendelian Randomization Analysis

To test evidence of a potential causal relationship between smoking behavior and GERD, we conducted MR analysis using genetic instrumental variables (IV). Due to the potential presence of sample overlap, we evaluated the potential type 1 error that may arise in this situation (https://sb452.shinyapps.io/overlap/) and corrected the lower limit of the F-statistic of the exposure IV to mitigate bias from weak IVs. Furthermore, we validated the MR result using GERD from the latest release of the FinnGen database R10 data (sample = 378,923, cases = 28,859, controls = 350,064) as the outcome variable (https://storage.googleapis.com/finngen-public-data-r10/summary_stats/finngen_R10_K11_REFLUX.gz). These methods were implemented to reduce errors due to sample overlap, ensuring the reliability of the MR results. The inverse variance weighted (IVW) method served as the primary analysis method, summarizing estimates from each genetic variant (IV) and calculating a precise causal estimate, assuming all genetic variants are valid, or balancing the overall pleiotropy to zero. MR-Egger regression, weighted median (WM), weighted mode, and generalized summary-data-based Mendelian randomization (GSMR) analyses were used as complementary methods to enhance the reliability of causal inference [25]. Additionally, MR-PRESSO and Leave-one-out sensitivity analyses were conducted to test for SNPs with an outsized impact on MR estimates; Cochran's Q statistic *P*-value was used to assess heterogeneity of results; the presence of pleiotropy was determined by the intercept term of the MR-Egger method. By utilizing data from the GSCAN Consortium for exposure selection and considering the potential association of alcohol with GERD, we implemented a Multivariable Mendelian Randomization (MVMR) model to account for this factor in causal inference. The weekly alcohol consumption (DPW) also comes from the study by Liu M in GSCAN, and is defined as the average alcohol consumption reported by participants per week. For the weekly alcohol consumption, we take the midpoint of the reported range. For example, if a person reports drinking 1–5 glasses of alcohol per week, we assume they drink an average of 2.5 glasses per week. MVMR extends the standard univariate MR approach to assess the causal effects of various exposures on outcomes and estimate the direct causal effects of each exposure in a unified analysis [26].

## Results

### Genetic Correlation

We first employed S-LDSC and baseline LD modeling to estimate the SNP heritability of smoking behavior and GERD. The liability scale SNP heritability for SI was 5.64% (95% CI = 5.29 ~ 5.99%), NS was 10.35% (95% CI = 9.78 ~ 10.92%), ES was 8.12% (95% CI = 7.69 ~ 8.55%), CPD was 6.32% (95% CI = 4.91 ~ 7.69%), ASI was 4.29% (95% CI = 3.88 ~ 4.70%), and for GERD was 56.95% (95% CI = 53.28 ~ 60.62%). Then, we utilized bivariate LDSC to estimate the genetic correlation between smoking behavior and GERD. SI (rg = 0.69, *P* = 2.93E-199), ES (rg = 0.4, P = 9.76E-139), and CPD (rg = 0.66, *P* = 2.07E-60) showed significant positive genetic correlations with GERD, while NS (rg = -0.65, *P* = 5.22E-199) and ASI (rg = -0.86, *P* = 2.16E-293) exhibited significant negative correlations. Additionally, with the intercept close to 1 and genetic covariance close to 0.01 between smoking behavior and GERD traits, the possibility impact of overlapping samples on the results appears to be relatively minor. To further validate the results and considering the possibility of slight overlap between samples, we constrained the intercept in LDSC analysis without assuming population stratification. The liability scale SNP heritability for smoking initiation was 6.97% (95% CI = 6.51 ~ 7.42%), never smoking was 9.82% (95% CI = 9.06 ~ 10.58%), ever smoking was 7.66% (95% CI = 7.07 ~ 8.25%), cigarettes smoked per day was 7.51% (95% CI = 5.48 ~ 9.52%), smoking age was 4.87% (95% CI = 4.26 ~ 5.48%), and for GERD was 80.51% (95% CI = 76.01 ~ 84.99%). SI (rg = 0.43, *P* = 1.91E-140), ES (rg = 0.25, *P* = 4.59E-37), and CPD (rg = 0.42, *P* = 8E-34) exhibited significant positive genetic correlations with GERD, while NS (rg = -0.41, *P* = 1.22E-155) and ASI (rg = -0.55, *P* = 1.69E-113) showed significant negative correlations. These estimates remained relatively stable, indicating unlikely sample overlap among the five smoking behavior and GERD populations.

**Fig. 1:**
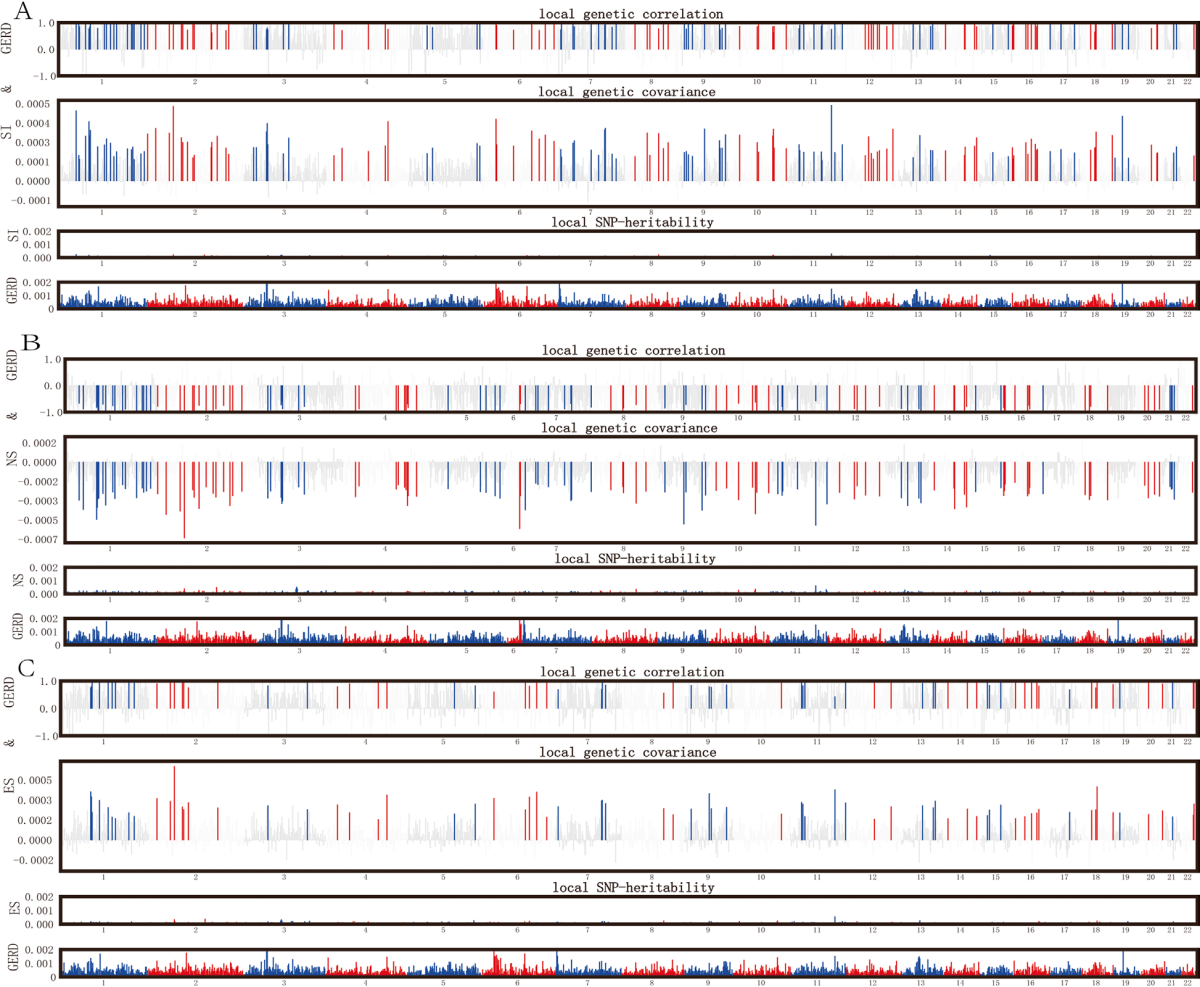
Local genetic correlation study of SI, NS, ES with GERD. The Manhattan plot illustrates estimates of local genetic correlation and local genetic covariance between smoking behavior (SI, NS, ES) and GERD, along with local SNP heritability for each smoking behavior. In the 'local genetic correlation' and 'local genetic covariance,' red and blue bars indicate significant regions of shared SNP heritability after multiple adjustments (local SNP heritability test *P* < 5E-08, local genetic covariance test *P* < 0.05/ NUM_SNP_). SI: Smoking initiation; NS: Never Smoking; ES: Ever Smoking; GERD: Gastroesophageal reflux disease; HESS: Heritability estimation from summary statistics

**Fig. 2:**
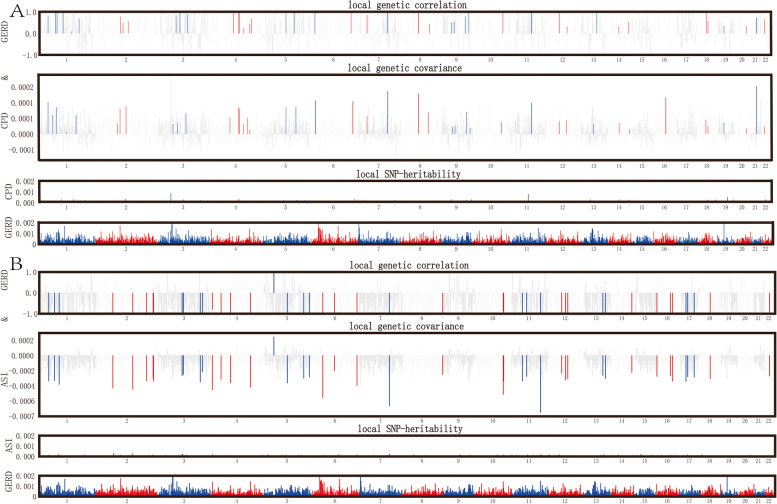
Local genetic correlation study of CPD, ASI with GERD. The Manhattan plot illustrates estimates of local genetic correlation and local genetic covariance between smoking behavior (CPD, ASI) and GERD, along with local SNP heritability for each smoking behavior. In the ‘local genetic correlation’ and ‘local genetic covariance,’ red and blue bars indicate significant regions of shared SNP heritability after multiple adjustments (local SNP heritability test *P* < 5E-08, local genetic covariance test *P* < 0.05/ NUM_SNP_). CPD: Cigarettes smoked per day; ASI: Age of smoking initiation; GERD: Gastroesophageal reflux disease; HESS: Heritability estimation from summary statistics

The HESS method was employed to determine the genome-wide local genetic correlations between smoking and GERD. After multiple corrections, strong local correlations were discovered between SI, NS, ES, CPD, ASI, and GERD in 142, 122, 72, 57, and 44 different loci, respectively (*P* < 0.05/(NUM_SNP_) within SNP quantity in the loci region) (Figs. 1 and 2, Supplementary Material—Table S2-6). Notably, the loci chr2(p16.1) (chr2:58,297,315–60,292,000) on chromosome 2 exhibited the most prominent association with SI, NS, ES, and GERD (P_SI = 6.04E-22, P_NS = 6.75E-23, P_ES = 1.74E-22), while the locus chr18(q12.3) (chr18: 39,892,648–42,922,106) on chromosome 18 showed the strongest association with CPD and GERD (*P* = 1.27E-15), and the locus chr11(q23.1- q23.2) (chr11: 112,459,488–114,257,728) on chromosome 11 was most strongly associated with smoking age and GERD (*P* = 8.70E-16). The heritability percentages (95% CI) for SI, NS, ES, CPD and ASI were 5.44% (5.08 ~ 5.8%), 11.8% (11.2 ~ 12.4%), 9.21% (8.72 ~ 9.7%), 7.86% (7.01 ~ 8.72%), and 4.89% (4.02 ~ 5.76%), respectively, while GERD heritability was 74.2% (72.97.3 ~ 75.43%). The genome-wide local genetic correlations (rgSI = 0.59, rgNS = -0.49, rgES = 0.32, rgCPD = 0.52, rgASI = -0.7) calculated through ρ-HESS were largely consistent with bivariate LDSC, demonstrating the strong genetic associations between these smoking behavior and GERD (Table 1).

**Table 1:** Heritability and genetic correlation between five smoking behavior and GERD

Method	Exposure	Exposure -Heritability h2(%), 95%CI	Outcome	Outcome—Heritability h^2^(%), 95%CI	Rg (Exposure—Outcome)	Intercept (Se)
LDSC-no intercept	SI	5.64% (5.29 ~ 5.99)	GERD	56.95% (53.28 ~ 60.62)	0.69	0.88 (1.06E-02)
	NS	10.35% (9.78 ~ 10.92)			-0.65	1.03 (1.13 E -02)
	ES	8.12% (7.69 ~ 8.55)			0.4	1.03 (1.16 E -02)
	CPD	6.32% (4.91 ~ 7.69)			0.66	0.95 (1.67E-02)
	ASI	4.29% (3.88 ~ 4.70)			-0.86	0.97 (8.40 E-03)
LDSC- intercept	SI	6.97% (6.51 ~ 7.42)		80.51%(76.01 ~ 84.99)	0.43	1
	NS	9.82% (9.06 ~ 10.58)			-0.41	
	ES	7.66% (7.07 ~ 8.25)			0.25	
	CPD	7.51% (5.48 ~ 9.52)			0.42	
	ASI	4.87% (4.26 ~ 5.48)			-0.55	
HESS	SI	5.44% (5.08 -5.8)		74.2% (72.97.3–75.43)	0.59	NA
	NS	11.8% (11.2–12.4)			-0.49	
	ES	9.21% (8.72 ~ 9.7)			0.32	
	CPD	7.86% (7.01 ~ 8.72)			0.52	
	ASI	4.89% (4.02–5.76)			-0.7	

*SI* Smoking initiation, *NS* Never Smoking, *ES* Ever Smoking, *CPD* Cigarettes smoked per day, *ASI* Age of smoking initiation, *GERD* Gastroesophageal reflux disease, *HESS* Heritability estimation from summary statistics, *Rg* Genetic correlation

### Identification of SNPs from cross-trait GWAS meta-analysis

**Fig. 3:**
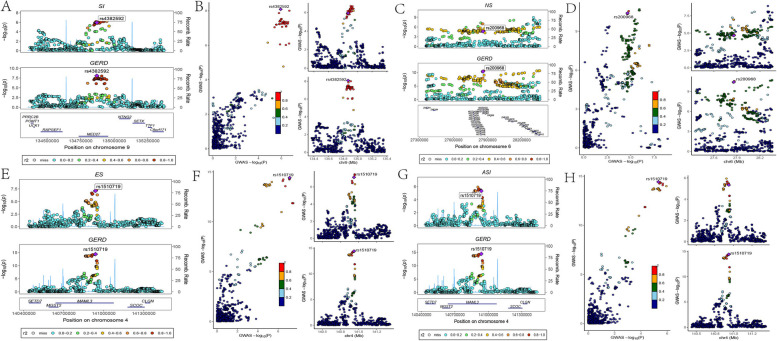
Diagram Illustrating the Co-localization Analysis of pleiotropic SNPs. For the “pleiotropic SNP” between SI – GERD, (**A**) gassocplot and (**B**) locuscomparer illustrate the number of SNPs and gene names within a 1 MB range of the mapped gene MED27 for rs4382592. For the “pleiotropic SNP” between NS – GERD, (**C**) gassocplot and (**D**) locuscomparer depict the number of SNPs and gene names within a 1 MB range of the mapped gene HIST1H2BO for rs200968. For the “pleiotropic SNP” between ES – GERD, (**E**) gassocplot and (**F**) locuscomparer show the number of SNPs and gene names within a 1 MB range of the mapped gene MAML3 for rs1510719. For the “pleiotropic SNP” between ASI –GERD, (**G**) gassocplot and (**H**) locuscomparer display the number of SNPs and gene names within a 1 MB range of the mapped gene MAML3 for rs1510719. SI: Smoking initiation; NS: Never Smoking; ES: Ever Smoking; ASI: Age of smoking initiation; GERD: Gastroesophageal reflux disease

Further cross-trait meta-analysis was conducted on the gene loci showing strong correlations between SI, NS, ES, CPD, ASI, and GERD to identify "pleiotropic SNPs" underlying the joint phenotype of smoking-related behavior and GERD. Two complementary methods, MTAG and CPASSOC, were employed, identifying 51, 35, 18, 35, and 11 "novel SNPs" that were significantly and independently associated with SI, NS, ES, CPD, ASI, and GERD at the genome-wide level, respectively (Supplementary Material—Table S7). After examination through ρ-HESS, it was found that only SI, NS, ES, and ASI had 3, 3, 2, and 4 shared "novel SNPs" with GERD, respectively, reaching genome-wide significance (SNPs marked with "*" in Table S7). Following the exclusion of SNPs significant in single-trait GWAS for SI, NS, ES, ASI, GERD, or in LD (LD r^2^ ≥ 0.02) with any previously reported significant SNPs, rs4382592 and rs9671376 emerged as novel "pleiotropic SNPs" associated with the joint phenotype of SI-GERD, mapping to the genes MED27 and TRAF3, respectively. Similarly, rs200968 emerged as a novel "pleiotropic SNP" associated with the joint phenotype of NS-GERD, mapping to the HIST1H2BO, while rs1510719 emerged as a novel "pleiotropic SNP" associated with the joint phenotype of ES-GERD, mapping to the MAML3. Additionally, rs1510719, rs2734839, rs10262103, and rs2396766 emerged as novel "pleiotropic SNPs" associated with the joint phenotype of ASI-GERD, mapping to the genes MAML3, DRD2, and FOXP2. Further co-localization analysis confirmed that rs4382592, rs200968, and rs1510719 all had shared loci between SI, NS, ES/ASI, and GERD (PPH_3_ + PPH_4_ > 0.8) (Table 2, Fig. 3), and the genes (MED27, HIST1H2BO, MAML3) mapped by these SNPs were labeled on the Manhattan plot (Supplementary Material—Figure S1-6).

**Table 2:** Co-localization analysis of pleiotropic SNPs

Exposure-outcome	SNP	N-snps	GENE	H_0_-pval	H_1_-pval	H_2_-pval	H_3_-pval	H_4_-pval
SI-GERD	rs4382592*	781	MED27	6.07E-06	1.07E-04	1.94E-03	0.03	0.96
SI-GERD	rs9671376	562	TRAF3	4.76E-05	3.34E-06	0.81	0.06	0.14
NS-GERD	rs200968*	815	HIST1H2BO	3.26E-08	5.83E-06	4.16E-03	0.74	0.25
ES-GERD	rs1510719*	807	MAML3	3.05E-12	3.88E-11	1.79E-03	0.02	0.98
ASI-GERD	rs1510719*	768	MAML3	1.96E-11	3.62E-11	1.06E-02	0.02	0.97
ASI-GERD	rs2734839	1014	DRD2	1.24E-03	5.18E-05	0.80	0.03	0.17
ASI-GERD	rs10262103	553	FOXP2	3.86E-06	4.92E-07	0.68	0.09	0.24
ASI-GERD	rs2396766	586	FOXP2	3.86E-06	4.93E-07	0.68	0.09	0.24

*SI* Smoking initiation, *NS* Never Smoking, *ES* Ever Smoking, *ASI* Age of smoking initiation, *GERD* Gastroesophageal reflux disease

N-snps represent the number of SNPS in each loci range

*represent the portion used for co-locating positive results

### Tissue-specific Enrichment of SNP Heritability

**Fig. 4:**
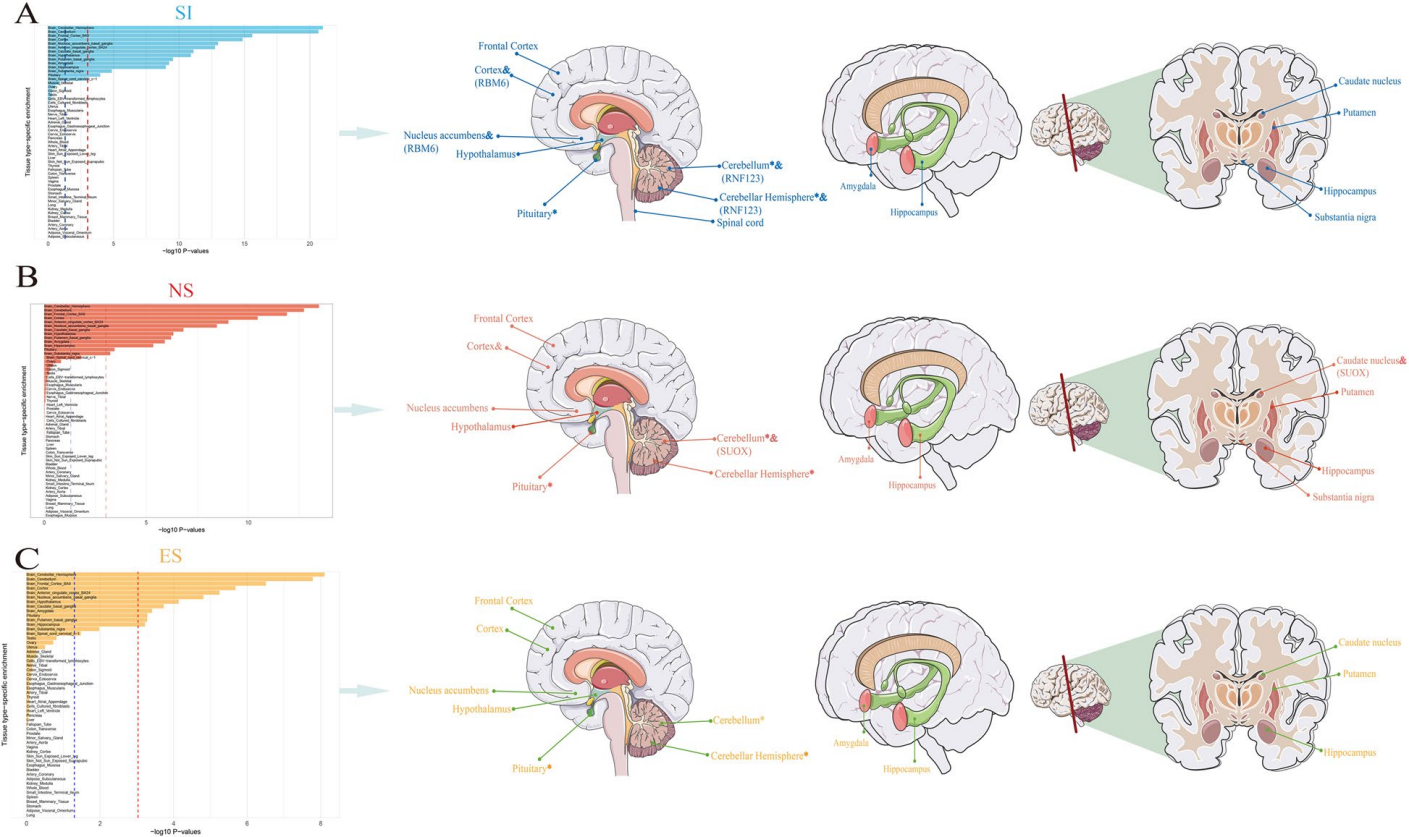
Tissue-Specific Enrichment Diagram of SNP Heritability between SI, NS, ES, and GERD. Tissue-specific enrichment diagrams for smoking behavior (SI, NS, ES) and GERD, along with an illustration of co-enriched loci in the brain. The left blue dashed line represents the threshold for statistical significance (0.05), while the red dashed line represents the Bonferroni-corrected significance threshold (0.05/53 = 9.43E-04). Different colors annotate brain tissues with significant co-enriched loci after Bonferroni correction. **A** Blue font and solid lines represent brain tissues co-enriched between SI and GERD; **B** Orange-red font and solid lines represent brain tissues co-enriched between NS and GERD; **C** Yellow font and solid lines represent brain tissues co-enriched between ES and GERD. All brain tissues marked with “*” indicate sites enriched for all five smoking behavior and GERD, while “&” signifies tissues identified through summary data-based Mendelian randomization (SMR) analysis for potential functional genes, with these genes being annotated accordingly. SI: Smoking initiation; NS: Never Smoking; ES: Ever Smoking; GERD: Gastroesophageal reflux disease

**Fig. 5:**
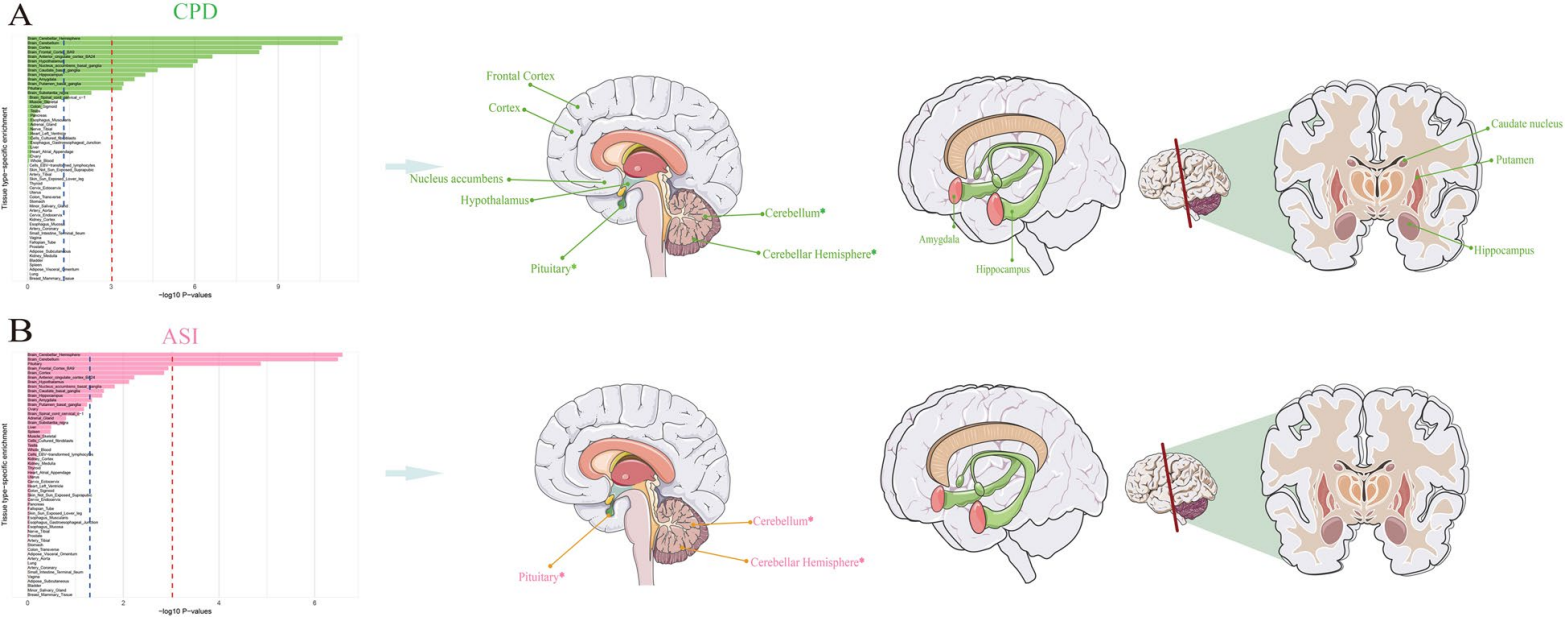
Tissue-Specific Enrichment Diagram of SNP Heritability between CPD, ASI, and GERD. Tissue-specific enrichment diagrams for smoking behavior (CPD, ASI) and GERD, along with an illustration of co-enriched loci in the brain. The left blue dashed line represents the threshold for statistical significance (0.05), while the red dashed line represents the Bonferroni-corrected significance threshold (0.05/53 = 9.43E-04). Different colors annotate brain tissues with significant co-enriched loci after Bonferroni correction. **A** Green font and solid lines represent brain tissues co-enriched between CPD and GERD; **B** Pink font and solid lines represent brain tissues co-enriched between ASI and GERD. All brain tissues marked with “*” indicate sites enriched for all five smoking behavior and GERD. CPD: Cigarettes smoked per day; ASI: Age of smoking initiation; GERD: Gastroesophageal reflux disease

After adjusting the baseline model, we identified multiple brain tissues showing significant SNP-heritability enrichment shared between smoking behavior and GERD. In terms of smoking-related traits, SNPs associated with SI, NS, ES, CPD, and ASI were observed to be specifically enriched in 14, 13, 13, 12, and 3 different brain regions, respectively, while for GERD, SNPs were enriched in 10 different brain regions. Interestingly, all five traits showed enrichment primarily in the Cerebellar Hemisphere and Cerebellum (Figs. 4 and 5, Supplementary Material Table S8/ Figure S7). We utilized the SMR method to identify putative functional genes underlying smoking behavior and GERD, leveraging joint analysis of GWAS summary data for the five smoking-related traits, GERD, and eQTL summary data from GTEx (showing SNP-heritability enrichment in five smoking-related traits and GERD across brain tissues). The results revealed statistically associated putative functional genes between SI, NS, and GERD across different tissues showing significant SNP-heritability enrichment. Four tissues were found to be co-enriched for SI and GERD, with GMPPB and RNF123 co-enriched in the Cerebellar Hemisphere and Cerebellum, while RBM6 co-enriched in the Cortex and Nucleus accumbens. Additionally, SUOX was identified as a gene co-enriched in the Caudate nucleus, Cerebellum, and Cortex, shared between NS and GERD. These results also passed the HEIDI-outlier test (*P* > 0.05) (Table 3).

**Table 3:** Shared significant SMR associations for smoking behavior and GERD in enriched tissues

Exposure-Outcome	Tissue (Brain)	Functional Gene	Exposure						Outcome				
			**Top** **SNP**	**Βeta** **SMR**	**Se** **SMR**	***P***-**val SMR**	***P***-**val** **HEIDI**	**BH**	**Βeta** **SMR**	**Se** **SMR**	***P***-**val SMR**	***P***-**val** **HEIDI**	**BH**
SI-GERD	Cerebellar Hemisphere	GMPPB	rs62262722	0.03	0.01	1.19E-06	0.050499	4.48E-06	-0.11	0.02	1.25E-06	0.106184	1.88E-06
	Cerebellum	RNF123	rs62260755	0.09	0.02	1.56E-05	0.957791	1.94E-05	-0.12	0.02	4.18E-11	0.05089	2.23E-10
	Cortex	RBM6	rs11919418	-0.06	0.01	2.91E-05	0.393813	4.19E-05	0.08	0.01	7.41E-14	0.252924	8.89E-13
	Nucleus accumbens	RBM6	rs12632110	-0.09	0.02	2.01E-05	0.465134	2.28E-05	0.11	0.02	8.94E-13	0.219377	8.05E-12
NS-GERD	Caudate nucleus	SUOX	rs10876864	-0.02	0.01	2.81E-05	0.139845	3.59E-05	-0.11	0.02	4.64E-06	0.233402	6.85E-06
	Cerebellum	SUOX	rs10876864	-0.01	0.00	6.84E-07	0.087019	1.88E-06	-0.05	0.01	7.59E-10	0.254894	2.43E-09
	Cortex	SUOX	rs1131017	-0.02	0.01	3.67E-05	0.082014	4.45E-05	-0.11	0.02	1.19E-05	0.180958	1.78E-05

*SMR* Summary data-based Mendelian randomization, *SI* Smoking initiation, *NS* Never Smoking, *GERD* Gastroesophageal reflux disease, *BH* Benjamini & Hochberg, *HEIDI* Heterogeneity in dependent instrument

### Causal associations

**Fig. 6:**
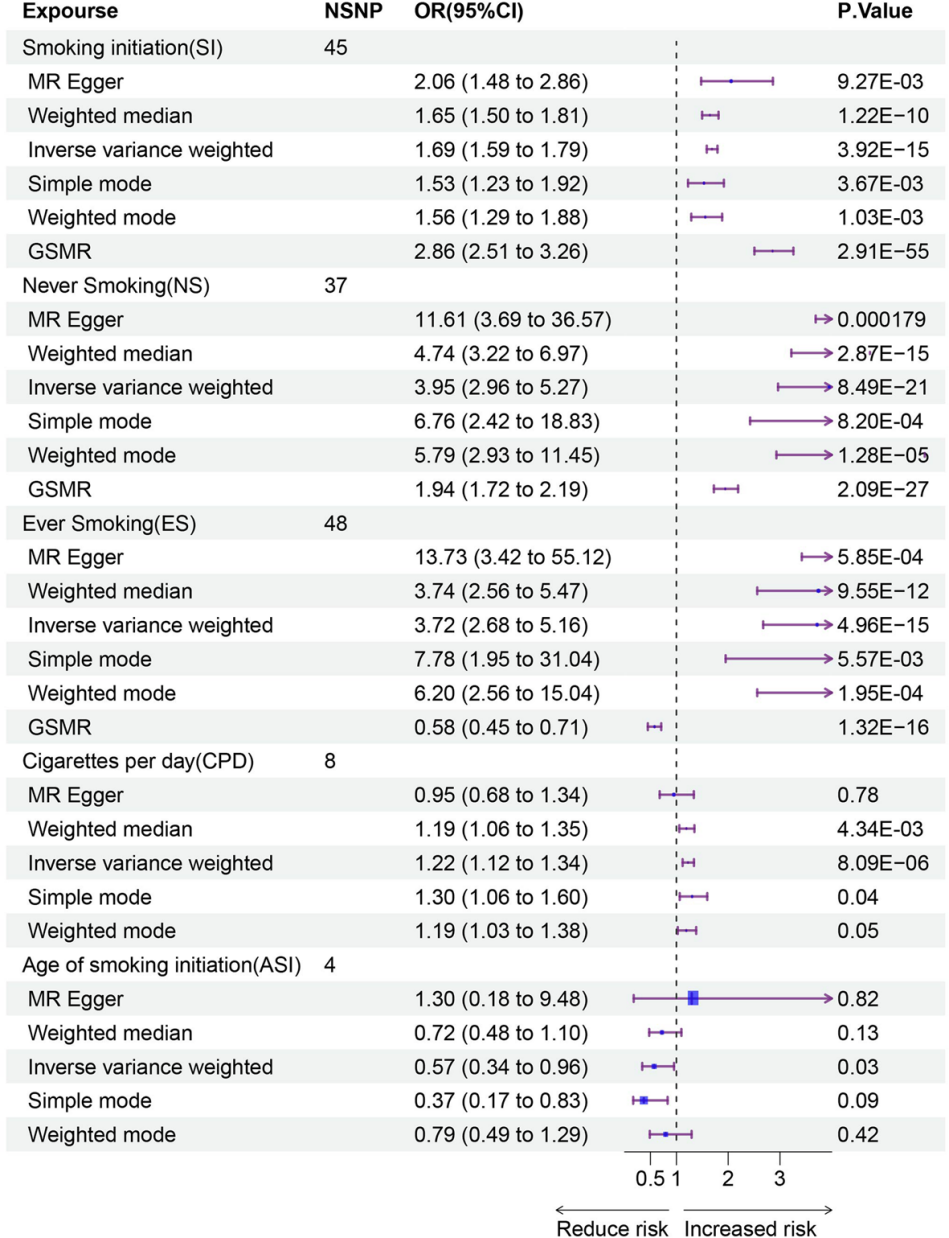
Mendelian randomization analysis between smoking behavior and GERD. Using “inverse variance weighting (IVW)” as the primary analysis method, with MR-eggr regression, weighted median (WM), weighted model, and generalized summary-data-based Mendelian randomization (GSMR) analysis as supplements. Since GSMR typically requires the number of instrumental variables (IVs) for exposure to be greater than 10, CPD and ASI were not included in this analysis. MR: Mendelian randomization; SI: Smoking initiation; NS: Never Smoking; ES: Ever Smoking; CPD: Cigarettes smoked per day; ASI: Age of smoking initiation; GERD: Gastroesophageal reflux disease.....

Having delved into the shared genetic background between smoking behavior and GERD, we further explored the potential causal effects between them through MR analysis. IV were chosen after evaluating based on the three assumptions of MR. The expected F-statistics for the five smoking behavior as exposures were 13.51 (SI), 9.73 (NS), 9.61 (ES), 29.55 (CPD), and 28.35 (ASI). With the maximum sample overlap between these five smoking behavior and GERD ranging from 0.11 to 0.20, the type 1 error rate consistently remained at 0.05, meeting the criteria for the chosen IVs (Supplementary Material—Table S9/10). Then, we found evidence supporting a causal relationship from smoking behavior to GERD, where SI, ES, and CPD were associated with an increased risk of GERD (SI: P_IVW = 3.92E-15; ES: P_IVW = 4.96E-15; CPD: P_IVW = 8.09E-06), while NS and ASI showed the opposite (NS: P_IVW = 4.96E-15; ASI: P_IVW = 0.03, Fig. 6). Neither SI nor CPD exhibited heterogeneity, and all results did not show evidence of horizontal pleiotropy (Table 4). Leave-one-out analysis also indicated that these effects were not driven by any single SNP (Supplementary Material—Figure S8-12). The MVMR results also indicate that the alcohol factor does not influence the causal relationship between these smoking behavior and GERD (Supplementary Material—Table S11). Finally, when using GERD data from FinnGen as the outcome variable, above MR results remained consistent, demonstrating the robustness of the findings (Supplementary Material—Figure S13, Table S12).

**Table 4:** Sensitivity analysis of Mendelian randomization

Exposure	Outcome	Cochran Q value	Q-pval	MR-Egger Intercept	*P-*value	MR-PRESSO *P*-value
SI	GERD	39.65	0.66	-5.14E-03	0.24	0.23
NS		52.56	0.04	-7.77E-03	0.07	0.0045
ES		119.96	2.65E-08	9.5E-03	0.06	< 2E-04
CPD		6.93	0.44	0.01	0.18	0.16
ASI		10.60	0.01	-0.02	0.49	0.07

*SI* Smoking initiation, *NS* Never Smoking, *ES* Ever Smoking, *CPD* Cigarettes smoked per day, *ASI* Age of smoking initiation, *GERD* Gastroesophageal reflux disease, *MR-PRESSO* Mendelian Randomization Pleiotropy RESidual Sum and Outlier

## Discussion

To the best of our knowledge, this is the first study to leverage a whole-genome cross-trait analysis to systematically assess the shared genetic underpinnings behind smoking behavior and GERD. Our research offers new genetic insights: firstly, associations between smoking behavior (SI, NS, ES, CPD, ASI) and GERD are present across multiple specific loci. Secondly, three novel pleiotropic SNPs (rs4382592, rs200968, rs1510719) have been identified across these loci, existing between SI, NS, ES, ASI, and GERD, mapping the genes MED27, HIST1H2BO, and MAML3 as potential pleiotropic genes. Furthermore, common enrichment of the five smoking behavior and GERD in brain tissues, predominantly in the Cerebellar Hemisphere and Cerebellum, has been observed. In the Cerebellar Hemisphere, Cerebellum, and Cortex/Nucleus accumbens, enriched for SI and GERD, GMPPB, RNF123, and RBM6 were identified as potential functional genes, while SUOX was identified in the Caudate nucleus, Cerebellum, and Cortex, enriched for NS and GERD. These genes have not been previously revealed. Lastly, MR analysis indicates a causal relationship of the five smoking behavior with GERD, with SI, ES, and higher CPD increasing the risk of GERD, whereas NS and higher ASI may reduce the risk. Overall, our study robustly demonstrates the genetic correlation between smoking behavior and GERD.

Previous studies have indicated a significant genetic relationship between GERD and smoking. Twin studies suggest that genetic factors contribute up to 31% to the etiology of GERD-related symptoms [27]. Additionally, there is a wide variation in individual susceptibility to tobacco, with maternal inheritance of tobacco preference being a key factor in nicotine addiction [28]. Our study aims to provide novel insights into this area. Firstly, we found that the locus chr2(p16.1) consistently takes precedence among the significantly associated gene loci between SI, NS, ES, and GERD. Notably, all smoking behavior examined in this study exhibited significant associations with GERD within this loci. Furthermore, the loci chr18(q12.3) and chr11(q23.1- q23.2), identified as the most significantly associated loci between CPD, ASI, and GERD, also showed significant associations between all smoking behavior and GERD. These findings suggest that further research into these three leading loci is warranted, despite the lack of reported associations with smoking or GERD. Moreover, through a joint cross-trait GWAS meta-analysis, we identified eight novel pleiotropic SNPs mapping to six genes within the gene loci significantly associated with both smoking behavior and GERD. Among them, rs4382592, rs200968, and rs1510719 passed the co-localization analysis and mapped to MED27, HIST1H2BO, and MAML3 genes, respectively. Nicotine in tobacco can induce relaxation of the lower esophageal sphincter (LES) by blocking cholinergic receptors, leading to a decrease in LES pressure [29], which results in a rapid decrease in LES pressure shortly after smoking [30, 31]. The combination of lower baseline LES pressure and abnormally high rates of transient, non-swallow-related LES relaxation are the main reasons for gastric acid reflux [32]. The disruption of muscle tone caused by MED27 may play a role in the complex relationship between smoking behavior and gastroesophageal reflux, warranting further investigation. No association between HIST1H2BO, MAML3 (Mastermind Like Transcriptional Coactivator 3), and GERD or smoking has been found, with current reports focusing on cancer [33, 34]. Although colocalization analysis did not further confirm the association between TRAF3 (TNF Receptor Associated Factor 3), DRD2 (Dopamine Receptor D2), FOXP2 (Forkhead box protein P2), and GERD, they may have potential roles in the association between smoking behavior and GERD. For instance, DRD2 encodes a dopamine receptor, highly relevant to substance dependence. Current pharmacogenetic research suggests an association between the DRD2 gene and the response to smoking cessation medications, as well as smoking behavior itself [35], and we also look forward to developing relevant medications for the prevention or treatment of GERD.

Tissue-specific enrichment results demonstrate varying degrees of enrichment of all smoking behavior and GERD across different brain tissues, with the Cerebellum and Cortex being the most significant. We also identified RNF123, RBM6, and SUOX as potential functional genes in these brain tissues through SMR analysis between SI, NS, and GERD, with RBM6 also identified in the Nucleus accumbens enriched for SI and GERD, and GMPPB additionally identified in the Cerebellar Hemisphere region. These results suggest that smoking behavior might also influence GERD through effects on brain tissues, where the addiction mechanism of the Nucleus accumbens concerning smoking is notable [36], potentially serving as a breakthrough in the association with GERD. The ongoing development of the "brain-gut" axis in the field of psychogastroenterology greatly aids in understanding, developing, and treating chronic digestive diseases [37]. The integration between the gut and the exogenous autonomic nervous system through excitatory vagal pathways and sympathetic nerve pathways is considered key to maintaining LES function [38], with some existing studies providing a basis for our results. For example, Shaker R [39] and Wang K [40] demonstrated increased insular cortex activity during esophageal stimulation in patients with GERD, indicating a sensitive esophageal-cortical neural axis. The significant impact of smoking on the neuroendocrine system may thus link smoking behavior and GERD through neural transmission disorders, making related brain tissues of interest.

Our study has certain limitations. First, our data are derived from individuals of European descent, which, while reducing the impact of population stratification and genetic heterogeneity, may also limit the generalizability of our findings to other ethnicities. Additionally, this factor contributes to potential sample overlap; however, after multiple rounds of validation, there is reason to believe that sample overlap does not have a decisive impact on the study conclusions. Second, in the MR analysis of ES and GERD, GSMR results and IVW show inconsistency, possibly due to the introduction of HEIDI and reference to the 1000 Genomes Project, warranting further individual analysis in the future. Finally, due to limitations in the applicability of statistical methods, the analysis of SNPs on sex chromosomes was not included in this study, which could be further explored in future research to perform gender difference analyses.

## Conclusion

In summary, we have identified significant global and local genetic correlations and causal relationships between five smoking behavior and GERD, with multiple associated loci identified, and further pinpointed three new pleiotropic SNPs and their mapped genes. Additionally, various brain tissues have been linked to both smoking behavior and GERD, with four potential functional genes identified in brain tissues specifically enriched for SI, NS, and GERD. These novel findings can help better elucidate the inherent genetic connections and shared genetic mechanisms between smoking behavior and GERD.

### Supplementary Information

Supplementary Material 1.

## Data Availability

Data is provided within the manuscript or supplementary information files.

## References

[1] Vakil N, van Zanten SV, Kahrilas P, Dent J, Jones R, G. Global Consensus (2006). The montreal definition and classification of gastroesophageal reflux disease: a global evidence-based consensus. Am J Gastroenterol..

[2] Iwakiri  KY, Fujiwara  N, Manabe  E, Ihara  S, Kuribayashi  J, Akiyama  T, Kondo  H, Yamashita  N, Ishimura  Y, Kitasako Y (2022). Evidence-based clinical practice guidelines for gastroesophageal reflux disease 2021. J Gastroenterol.

[3] El-Serag HB, Sweet S, Winchester CC, Dent  J (2014). Update on the epidemiology of gastro-oesophageal reflux disease: a systematic review. Gut.

[4] Li LF, Chan RL, Lu L, Shen J, Zhang L, Wu WK, Wang L, Hu T, Li MX, Cho CH (2014). Cigarette smoking and gastrointestinal diseases: the causal relationship and underlying molecular mechanisms (review). Int J Mol Med..

[5] Etemadi  A, Gandomkar  A, Freedman ND, Moghadami M, Fattahi MR, Poustchi H, Islami F, Boffetta P, Dawsey SM, Abnet CC (2017). The association between waterpipe smoking and gastroesophageal reflux disease. Int J Epidemiol.

[6] Soroush A, Malekzadeh R, Roshandel G, Khoshnia M, Poustchi H, Kamangar F, Brennan P, Boffetta P, Dawsey SM, Abnet CC (2023). Sex and smoking differences in the association between gastroesophageal reflux and risk of esophageal squamous cell carcinoma in a high-incidence area: golestan cohort study. Int J Cancer.

[7] Nilsson M, Johnsen R, Ye W, Hveem K, Lagergren J (2004). Lifestyle related risk factors in the aetiology of gastro-oesophageal reflux. Gut.

[8] Fujiwara Y, Kubo M, Kohata Y, Machida H, Okazaki H, Yamagami H, Tanigawa T, Watanabe K, Watanabe T, Tominaga K (2011). Cigarette smoking and its association with overlapping gastroesophageal reflux disease, functional dyspepsia, or irritable bowel syndrome. Intern Med.

[9] Kohata Y, Fujiwara Y, Watanabe T, Kobayashi M, Takemoto Y, Kamata N, Yamagami H, Tanigawa T, Shiba M, Watanabe T (2016). Long-term benefits of smoking cessation on gastroesophageal reflux disease and health-related quality of life. PLoS One.

[10] Ness-Jensen E, Lindam A, Lagergren J, Hveem K (2014). Tobacco smoking cessation and improved gastroesophageal reflux: a prospective population-based cohort study: the hunt study. Am J Gastroenterol.

[11] Mavromatis LA, Rosoff DB, Cupertino RB, Garavan H, Mackey S, Lohoff FW (2022). Association between brain structure and alcohol use behaviors in adults: a mendelian randomization and multiomics study. JAMA Psychiatry.

[12] Liu M, Jiang Y, Wedow R, Li Y, Brazel DM, Chen F, Datta G, Davila-Velderrain J, McGuire D, Tian C (2019). Association studies of up to 1.2 million individuals yield new insights into the genetic etiology of tobacco and alcohol use. Nat Genet.

[13] Ong JS, An J, Han X, Law MH, Nandakumar P, Schumacher J, Gockel I, Bohmer A, , t and Me Research, c. Esophageal cancer (2022). Multitrait genetic association analysis identifies 50 new risk loci for gastro-oesophageal reflux, seven new loci for barrett's oesophagus and provides insights into clinical heterogeneity in reflux diagnosis. Gut.

[14] Bulik-Sullivan B, Finucane HK, Anttila V, Gusev A, Day FR, Loh PR, Duncan L, ReproGen C, C. Psychiatric Genomics, C. Genetic Consortium for Anorexia Nervosa of the Wellcome Trust Case Control (2015). An atlas of genetic correlations across human diseases and traits. Nat Genet.

[15] Sheerin  CM, Bountress KE, Meyers JL, Saenz de Viteri SS, Shen H, Maihofer AX, Duncan LE, Amstadter AB (2020). Shared molecular genetic risk of alcohol dependence and posttraumatic stress disorder (ptsd). Psychol Addict Behav.

[16] Gazal S, Finucane HK, Furlotte NA, Loh PR, Palamara PF, Liu X, Schoech A, Bulik-Sullivan B, Neale BM, Gusev A (2017). Linkage disequilibrium-dependent architecture of human complex traits shows action of negative selection. Nat Genet.

[17] Shi H, Mancuso N, Spendlove S, Pasaniuc B (2017). Local genetic correlation gives insights into the shared genetic architecture of complex traits. Am J Hum Genet.

[18] Yoshida GM, Yanez JM (2021). Multi-trait gwas using imputed high-density genotypes from whole-genome sequencing identifies genes associated with body traits in nile tilapia. BMC Genomics.

[19] Yang Y, Musco H, Simpson-Yap S, Zhu Z, Wang Y, Lin X, Zhang J, Taylor B, Gratten J, Zhou Y (2021). Investigating the shared genetic architecture between multiple sclerosis and inflammatory bowel diseases. Nat Commun.

[20] Turley P, Walters RK, Maghzian O, Okbay A, Lee JJ, Fontana MA, Nguyen-Viet TA, Wedow R, Zacher M, Furlotte NA (2018). Multi-trait analysis of genome-wide association summary statistics using mtag. Nat Genet.

[21] Zhu Z, Hasegawa K, Camargo CA, Liang L (2021). Investigating asthma heterogeneity through shared and distinct genetics: Insights from genome-wide cross-trait analysis. J Allergy Clin Immunol.

[22] Storm CS, Kia DA, Almramhi MM, Bandres-Ciga S, Finan C, Hingorani AD, Wood NW, International Parkinson's Disease Genomics C (2021). Finding genetically-supported drug targets for parkinson's disease using mendelian randomization of the druggable genome. Nat Commun.

[23] Finucane HK, Reshef YA, Anttila V, Slowikowski K, Gusev A, Byrnes A, Gazal S, Loh PR, Lareau C, Shoresh N (2018). Heritability enrichment of specifically expressed genes identifies disease-relevant tissues and cell types. Nat Genet.

[24] Zhu Z, Zhang F, Hu H, Bakshi A, Robinson MR, Powell JE, Montgomery GW, Goddard ME, Wray NR, Visscher PM (2016). Integration of summary data from gwas and eqtl studies predicts complex trait gene targets. Nat Genet.

[25] Zhu Z, Zheng Z, Zhang F, Wu Y, Trzaskowski M, Maier R, Robinson MR, McGrath JJ, Visscher PM, Wray NR (2018). Causal associations between risk factors and common diseases inferred from gwas summary data. Nat Commun.

[26] Sanderson, E. "Multivariable mendelian randomization and mediation." Cold Spring Harb Perspect Med. 2011;11. 10.1101/cshperspect.a038984. https://www.ncbi.nlm.nih.gov/pubmed/32341063. 10.1101/cshperspect.a038984 PMC7849347 32341063

[27] Cameron AJ, Lagergren J, Henriksson C, Nyren O, Locke GR, Pedersen NL (2002). Gastroesophageal reflux disease in monozygotic and dizygotic twins. Gastroenterology.

[28] Sullivan PF, Kendler KS (1999). The genetic epidemiology of smoking. Nicotine Tob Res.

[29] Ellis FG, Kauntze R, Trounce JR (1960). The innervation of the cardia and lower oesophagus in man. Br J Surg.

[30] Chattopadhyay DK, Greaney MG, Irvin TT (1977). Effect of cigarette smoking on the lower oesophageal sphincter. Gut.

[31] Dennish GW, Castell DO (1971). Inhibitory effect of smoking on the lower esophageal sphincter. N Engl J Med.

[32] Maret-Ouda J, Markar SR, Lagergren J (2020). Gastroesophageal reflux disease: a review. JAMA.

[33] Alzofon N, Koc K, Panwell K, Pozdeyev N, Marshall CB, Albuja-Cruz M, Raeburn CD, Nathanson KL, Cohen DL, Wierman ME (2021). Mastermind like transcriptional coactivator 3 (maml3) drives neuroendocrine tumor progression. Mol Cancer Res.

[34] Weber L, Massberg D, Becker C, Altmuller J, Ubrig B, Bonatz G, Wolk G, Philippou S, Tannapfel A, Hatt H (2018). Olfactory receptors as biomarkers in human breast carcinoma tissues. Front Oncol.

[35] Munafo MR, Timpson NJ, David SP, Ebrahim S, Lawlor DA (2009). Association of the drd2 gene taq1a polymorphism and smoking behavior: a meta-analysis and new data. Nicotine Tob Res.

[36] Markunas CA, Semick SA, Quach BC, Tao R, Deep-Soboslay A, Carnes MU, Bierut LJ, Hyde TM, Kleinman JE, Johnson EO (2021). Genome-wide DNA methylation differences in nucleus accumbens of smokers vs. Nonsmokers. Neuropsychopharmacology.

[37] Keefer L, Palsson OS, Pandolfino JE (2018). Best practice update: incorporating psychogastroenterology into management of digestive disorders. Gastroenterology.

[38] Campo SM, Capria A, Antonucci F, Martino G, Ciamei A, Rossini PM, Bologna E, Cannata D (2001). Decreased sympathetic inhibition in gastroesophageal reflux disease. Clin Auton Res.

[39] Shaker R (2007). Gastroesophageal reflux disease: beyond mucosal injury. J Clin Gastroenterol.

[40] Wang KLP, Dua LP, Zeng XZ, Liu JY, Xu-Chu W (2011). Differences in cerebral response to esophageal acid stimuli and psychological anticipation in gerd subtypes--an fmri study. BMC Gastroenterol.

