# Supplementary material for: Genetic correlation between smoking behavior and gastroesophageal reflux disease: insights from integrative multi-omics data
Source: BMC Genomics. 2024 Jun 27;25:642. doi: 10.1186/s12864-024-10536-3 (PMC11212162; doi:10.1186/s12864-024-10536-3)
Supplement: Supplementary file 1 — Supplementary Material 1. [file 12864_2024_10536_MOESM1_ESM.docx]

Genetic Correlation Between Smoking Behavior and Gastroesophageal Reflux Disease: Insights from Integrative Multi-Omics Data

[Table S1: Additional definitions for the five smoking and alcohol behavior in the questionnaire scale 3](#_Toc167050085)

[Table S2: Local genetic correlation between SI and GERD 5](#_Toc167050086)

[Table S3: Local genetic correlation between NS and GERD 11](#_Toc167050087)

[Table S4: Local genetic correlation between ES and GERD 16](#_Toc167050088)

[Table S5: Local genetic correlation between CPD and GERD 19](#_Toc167050089)

[Table S6: Local genetic correlation between ASI and GERD 22](#_Toc167050090)

[Table S7: Cross-trait GWAS meta-analysis 24](#_Toc167050091)

[Figure S1. Visualization of Genome-wide Loci Distribution for GERD 31](#_Toc167050092)

[Figure S2. Visualization of Genome-wide Loci Distribution for SI and Mapped Genes of "pleiotropic SNPs" 32](#_Toc167050093)

[Figure S3. Visualization of Genome-wide Loci Distribution for NS and Mapped Genes of "pleiotropic SNPs" 33](#_Toc167050094)

[Figure S4. Visualization of Genome-wide Loci Distribution for ES and Mapped Genes of "pleiotropic SNPs" 34](#_Toc167050095)

[Figure S5. Visualization of Genome-wide Loci Distribution for CPD 35](#_Toc167050096)

[Figure S6. Visualization of Genome-wide Loci Distribution for ASI and Mapped Genes of "pleiotropic SNPs" 36](#_Toc167050097)

[Table S8: Tissue specific enrichment for five smoking behavior and GERD 37](#_Toc167050098)

[Figure S7: Visualization of tissue-specific enrichment of genetic heritability of GERD's SNP 50](#_Toc167050099)

[Table S9: Statistical differences due to potential sample overlap rates 51](#_Toc167050100)

[Table S10: Genetic instrumental variables for five smoking behavior used in Mendelian randomization analysis 54](#_Toc167050101)

[Figure S8: Sensitivity analysis of Smoking initiation and GERD 61](#_Toc167050102)

[Figure S9: Sensitivity analysis of Never smoking and GERD 62](#_Toc167050103)

[Figure S10: Sensitivity analysis of Ever Smoking and GERD 63](#_Toc167050104)

[Figure S11: Sensitivity analysis of Age of smoking initiation and GERD 64](#_Toc167050105)

[Figure S12: Sensitivity analysis of Cigarettes smoked per day and GERD 65](#_Toc167050106)

[Table S11: Multivariate Mendelian randomization of Smoking behavior and GERD after adjustment for alcohol 66](#_Toc167050107)

[Figure S13: Mendelian Randomization Analysis between Smoking Behavior and GERD (FinnGen) 67](#_Toc167050108)

[Table S12: Sensitivity analysis of Mendelian randomization (FinnGen) 68](#_Toc167050109)

# Table S1: Additional definitions for the five smoking and alcohol behavior in the questionnaire scale

| **Age of Initiation of Smoking (ASI)** | **Cigarettes per Day (CPD)** | **Smoking Initiation (SI)/**  **Ever smoking (ES)** | **Never smoking (NS)** | **Drinks per Week (DPW)** |
| --- | --- | --- | --- | --- |
| 1. Age at which an individual started smoking cigarettes regularly | 1. Defined as the average number of cigarettes smoked per day, either as a current smoker or former smoker, and whether self-rolled or manufactured are smoked (most studies did not distinguish). Individuals who either never smoked, or for whom there is no available data (e.g., someone was a former smoker, but for whom former smoking was never assessed) were set to missing. | 1. This is a binary phenotype. Any participant reporting ever being a regular smoker in their life were coded “2”, while any participant who reported never being a regular smoker in their life were coded “1”. | 1. This is a binary phenotype. Any participant reporting ever being a regular smoker in their life were coded “2”, while any participant who reported never being a regular smoker in their life were coded “1”. | 1. Defined as the average number of drinks a participant reported drinking each week, aggregated across all types of alcohol. If a study recorded binned response ranges (e.g., 1-4 drinks per week, 5-10 drinks per week) we used the midpoint of the range. For example, if an individual reported 1-5 drinks per week, we assume they drank 2.5 drinks per week on average. |
| 2. Does not include information about pipes/cigars/chew, or other non-cigarette forms of tobacco use. | 2. For studies that collected a quantitative measure of cigarettes per day, where the respondent is free to provide any integer (e.g., 13 cigarettes per day) responses were binned as follows.  a. 1 = 1-5  b. 2 = 6-15  c. 3 = 16-25  d. 4 = 26-35  e. 5 = 36+ | 2. Does not include information about pipes/cigar/chew, or other non-cigarette forms of tobacco use. | 2. Does not include information about pipes/cigar/chew, or other non-cigarette forms of tobacco use. | 2. This was measured in a variety of ways.  a. In the past week, how many alcoholic beverages did you have?  b. Thinking about the past year, on the average how many drinks did you have each week? |
| 3. Measured in a variety of ways:  a. At what age did you begin smoking regularly?  b. How long have you smoked? combined with What is your current age? | 3. For studies with pre-defined bins, the pre-defined bins were used. | 3. This phenotype was measured in a variety of ways.  a. Have you smoked over 100 cigarettes over the course of your life?  b. Have you ever smoked every day for at least a month?  c. Have you ever smoked regularly? |  | 3. This phenotype was left-anchored at 1 and log-transformed prior to analysis, in order to prevent outliers from having undue leverage on analyses. |
|  | 4. Does not include information about pipes/cigars/chew, or other non-cigarette forms of tobacco use. |  |  |  |

The phenotypes related to smoking behavior (including alcohol-related phenotypes added in the third question) from the GSCAN Consortium were defined by a working group composed of Drs. Laura J. Bierut, Marilyn C. Cornelis, David A. Hinds, Jaakko Kaprio, Eric Jorgenson, Dajiang J. Liu, Matt McGue, Marcus R. Munafo, Scott Vrieze, and Luisa Zuccolo. The selection of phenotypes was based on the availability of a large number of phenotypes in participating studies, phenotypes from previous genetic association studies, and clinically relevant information collected after multiple conference calls to choose and define the phenotypes.

Liu M, Jiang Y, Wedow R, et al. Association studies of up to 1.2 million individuals yield new insights into the genetic etiology of tobacco and alcohol use. Nat Genet. 2019 Feb;51(2):237-244. doi: 10.1038/s41588-018-0307-5. Epub 2019 Jan 14.

# Table S2: Local genetic correlation between SI and GERD

| **chr** | **start** | **end** | **num_snp** | **local_rhog** | **var** | **se** | **z** | **p** |
| --- | --- | --- | --- | --- | --- | --- | --- | --- |
| 2 | 58297315 | 60292000 | 1221 | 0.00049605 | 2.65E-09 | 5.15E-05 | 9.6289 | 6.04E-22 |
| 1 | 43758457 | 44969183 | 586 | 0.00046751 | 2.95E-09 | 5.43E-05 | 8.6092 | 7.36E-18 |
| 7 | 116780178 | 118351581 | 834 | 0.00035019 | 1.88E-09 | 4.34E-05 | 8.0662 | 7.25E-16 |
| 11 | 112459488 | 114257728 | 1392 | 0.000503 | 4.28E-09 | 6.55E-05 | 7.6842 | 1.54E-14 |
| 1 | 76728135 | 79661198 | 1844 | 0.00033632 | 2.21E-09 | 4.71E-05 | 7.1476 | 8.83E-13 |
| 18 | 39892648 | 42922106 | 1994 | 0.00032507 | 2.21E-09 | 4.70E-05 | 6.9137 | 4.72E-12 |
| 12 | 119754110 | 122007651 | 1033 | 0.00034548 | 2.51E-09 | 5.01E-05 | 6.8993 | 5.23E-12 |
| 6 | 110304247 | 112345014 | 1065 | 0.00033346 | 2.46E-09 | 4.96E-05 | 6.7225 | 1.79E-11 |
| 6 | 25684587 | 26791233 | 844 | 0.00041058 | 3.76E-09 | 6.14E-05 | 6.6921 | 2.20E-11 |
| 9 | 81079055 | 82590928 | 1182 | 0.00034645 | 2.72E-09 | 5.21E-05 | 6.6438 | 3.06E-11 |
| 1 | 71684405 | 74326907 | 1383 | 0.00039511 | 3.55E-09 | 5.96E-05 | 6.6302 | 3.35E-11 |
| 1 | 115880593 | 118839067 | 1522 | 0.00028054 | 1.81E-09 | 4.25E-05 | 6.6005 | 4.10E-11 |
| 19 | 18409862 | 19877471 | 480 | 0.00043101 | 4.27E-09 | 6.53E-05 | 6.5982 | 4.16E-11 |
| 2 | 10133 | 1781022 | 1222 | 0.00031256 | 2.29E-09 | 4.78E-05 | 6.5341 | 6.40E-11 |
| 2 | 21050490 | 23341383 | 1273 | 0.00034924 | 2.86E-09 | 5.35E-05 | 6.5324 | 6.47E-11 |
| 7 | 25909555 | 28360309 | 1825 | 0.00025141 | 1.51E-09 | 3.88E-05 | 6.4736 | 9.57E-11 |
| 2 | 173138905 | 175588929 | 1489 | 0.00029776 | 2.12E-09 | 4.61E-05 | 6.4642 | 1.02E-10 |
| 4 | 139556511 | 141084631 | 758 | 0.00039581 | 3.75E-09 | 6.12E-05 | 6.4641 | 1.02E-10 |
| 10 | 102949239 | 104380410 | 482 | 0.00029932 | 2.15E-09 | 4.64E-05 | 6.4572 | 1.07E-10 |
| 1 | 154770403 | 156336133 | 435 | 0.00025123 | 1.54E-09 | 3.93E-05 | 6.3946 | 1.61E-10 |
| 2 | 78079264 | 80050403 | 1849 | 0.00028748 | 2.10E-09 | 4.58E-05 | 6.2801 | 3.38E-10 |
| 7 | 113712387 | 116780178 | 1458 | 0.00033981 | 2.94E-09 | 5.42E-05 | 6.2702 | 3.61E-10 |
| 2 | 50818928 | 52491133 | 1517 | 0.00031858 | 2.61E-09 | 5.11E-05 | 6.2393 | 4.40E-10 |
| 8 | 59728100 | 62329099 | 1672 | 0.00031997 | 2.69E-09 | 5.18E-05 | 6.1722 | 6.73E-10 |
| 9 | 134127888 | 135298842 | 653 | 0.00030823 | 2.56E-09 | 5.06E-05 | 6.0887 | 1.14E-09 |
| 18 | 77149991 | 78017158 | 439 | 0.0003029 | 2.54E-09 | 5.04E-05 | 6.0096 | 1.86E-09 |
| 1 | 216243634 | 218705513 | 2116 | 0.00022519 | 1.41E-09 | 3.76E-05 | 5.9893 | 2.11E-09 |
| 12 | 61123729 | 64032461 | 2225 | 0.00029531 | 2.44E-09 | 4.94E-05 | 5.9786 | 2.25E-09 |
| 5 | 175450595 | 177251507 | 468 | 0.00023323 | 1.53E-09 | 3.92E-05 | 5.9549 | 2.60E-09 |
| 4 | 132954033 | 134628434 | 1044 | 0.00023405 | 1.55E-09 | 3.94E-05 | 5.9391 | 2.87E-09 |
| 8 | 90638162 | 93554386 | 1421 | 0.00031545 | 2.82E-09 | 5.31E-05 | 5.9379 | 2.89E-09 |
| 6 | 164383521 | 165586864 | 1187 | 0.00026386 | 1.98E-09 | 4.45E-05 | 5.9285 | 3.06E-09 |
| 13 | 65200602 | 67844453 | 1569 | 0.00030237 | 2.61E-09 | 5.11E-05 | 5.9196 | 3.23E-09 |
| 16 | 53382572 | 55903774 | 1947 | 0.00027927 | 2.23E-09 | 4.72E-05 | 5.9114 | 3.39E-09 |
| 6 | 142288479 | 145319810 | 2010 | 0.00030672 | 2.71E-09 | 5.21E-05 | 5.8903 | 3.85E-09 |
| 8 | 116096495 | 119685457 | 2358 | 0.00025496 | 1.88E-09 | 4.34E-05 | 5.8736 | 4.26E-09 |
| 1 | 65041704 | 66939404 | 1242 | 0.00029783 | 2.70E-09 | 5.20E-05 | 5.7266 | 1.02E-08 |
| 16 | 63691589 | 65938566 | 1602 | 0.00024526 | 1.84E-09 | 4.29E-05 | 5.7195 | 1.07E-08 |
| 3 | 106982811 | 109522395 | 1621 | 0.00028545 | 2.51E-09 | 5.01E-05 | 5.7016 | 1.19E-08 |
| 2 | 81620841 | 85140680 | 2237 | 0.00025627 | 2.03E-09 | 4.50E-05 | 5.6898 | 1.27E-08 |
| 3 | 49316972 | 51832015 | 625 | 0.00038341 | 4.55E-09 | 6.75E-05 | 5.6828 | 1.32E-08 |
| 10 | 104380410 | 106695048 | 1308 | 0.00034553 | 3.79E-09 | 6.16E-05 | 5.6132 | 1.99E-08 |
| 14 | 98341162 | 99138532 | 801 | 0.00023204 | 1.72E-09 | 4.15E-05 | 5.5927 | 2.24E-08 |
| 1 | 74326907 | 76728135 | 1562 | 0.00030166 | 2.92E-09 | 5.40E-05 | 5.5823 | 2.37E-08 |
| 10 | 19716878 | 22772283 | 1898 | 0.00030586 | 3.03E-09 | 5.50E-05 | 5.5575 | 2.74E-08 |
| 3 | 47727212 | 49316972 | 320 | 0.00032712 | 3.48E-09 | 5.90E-05 | 5.5466 | 2.91E-08 |
| 7 | 78093859 | 79888069 | 1324 | 0.00020573 | 1.41E-09 | 3.76E-05 | 5.4768 | 4.33E-08 |
| 21 | 38711704 | 40482902 | 1378 | 0.0002264 | 1.73E-09 | 4.15E-05 | 5.4511 | 5.01E-08 |
| 10 | 134494673 | 135523865 | 364 | 0.00021525 | 1.56E-09 | 3.95E-05 | 5.4506 | 5.02E-08 |
| 14 | 103012102 | 105001723 | 810 | 0.00028692 | 2.79E-09 | 5.28E-05 | 5.4335 | 5.53E-08 |
| 14 | 32383265 | 34846251 | 1896 | 0.0002012 | 1.40E-09 | 3.74E-05 | 5.3865 | 7.19E-08 |
| 16 | 2764829 | 4001196 | 397 | 0.00016944 | 1.00E-09 | 3.16E-05 | 5.3596 | 8.34E-08 |
| 16 | 71054028 | 72935150 | 689 | 0.00021174 | 1.56E-09 | 3.95E-05 | 5.357 | 8.46E-08 |
| 11 | 133000046 | 134205993 | 809 | 0.00024106 | 2.05E-09 | 4.52E-05 | 5.3288 | 9.89E-08 |
| 21 | 34376999 | 35225465 | 439 | 0.0001863 | 1.22E-09 | 3.50E-05 | 5.3273 | 9.97E-08 |
| 6 | 127540461 | 129861031 | 1392 | 0.00028008 | 2.82E-09 | 5.31E-05 | 5.271 | 1.36E-07 |
| 18 | 26875587 | 27866478 | 666 | 0.00020565 | 1.53E-09 | 3.91E-05 | 5.2611 | 1.43E-07 |
| 1 | 111741737 | 113273306 | 1230 | 0.00023967 | 2.13E-09 | 4.61E-05 | 5.1944 | 2.05E-07 |
| 2 | 159577082 | 161769733 | 1248 | 0.00022317 | 1.85E-09 | 4.30E-05 | 5.194 | 2.06E-07 |
| 10 | 63341695 | 65794114 | 1697 | 0.00025568 | 2.45E-09 | 4.95E-05 | 5.1672 | 2.38E-07 |
| 17 | 29786491 | 31538425 | 874 | 0.0002102 | 1.67E-09 | 4.08E-05 | 5.1497 | 2.61E-07 |
| 11 | 27020461 | 28481593 | 735 | 0.00024176 | 2.22E-09 | 4.71E-05 | 5.128 | 2.93E-07 |
| 9 | 117921960 | 121321537 | 2865 | 0.00026864 | 2.75E-09 | 5.25E-05 | 5.1208 | 3.04E-07 |
| 8 | 104988752 | 107410754 | 1700 | 0.00021589 | 1.79E-09 | 4.23E-05 | 5.0997 | 3.40E-07 |
| 12 | 67909729 | 69826542 | 1339 | 0.00018437 | 1.32E-09 | 3.63E-05 | 5.0801 | 3.77E-07 |
| 2 | 103588215 | 105125034 | 756 | 0.00025932 | 2.61E-09 | 5.11E-05 | 5.0729 | 3.92E-07 |
| 5 | 169505664 | 171074292 | 1102 | 0.00024924 | 2.44E-09 | 4.94E-05 | 5.0494 | 4.43E-07 |
| 1 | 165191702 | 166460517 | 936 | 0.00016233 | 1.04E-09 | 3.22E-05 | 5.0357 | 4.76E-07 |
| 2 | 161769733 | 163503551 | 666 | 0.00025832 | 2.64E-09 | 5.13E-05 | 5.0317 | 4.86E-07 |
| 12 | 83502666 | 84303844 | 606 | 0.00019351 | 1.49E-09 | 3.85E-05 | 5.0209 | 5.14E-07 |
| 20 | 25344231 | 31614823 | 1021 | 0.00023911 | 2.27E-09 | 4.77E-05 | 5.0147 | 5.31E-07 |
| 3 | 25461558 | 26877769 | 889 | 0.00022265 | 1.97E-09 | 4.44E-05 | 5.0131 | 5.36E-07 |
| 16 | 24136875 | 25986862 | 1230 | 0.00025534 | 2.61E-09 | 5.11E-05 | 4.9936 | 5.93E-07 |
| 3 | 20091348 | 21643707 | 1246 | 0.00022292 | 2.01E-09 | 4.49E-05 | 4.9692 | 6.72E-07 |
| 7 | 31137289 | 33555768 | 1879 | 0.00026752 | 2.91E-09 | 5.40E-05 | 4.9563 | 7.19E-07 |
| 14 | 78561945 | 80186579 | 1135 | 0.00022584 | 2.08E-09 | 4.56E-05 | 4.9554 | 7.22E-07 |
| 22 | 44995308 | 46470495 | 1008 | 0.00016731 | 1.15E-09 | 3.39E-05 | 4.9338 | 8.06E-07 |
| 3 | 51832015 | 54081390 | 973 | 0.00024218 | 2.42E-09 | 4.92E-05 | 4.9196 | 8.67E-07 |
| 1 | 96150893 | 97885249 | 1053 | 0.00022744 | 2.14E-09 | 4.63E-05 | 4.9141 | 8.92E-07 |
| 1 | 235819436 | 237555628 | 1223 | 0.00018006 | 1.36E-09 | 3.69E-05 | 4.8854 | 1.03E-06 |
| 7 | 4573428 | 5416232 | 283 | 0.00015979 | 1.07E-09 | 3.28E-05 | 4.8778 | 1.07E-06 |
| 1 | 241582220 | 242071602 | 337 | 0.00019625 | 1.64E-09 | 4.05E-05 | 4.8469 | 1.25E-06 |
| 3 | 88298373 | 94255316 | 1143 | 0.00018134 | 1.41E-09 | 3.76E-05 | 4.8229 | 1.42E-06 |
| 1 | 212842330 | 214578266 | 980 | 0.00020922 | 1.88E-09 | 4.34E-05 | 4.8196 | 1.44E-06 |
| 18 | 37684367 | 39892648 | 1687 | 0.00022401 | 2.18E-09 | 4.67E-05 | 4.8004 | 1.58E-06 |
| 13 | 96087558 | 97519210 | 715 | 0.00020324 | 1.80E-09 | 4.25E-05 | 4.7867 | 1.70E-06 |
| 1 | 201589975 | 203334734 | 949 | 0.00021333 | 2.02E-09 | 4.50E-05 | 4.7432 | 2.10E-06 |
| 17 | 1928731 | 3702312 | 985 | 0.0001967 | 1.75E-09 | 4.18E-05 | 4.7087 | 2.49E-06 |
| 5 | 58524622 | 60935907 | 1299 | 0.00021979 | 2.18E-09 | 4.67E-05 | 4.704 | 2.55E-06 |
| 7 | 98715474 | 100196651 | 358 | 0.00017171 | 1.36E-09 | 3.69E-05 | 4.6597 | 3.17E-06 |
| 16 | 68841363 | 71054028 | 552 | 0.00020538 | 1.97E-09 | 4.44E-05 | 4.6247 | 3.75E-06 |
| 11 | 28481593 | 30141357 | 909 | 0.00020248 | 1.93E-09 | 4.39E-05 | 4.6115 | 4.00E-06 |
| 17 | 64800430 | 67858770 | 1542 | 0.00018678 | 1.65E-09 | 4.06E-05 | 4.6041 | 4.14E-06 |
| 11 | 92077144 | 93276157 | 764 | 0.00019361 | 1.77E-09 | 4.21E-05 | 4.5996 | 4.23E-06 |
| 11 | 72286017 | 74412351 | 926 | 0.00019291 | 1.76E-09 | 4.20E-05 | 4.5952 | 4.32E-06 |
| 6 | 26791233 | 28017819 | 509 | 0.00024125 | 2.76E-09 | 5.25E-05 | 4.5932 | 4.37E-06 |
| 6 | 67329215 | 68849483 | 1287 | 0.00023346 | 2.59E-09 | 5.09E-05 | 4.5899 | 4.44E-06 |
| 10 | 106695048 | 108726686 | 1516 | 0.00022021 | 2.30E-09 | 4.80E-05 | 4.5892 | 4.45E-06 |
| 9 | 26111757 | 28224283 | 1794 | 0.00016369 | 1.28E-09 | 3.58E-05 | 4.57 | 4.88E-06 |
| 13 | 100574095 | 102300523 | 1369 | 0.00020126 | 1.94E-09 | 4.41E-05 | 4.5646 | 5.01E-06 |
| 16 | 60054 | 1207206 | 297 | 0.00022243 | 2.38E-09 | 4.87E-05 | 4.5639 | 5.02E-06 |
| 2 | 206735704 | 208645399 | 1072 | 0.00017794 | 1.52E-09 | 3.90E-05 | 4.558 | 5.16E-06 |
| 12 | 89682122 | 92066155 | 1402 | 0.00020154 | 1.96E-09 | 4.42E-05 | 4.5573 | 5.18E-06 |
| 15 | 53069096 | 54508528 | 1288 | 0.000189 | 1.72E-09 | 4.15E-05 | 4.5523 | 5.31E-06 |
| 2 | 123302906 | 124732250 | 1104 | 0.00017229 | 1.44E-09 | 3.80E-05 | 4.5331 | 5.81E-06 |
| 2 | 118367466 | 121303783 | 1836 | 0.00015403 | 1.16E-09 | 3.41E-05 | 4.5161 | 6.30E-06 |
| 12 | 53039004 | 54778823 | 909 | 0.00016894 | 1.40E-09 | 3.74E-05 | 4.5153 | 6.32E-06 |
| 9 | 121321537 | 122260297 | 736 | 0.00021703 | 2.31E-09 | 4.81E-05 | 4.5147 | 6.34E-06 |
| 4 | 27965868 | 29762208 | 1306 | 0.00021826 | 2.34E-09 | 4.84E-05 | 4.5126 | 6.40E-06 |
| 4 | 10699152 | 12323034 | 1292 | 0.00016937 | 1.42E-09 | 3.77E-05 | 4.4966 | 6.90E-06 |
| 8 | 69863057 | 70867979 | 827 | 0.00017144 | 1.46E-09 | 3.81E-05 | 4.4939 | 6.99E-06 |
| 1 | 173097907 | 175089768 | 677 | 0.00019544 | 1.89E-09 | 4.35E-05 | 4.4898 | 7.13E-06 |
| 16 | 29036613 | 31382943 | 372 | 0.00017775 | 1.58E-09 | 3.97E-05 | 4.4758 | 7.61E-06 |
| 12 | 106958748 | 109025901 | 1275 | 0.00021283 | 2.26E-09 | 4.76E-05 | 4.474 | 7.68E-06 |
| 1 | 48978188 | 49894177 | 431 | 0.00017051 | 1.47E-09 | 3.83E-05 | 4.4501 | 8.58E-06 |
| 8 | 28162392 | 29327896 | 609 | 0.0001626 | 1.34E-09 | 3.66E-05 | 4.4462 | 8.74E-06 |
| 18 | 36365490 | 37684367 | 759 | 0.00021957 | 2.46E-09 | 4.96E-05 | 4.4266 | 9.57E-06 |
| 2 | 199311125 | 201576284 | 1137 | 0.0002199 | 2.47E-09 | 4.97E-05 | 4.4258 | 9.61E-06 |
| 9 | 16659655 | 18661051 | 1867 | 0.00017484 | 1.59E-09 | 3.99E-05 | 4.3873 | 1.15E-05 |
| 4 | 94233299 | 96182188 | 1182 | 0.00019776 | 2.04E-09 | 4.52E-05 | 4.3795 | 1.19E-05 |
| 7 | 140235210 | 141226557 | 484 | 0.00014874 | 1.15E-09 | 3.40E-05 | 4.3802 | 1.19E-05 |
| 20 | 42680176 | 44839056 | 1278 | 0.0001878 | 1.85E-09 | 4.30E-05 | 4.3663 | 1.26E-05 |
| 15 | 94924330 | 96141596 | 965 | 0.00016247 | 1.39E-09 | 3.73E-05 | 4.3611 | 1.29E-05 |
| 20 | 41615460 | 42680176 | 636 | 0.00018422 | 1.80E-09 | 4.24E-05 | 4.3405 | 1.42E-05 |
| 5 | 41888710 | 43983499 | 878 | 0.00018332 | 1.80E-09 | 4.24E-05 | 4.3259 | 1.52E-05 |
| 10 | 67549615 | 69900148 | 1910 | 0.00019313 | 1.99E-09 | 4.46E-05 | 4.3256 | 1.52E-05 |
| 14 | 77229472 | 78561945 | 1005 | 0.00017171 | 1.58E-09 | 3.97E-05 | 4.3236 | 1.54E-05 |
| 11 | 93276157 | 94242260 | 522 | 0.00013633 | 9.99E-10 | 3.16E-05 | 4.313 | 1.61E-05 |
| 13 | 51591091 | 53339622 | 633 | 0.00015538 | 1.30E-09 | 3.60E-05 | 4.3126 | 1.61E-05 |
| 11 | 39382003 | 42310003 | 2094 | 0.0001909 | 1.97E-09 | 4.44E-05 | 4.3007 | 1.70E-05 |
| 16 | 1207206 | 2764829 | 518 | 0.00014892 | 1.20E-09 | 3.46E-05 | 4.3012 | 1.70E-05 |
| 9 | 7154923 | 8456299 | 1650 | 0.00018914 | 1.94E-09 | 4.41E-05 | 4.29 | 1.79E-05 |
| 9 | 10879253 | 12276489 | 1327 | 0.00019661 | 2.10E-09 | 4.59E-05 | 4.2854 | 1.82E-05 |
| 12 | 72645075 | 73818454 | 678 | 0.00014698 | 1.18E-09 | 3.44E-05 | 4.2755 | 1.91E-05 |
| 19 | 34262952 | 36469295 | 1022 | 0.00015176 | 1.26E-09 | 3.55E-05 | 4.2733 | 1.93E-05 |
| 9 | 126971887 | 129059665 | 880 | 0.00021953 | 2.65E-09 | 5.15E-05 | 4.2667 | 1.98E-05 |
| 1 | 219590571 | 221858231 | 1404 | 0.00014263 | 1.12E-09 | 3.35E-05 | 4.259 | 2.05E-05 |
| 11 | 119215476 | 120766806 | 983 | 0.00018904 | 1.98E-09 | 4.45E-05 | 4.2445 | 2.19E-05 |
| 19 | 3019660 | 4348967 | 417 | 0.00015213 | 1.29E-09 | 3.60E-05 | 4.2296 | 2.34E-05 |
| 1 | 49894177 | 51713726 | 464 | 0.00014493 | 1.19E-09 | 3.45E-05 | 4.2032 | 2.63E-05 |
| 7 | 132805848 | 134307596 | 949 | 0.00017838 | 1.81E-09 | 4.25E-05 | 4.1955 | 2.72E-05 |
| 7 | 2772227 | 4573428 | 1551 | 0.00021035 | 2.52E-09 | 5.01E-05 | 4.1945 | 2.74E-05 |

1. SI: Smoking initiation; GERD: Gastroesophageal reflux disease.

2. K is the number of eigenvectors used in the truncated singular value decomposition (SVD), local_rhog represents local genetic covariance estimates, and var stands for variance estimates.

# Table S3: Local genetic correlation between NS and GERD

| **chr** | **start** | **end** | **num_snp** | **local_rhog** | **var** | **se** | **z** | **p** |
| --- | --- | --- | --- | --- | --- | --- | --- | --- |
| 2 | 58297315 | 60292000 | 1301 | -0.00067403 | 4.68E-09 | 6.84E-05 | -9.8515 | 6.75E-23 |
| 9 | 81079055 | 82590928 | 1263 | -0.00055188 | 4.87E-09 | 6.98E-05 | -7.907 | 2.64E-15 |
| 6 | 25684587 | 26791233 | 891 | -0.0005916 | 6.46E-09 | 8.04E-05 | -7.3616 | 1.82E-13 |
| 14 | 78561945 | 80186579 | 1193 | -0.00041534 | 3.52E-09 | 5.93E-05 | -7.001 | 2.54E-12 |
| 1 | 71684405 | 74326907 | 1453 | -0.00050968 | 5.78E-09 | 7.60E-05 | -6.7023 | 2.05E-11 |
| 2 | 21050490 | 23341383 | 1333 | -0.00046735 | 4.93E-09 | 7.02E-05 | -6.6594 | 2.75E-11 |
| 2 | 50818928 | 52491133 | 1618 | -0.00043754 | 4.49E-09 | 6.70E-05 | -6.5271 | 6.70E-11 |
| 2 | 173138905 | 175588929 | 1574 | -0.00038844 | 3.56E-09 | 5.97E-05 | -6.5087 | 7.58E-11 |
| 1 | 43758457 | 44969183 | 615 | -0.00042256 | 4.25E-09 | 6.52E-05 | -6.4781 | 9.29E-11 |
| 11 | 112459488 | 114257728 | 1463 | -0.0005611 | 7.88E-09 | 8.88E-05 | -6.3212 | 2.60E-10 |
| 1 | 96150893 | 97885249 | 1116 | -0.00038295 | 3.72E-09 | 6.10E-05 | -6.2783 | 3.42E-10 |
| 7 | 2772227 | 4573428 | 1662 | -0.00042532 | 4.61E-09 | 6.79E-05 | -6.2651 | 3.73E-10 |
| 9 | 126971887 | 129059665 | 926 | -0.00042768 | 4.84E-09 | 6.96E-05 | -6.1464 | 7.93E-10 |
| 22 | 44995308 | 46470495 | 1089 | -0.0002691 | 1.93E-09 | 4.39E-05 | -6.1271 | 8.95E-10 |
| 7 | 116780178 | 118351581 | 893 | -0.00033484 | 3.02E-09 | 5.50E-05 | -6.0932 | 1.11E-09 |
| 11 | 133000046 | 134205993 | 857 | -0.00037393 | 3.84E-09 | 6.20E-05 | -6.0306 | 1.63E-09 |
| 21 | 38711704 | 40482902 | 1468 | -0.00033029 | 3.01E-09 | 5.49E-05 | -6.0207 | 1.74E-09 |
| 1 | 115880593 | 118839067 | 1607 | -0.00032544 | 2.94E-09 | 5.42E-05 | -6.0043 | 1.92E-09 |
| 13 | 100574095 | 102300523 | 1440 | -0.00036184 | 3.65E-09 | 6.05E-05 | -5.9854 | 2.16E-09 |
| 2 | 103588215 | 105125034 | 792 | -0.00041316 | 4.84E-09 | 6.96E-05 | -5.9388 | 2.87E-09 |
| 13 | 65200602 | 67844453 | 1651 | -0.00038868 | 4.32E-09 | 6.57E-05 | -5.9127 | 3.37E-09 |
| 18 | 26875587 | 27866478 | 700 | -0.00029192 | 2.58E-09 | 5.08E-05 | -5.7484 | 9.01E-09 |
| 10 | 104380410 | 106695048 | 1384 | -0.00045995 | 6.48E-09 | 8.05E-05 | -5.7118 | 1.12E-08 |
| 1 | 74326907 | 76728135 | 1647 | -0.00040298 | 5.08E-09 | 7.13E-05 | -5.6512 | 1.59E-08 |
| 14 | 103012102 | 105001723 | 875 | -0.00040012 | 5.02E-09 | 7.08E-05 | -5.6489 | 1.61E-08 |
| 16 | 63691589 | 65938566 | 1681 | -0.00030178 | 2.89E-09 | 5.37E-05 | -5.6174 | 1.94E-08 |
| 1 | 76728135 | 79661198 | 1966 | -0.00032657 | 3.38E-09 | 5.81E-05 | -5.6166 | 1.95E-08 |
| 2 | 181312739 | 182266031 | 696 | -0.00029959 | 2.90E-09 | 5.38E-05 | -5.566 | 2.61E-08 |
| 20 | 25344231 | 31614823 | 1086 | -0.00031943 | 3.34E-09 | 5.77E-05 | -5.5313 | 3.18E-08 |
| 14 | 98341162 | 99138532 | 840 | -0.00028899 | 2.73E-09 | 5.23E-05 | -5.5281 | 3.24E-08 |
| 1 | 219590571 | 221858231 | 1492 | -0.0002693 | 2.37E-09 | 4.87E-05 | -5.5265 | 3.27E-08 |
| 16 | 1207206 | 2764829 | 567 | -0.0002574 | 2.17E-09 | 4.66E-05 | -5.5255 | 3.28E-08 |
| 3 | 51832015 | 54081390 | 1022 | -0.00034545 | 3.94E-09 | 6.27E-05 | -5.5063 | 3.67E-08 |
| 13 | 96087558 | 97519210 | 758 | -0.00032261 | 3.44E-09 | 5.86E-05 | -5.5028 | 3.74E-08 |
| 21 | 34376999 | 35225465 | 464 | -0.00024308 | 2.00E-09 | 4.47E-05 | -5.4338 | 5.52E-08 |
| 18 | 36365490 | 37684367 | 802 | -0.00033379 | 3.84E-09 | 6.19E-05 | -5.3899 | 7.05E-08 |
| 2 | 81620841 | 85140680 | 2376 | -0.00031364 | 3.42E-09 | 5.85E-05 | -5.3656 | 8.07E-08 |
| 8 | 59068651 | 59728100 | 620 | -0.00021961 | 1.68E-09 | 4.10E-05 | -5.3543 | 8.59E-08 |
| 5 | 159973944 | 161482133 | 1099 | -0.00030118 | 3.17E-09 | 5.63E-05 | -5.3472 | 8.93E-08 |
| 8 | 90638162 | 93554386 | 1517 | -0.00036662 | 4.71E-09 | 6.86E-05 | -5.3436 | 9.11E-08 |
| 3 | 20091348 | 21643707 | 1317 | -0.00032207 | 3.65E-09 | 6.04E-05 | -5.3317 | 9.73E-08 |
| 4 | 163243679 | 164478234 | 731 | -0.00030648 | 3.31E-09 | 5.75E-05 | -5.3302 | 9.81E-08 |
| 12 | 23820634 | 25371083 | 1406 | -0.00027116 | 2.65E-09 | 5.15E-05 | -5.2703 | 1.36E-07 |
| 14 | 32383265 | 34846251 | 2020 | -0.00024934 | 2.26E-09 | 4.75E-05 | -5.2471 | 1.55E-07 |
| 10 | 134494673 | 135523865 | 388 | -0.00027554 | 2.83E-09 | 5.32E-05 | -5.179 | 2.23E-07 |
| 1 | 201589975 | 203334734 | 996 | -0.00031797 | 3.78E-09 | 6.15E-05 | -5.1688 | 2.36E-07 |
| 18 | 77149991 | 78017158 | 487 | -0.0003373 | 4.27E-09 | 6.53E-05 | -5.163 | 2.43E-07 |
| 20 | 58406572 | 59561724 | 1015 | -0.00030504 | 3.58E-09 | 5.99E-05 | -5.0955 | 3.48E-07 |
| 20 | 42680176 | 44839056 | 1340 | -0.00028847 | 3.21E-09 | 5.67E-05 | -5.0906 | 3.57E-07 |
| 4 | 139556511 | 141084631 | 800 | -0.00038814 | 5.84E-09 | 7.64E-05 | -5.0808 | 3.76E-07 |
| 1 | 79661198 | 81354191 | 1162 | -0.00032212 | 4.08E-09 | 6.39E-05 | -5.0433 | 4.58E-07 |
| 4 | 118479918 | 119933512 | 1098 | -0.00024144 | 2.30E-09 | 4.80E-05 | -5.0308 | 4.89E-07 |
| 12 | 89682122 | 92066155 | 1485 | -0.00029459 | 3.44E-09 | 5.86E-05 | -5.0263 | 5.00E-07 |
| 4 | 18841874 | 20544557 | 1127 | -0.0003094 | 3.79E-09 | 6.16E-05 | -5.023 | 5.09E-07 |
| 5 | 169505664 | 171074292 | 1163 | -0.00032306 | 4.18E-09 | 6.47E-05 | -4.9959 | 5.86E-07 |
| 8 | 28162392 | 29327896 | 651 | -0.0002479 | 2.50E-09 | 5.00E-05 | -4.962 | 6.98E-07 |
| 2 | 206735704 | 208645399 | 1143 | -0.0002606 | 2.77E-09 | 5.26E-05 | -4.9505 | 7.40E-07 |
| 16 | 17644749 | 18643607 | 331 | -0.00018074 | 1.34E-09 | 3.66E-05 | -4.9359 | 7.98E-07 |
| 11 | 27020461 | 28481593 | 772 | -0.00029592 | 3.62E-09 | 6.02E-05 | -4.9155 | 8.86E-07 |
| 15 | 35083251 | 37456502 | 1771 | -0.00026326 | 2.90E-09 | 5.39E-05 | -4.885 | 1.03E-06 |
| 10 | 63341695 | 65794114 | 1782 | -0.00033658 | 4.75E-09 | 6.89E-05 | -4.8838 | 1.04E-06 |
| 12 | 61123729 | 64032461 | 2348 | -0.00032842 | 4.55E-09 | 6.74E-05 | -4.8709 | 1.11E-06 |
| 10 | 108726686 | 110317705 | 1454 | -0.00027 | 3.08E-09 | 5.55E-05 | -4.8665 | 1.14E-06 |
| 4 | 141084631 | 143443265 | 1436 | -0.00030016 | 3.83E-09 | 6.19E-05 | -4.8474 | 1.25E-06 |
| 17 | 1928731 | 3702312 | 1045 | -0.00028163 | 3.41E-09 | 5.84E-05 | -4.8261 | 1.39E-06 |
| 1 | 226810860 | 229156248 | 1407 | -0.00026004 | 2.92E-09 | 5.40E-05 | -4.8165 | 1.46E-06 |
| 1 | 32438685 | 34799758 | 1125 | -0.00034021 | 5.01E-09 | 7.08E-05 | -4.8071 | 1.53E-06 |
| 11 | 17578402 | 19569535 | 1430 | -0.00028222 | 3.45E-09 | 5.87E-05 | -4.8068 | 1.53E-06 |
| 13 | 53339622 | 54682864 | 1128 | -0.00031914 | 4.43E-09 | 6.66E-05 | -4.7931 | 1.64E-06 |
| 5 | 120452166 | 122556905 | 1413 | -0.00026346 | 3.03E-09 | 5.51E-05 | -4.7842 | 1.72E-06 |
| 18 | 39892648 | 42922106 | 2102 | -0.00029843 | 3.91E-09 | 6.26E-05 | -4.771 | 1.83E-06 |
| 7 | 113712387 | 116780178 | 1545 | -0.0003432 | 5.20E-09 | 7.21E-05 | -4.7575 | 1.96E-06 |
| 2 | 137042794 | 138698117 | 1195 | -0.00027856 | 3.44E-09 | 5.86E-05 | -4.7501 | 2.03E-06 |
| 12 | 119754110 | 122007651 | 1107 | -0.00030341 | 4.14E-09 | 6.44E-05 | -4.7146 | 2.42E-06 |
| 2 | 142518602 | 144519484 | 1464 | -0.0003118 | 4.38E-09 | 6.62E-05 | -4.7111 | 2.46E-06 |
| 10 | 10249396 | 12586797 | 1929 | -0.00025378 | 2.90E-09 | 5.39E-05 | -4.7088 | 2.49E-06 |
| 1 | 235819436 | 237555628 | 1318 | -0.0002349 | 2.53E-09 | 5.03E-05 | -4.6741 | 2.95E-06 |
| 15 | 98030910 | 99244059 | 1011 | -0.00022944 | 2.41E-09 | 4.91E-05 | -4.6731 | 2.97E-06 |
| 16 | 68841363 | 71054028 | 579 | -0.00028211 | 3.66E-09 | 6.05E-05 | -4.6645 | 3.09E-06 |
| 1 | 165191702 | 166460517 | 980 | -0.00020297 | 1.90E-09 | 4.36E-05 | -4.6542 | 3.25E-06 |
| 11 | 28481593 | 30141357 | 977 | -0.0002603 | 3.16E-09 | 5.62E-05 | -4.6308 | 3.64E-06 |
| 4 | 122657987 | 124286481 | 857 | -0.00020263 | 1.92E-09 | 4.38E-05 | -4.6265 | 3.72E-06 |
| 8 | 116096495 | 119685457 | 2504 | -0.0002606 | 3.19E-09 | 5.65E-05 | -4.6135 | 3.96E-06 |
| 21 | 31197025 | 32668642 | 1063 | -0.00021961 | 2.27E-09 | 4.76E-05 | -4.6131 | 3.97E-06 |
| 2 | 10133 | 1781022 | 1310 | -0.0002822 | 3.75E-09 | 6.12E-05 | -4.6109 | 4.01E-06 |
| 21 | 35225465 | 36524373 | 974 | -0.00023565 | 2.63E-09 | 5.13E-05 | -4.5919 | 4.39E-06 |
| 16 | 60054 | 1207206 | 327 | -0.00029882 | 4.28E-09 | 6.54E-05 | -4.5686 | 4.91E-06 |
| 16 | 2764829 | 4001196 | 429 | -0.00019593 | 1.85E-09 | 4.30E-05 | -4.558 | 5.16E-06 |
| 1 | 216243634 | 218705513 | 2222 | -0.00023048 | 2.58E-09 | 5.07E-05 | -4.5415 | 5.59E-06 |
| 9 | 134127888 | 135298842 | 703 | -0.00029433 | 4.20E-09 | 6.48E-05 | -4.5408 | 5.61E-06 |
| 9 | 10879253 | 12276489 | 1405 | -0.00026679 | 3.49E-09 | 5.91E-05 | -4.5164 | 6.29E-06 |
| 11 | 72286017 | 74412351 | 987 | -0.00024144 | 2.86E-09 | 5.35E-05 | -4.511 | 6.45E-06 |
| 4 | 27343722 | 27965868 | 465 | -0.00021295 | 2.28E-09 | 4.77E-05 | -4.4628 | 8.09E-06 |
| 3 | 25461558 | 26877769 | 948 | -0.00025911 | 3.39E-09 | 5.83E-05 | -4.4477 | 8.68E-06 |
| 1 | 90066303 | 91886317 | 1054 | -0.00027933 | 3.96E-09 | 6.30E-05 | -4.4364 | 9.15E-06 |
| 4 | 136566407 | 138541971 | 1260 | -0.0002463 | 3.08E-09 | 5.55E-05 | -4.4344 | 9.23E-06 |
| 7 | 53176312 | 54222976 | 896 | -0.00022077 | 2.49E-09 | 4.99E-05 | -4.4207 | 9.84E-06 |
| 3 | 106982811 | 109522395 | 1702 | -0.00028692 | 4.23E-09 | 6.50E-05 | -4.4111 | 1.03E-05 |
| 14 | 77229472 | 78561945 | 1065 | -0.00023309 | 2.80E-09 | 5.29E-05 | -4.404 | 1.06E-05 |
| 7 | 157634597 | 159128575 | 841 | -0.00026286 | 3.56E-09 | 5.97E-05 | -4.4027 | 1.07E-05 |
| 5 | 41888710 | 43983499 | 927 | -0.00023796 | 2.92E-09 | 5.41E-05 | -4.4002 | 1.08E-05 |
| 12 | 67909729 | 69826542 | 1413 | -0.00021209 | 2.33E-09 | 4.83E-05 | -4.3938 | 1.11E-05 |
| 10 | 33707968 | 35109355 | 1021 | -0.00023168 | 2.79E-09 | 5.28E-05 | -4.3854 | 1.16E-05 |
| 6 | 26791233 | 28017819 | 539 | -0.00030124 | 4.79E-09 | 6.92E-05 | -4.3539 | 1.34E-05 |
| 1 | 111741737 | 113273306 | 1290 | -0.00026651 | 3.76E-09 | 6.14E-05 | -4.3434 | 1.40E-05 |
| 9 | 85440801 | 86938196 | 1271 | -0.00026677 | 3.78E-09 | 6.15E-05 | -4.3398 | 1.43E-05 |
| 20 | 18972203 | 20959710 | 1718 | -0.00020873 | 2.31E-09 | 4.81E-05 | -4.3382 | 1.44E-05 |
| 5 | 136376050 | 139265072 | 1207 | -0.00027213 | 3.94E-09 | 6.28E-05 | -4.3343 | 1.46E-05 |
| 8 | 59728100 | 62329099 | 1763 | -0.00027488 | 4.07E-09 | 6.38E-05 | -4.3085 | 1.64E-05 |
| 7 | 98715474 | 100196651 | 386 | -0.00020734 | 2.33E-09 | 4.83E-05 | -4.2937 | 1.76E-05 |
| 3 | 47727212 | 49316972 | 351 | -0.00031428 | 5.39E-09 | 7.34E-05 | -4.2825 | 1.85E-05 |
| 7 | 25909555 | 28360309 | 1945 | -0.00019979 | 2.18E-09 | 4.67E-05 | -4.2802 | 1.87E-05 |
| 2 | 123302906 | 124732250 | 1167 | -0.00022293 | 2.72E-09 | 5.22E-05 | -4.2709 | 1.95E-05 |
| 3 | 88298373 | 94255316 | 1215 | -0.00022286 | 2.74E-09 | 5.23E-05 | -4.2587 | 2.06E-05 |
| 3 | 49316972 | 51832015 | 661 | -0.00036952 | 7.54E-09 | 8.69E-05 | -4.2547 | 2.09E-05 |
| 2 | 159577082 | 161769733 | 1324 | -0.00026254 | 3.81E-09 | 6.17E-05 | -4.2527 | 2.11E-05 |
| 16 | 71054028 | 72935150 | 743 | -0.00022788 | 2.91E-09 | 5.40E-05 | -4.2232 | 2.41E-05 |
| 7 | 22507629 | 23471442 | 569 | -0.00019045 | 2.04E-09 | 4.52E-05 | -4.2156 | 2.49E-05 |
| 2 | 78079264 | 80050403 | 1962 | -0.0002472 | 3.45E-09 | 5.88E-05 | -4.2062 | 2.60E-05 |
| 1 | 173097907 | 175089768 | 719 | -0.00023827 | 3.22E-09 | 5.68E-05 | -4.1984 | 2.69E-05 |
| 10 | 97822357 | 100241302 | 1489 | -0.00022629 | 2.92E-09 | 5.41E-05 | -4.1854 | 2.85E-05 |
| 4 | 132954033 | 134628434 | 1118 | -0.00022552 | 2.91E-09 | 5.40E-05 | -4.1775 | 2.95E-05 |

1. NS: Never smoking; GERD: Gastroesophageal reflux disease.

2. K is the number of eigenvectors used in the truncated singular value decomposition (SVD), local_rhog represents local genetic covariance estimates, and var stands for variance estimates.

# Table S4: Local genetic correlation between ES and GERD

| **chr** | **start** | **end** | **num_snp** | **local_rhog** | **var** | **se** | **z** | **p** |
| --- | --- | --- | --- | --- | --- | --- | --- | --- |
| 2 | 58297315 | 60292000 | 1301 | 0.00057492 | 3.47E-09 | 5.89E-05 | 9.7558 | 1.74E-22 |
| 18 | 39892648 | 42922106 | 2102 | 0.00041628 | 3.17E-09 | 5.63E-05 | 7.3885 | 1.48E-13 |
| 22 | 44995308 | 46470495 | 1089 | 0.00028153 | 1.59E-09 | 3.99E-05 | 7.0586 | 1.68E-12 |
| 6 | 127540461 | 129861031 | 1480 | 0.00037389 | 3.16E-09 | 5.62E-05 | 6.6521 | 2.89E-11 |
| 7 | 116780178 | 118351581 | 893 | 0.00031173 | 2.42E-09 | 4.92E-05 | 6.3305 | 2.44E-10 |
| 1 | 96150893 | 97885249 | 1116 | 0.00030996 | 2.57E-09 | 5.07E-05 | 6.1116 | 9.86E-10 |
| 9 | 81079055 | 82590928 | 1263 | 0.00036169 | 3.70E-09 | 6.08E-05 | 5.9443 | 2.78E-09 |
| 6 | 110304247 | 112345014 | 1156 | 0.00033555 | 3.29E-09 | 5.74E-05 | 5.8473 | 5.00E-09 |
| 13 | 100574095 | 102300523 | 1440 | 0.00030309 | 2.69E-09 | 5.19E-05 | 5.8393 | 5.24E-09 |
| 1 | 71684405 | 74326907 | 1453 | 0.00037582 | 4.24E-09 | 6.51E-05 | 5.7748 | 7.71E-09 |
| 15 | 67094767 | 69017999 | 1223 | 0.00027279 | 2.25E-09 | 4.75E-05 | 5.7476 | 9.05E-09 |
| 16 | 17644749 | 18643607 | 331 | 0.00018368 | 1.05E-09 | 3.24E-05 | 5.6628 | 1.49E-08 |
| 1 | 74326907 | 76728135 | 1647 | 0.00033754 | 3.69E-09 | 6.08E-05 | 5.5532 | 2.81E-08 |
| 11 | 133000046 | 134205993 | 857 | 0.00028915 | 2.75E-09 | 5.24E-05 | 5.5164 | 3.46E-08 |
| 18 | 26875587 | 27866478 | 700 | 0.00023144 | 1.78E-09 | 4.22E-05 | 5.4887 | 4.05E-08 |
| 4 | 139556511 | 141084631 | 800 | 0.00035169 | 4.11E-09 | 6.41E-05 | 5.4862 | 4.11E-08 |
| 11 | 24088517 | 26045753 | 1720 | 0.00029447 | 2.92E-09 | 5.41E-05 | 5.4457 | 5.16E-08 |
| 12 | 110336719 | 113263518 | 730 | 0.00025975 | 2.29E-09 | 4.78E-05 | 5.4302 | 5.63E-08 |
| 2 | 50818928 | 52491133 | 1618 | 0.00030205 | 3.10E-09 | 5.56E-05 | 5.4281 | 5.69E-08 |
| 2 | 21050490 | 23341383 | 1333 | 0.00032431 | 3.63E-09 | 6.03E-05 | 5.3794 | 7.48E-08 |
| 5 | 117346213 | 118608820 | 805 | 0.00020283 | 1.43E-09 | 3.78E-05 | 5.3647 | 8.11E-08 |
| 1 | 115880593 | 118839067 | 1607 | 0.00025439 | 2.25E-09 | 4.75E-05 | 5.359 | 8.37E-08 |
| 11 | 27020461 | 28481593 | 772 | 0.00028121 | 2.76E-09 | 5.25E-05 | 5.3559 | 8.51E-08 |
| 13 | 96087558 | 97519210 | 758 | 0.00025228 | 2.22E-09 | 4.71E-05 | 5.3528 | 8.66E-08 |
| 2 | 173138905 | 175588929 | 1574 | 0.00025131 | 2.23E-09 | 4.73E-05 | 5.3178 | 1.05E-07 |
| 2 | 103588215 | 105125034 | 792 | 0.0002915 | 3.21E-09 | 5.67E-05 | 5.145 | 2.68E-07 |
| 3 | 51832015 | 54081390 | 1022 | 0.00026832 | 2.76E-09 | 5.25E-05 | 5.1062 | 3.29E-07 |
| 19 | 11284028 | 13471127 | 687 | 0.00021269 | 1.74E-09 | 4.17E-05 | 5.1037 | 3.33E-07 |
| 2 | 78079264 | 80050403 | 1962 | 0.0002584 | 2.63E-09 | 5.13E-05 | 5.0381 | 4.70E-07 |
| 3 | 153256571 | 154714218 | 959 | 0.00023809 | 2.24E-09 | 4.74E-05 | 5.0275 | 4.97E-07 |
| 20 | 18972203 | 20959710 | 1718 | 0.00020047 | 1.60E-09 | 4.00E-05 | 5.0087 | 5.48E-07 |
| 11 | 112459488 | 114257728 | 1463 | 0.00039181 | 6.16E-09 | 7.85E-05 | 4.9903 | 6.03E-07 |
| 4 | 18841874 | 20544557 | 1127 | 0.00027386 | 3.02E-09 | 5.50E-05 | 4.9805 | 6.34E-07 |
| 7 | 124167552 | 126519170 | 1855 | 0.00028517 | 3.32E-09 | 5.76E-05 | 4.9513 | 7.37E-07 |
| 14 | 78561945 | 80186579 | 1193 | 0.00024294 | 2.42E-09 | 4.92E-05 | 4.9376 | 7.91E-07 |
| 7 | 113712387 | 116780178 | 1545 | 0.00030526 | 3.83E-09 | 6.19E-05 | 4.9347 | 8.03E-07 |
| 5 | 169505664 | 171074292 | 1163 | 0.00028137 | 3.26E-09 | 5.71E-05 | 4.9305 | 8.20E-07 |
| 2 | 81620841 | 85140680 | 2376 | 0.00023812 | 2.34E-09 | 4.84E-05 | 4.9235 | 8.50E-07 |
| 13 | 65200602 | 67844453 | 1651 | 0.00026871 | 3.02E-09 | 5.50E-05 | 4.8865 | 1.03E-06 |
| 15 | 40384132 | 41177514 | 380 | 0.00023403 | 2.31E-09 | 4.80E-05 | 4.8736 | 1.10E-06 |
| 21 | 31197025 | 32668642 | 1063 | 0.00018192 | 1.40E-09 | 3.74E-05 | 4.8636 | 1.15E-06 |
| 16 | 71054028 | 72935150 | 743 | 0.0002404 | 2.46E-09 | 4.96E-05 | 4.8455 | 1.26E-06 |
| 16 | 49007926 | 52035823 | 1892 | 0.00020711 | 1.85E-09 | 4.31E-05 | 4.8093 | 1.51E-06 |
| 6 | 100630146 | 102636772 | 1334 | 0.00023614 | 2.41E-09 | 4.91E-05 | 4.8086 | 1.52E-06 |
| 16 | 2764829 | 4001196 | 429 | 0.00016866 | 1.25E-09 | 3.53E-05 | 4.7767 | 1.78E-06 |
| 9 | 10879253 | 12276489 | 1405 | 0.0002424 | 2.59E-09 | 5.09E-05 | 4.7588 | 1.95E-06 |
| 9 | 85440801 | 86938196 | 1271 | 0.00024489 | 2.68E-09 | 5.18E-05 | 4.7302 | 2.24E-06 |
| 16 | 63691589 | 65938566 | 1681 | 0.00021215 | 2.02E-09 | 4.50E-05 | 4.7175 | 2.39E-06 |
| 20 | 58406572 | 59561724 | 1015 | 0.00022884 | 2.45E-09 | 4.95E-05 | 4.6243 | 3.76E-06 |
| 10 | 116421406 | 119523934 | 1973 | 0.00020258 | 1.94E-09 | 4.40E-05 | 4.6039 | 4.15E-06 |
| 11 | 32276901 | 33958739 | 1011 | 0.00018354 | 1.61E-09 | 4.01E-05 | 4.5807 | 4.63E-06 |
| 22 | 43714200 | 44995308 | 1216 | 0.00019564 | 1.83E-09 | 4.28E-05 | 4.5677 | 4.93E-06 |
| 1 | 216243634 | 218705513 | 2222 | 0.00018542 | 1.65E-09 | 4.06E-05 | 4.5631 | 5.04E-06 |
| 6 | 25684587 | 26791233 | 891 | 0.00032616 | 5.12E-09 | 7.15E-05 | 4.5604 | 5.11E-06 |
| 1 | 76728135 | 79661198 | 1966 | 0.00021512 | 2.27E-09 | 4.76E-05 | 4.5162 | 6.30E-06 |
| 1 | 165191702 | 166460517 | 980 | 0.00016855 | 1.39E-09 | 3.73E-05 | 4.5142 | 6.35E-06 |
| 7 | 2772227 | 4573428 | 1662 | 0.00025986 | 3.32E-09 | 5.76E-05 | 4.5109 | 6.45E-06 |
| 4 | 45189157 | 47411896 | 1129 | 0.00021406 | 2.27E-09 | 4.77E-05 | 4.4883 | 7.18E-06 |
| 9 | 126971887 | 129059665 | 926 | 0.00025583 | 3.29E-09 | 5.74E-05 | 4.4573 | 8.30E-06 |
| 4 | 122657987 | 124286481 | 857 | 0.00015981 | 1.29E-09 | 3.59E-05 | 4.457 | 8.31E-06 |
| 1 | 154770403 | 156336133 | 454 | 0.00017945 | 1.69E-09 | 4.11E-05 | 4.3634 | 1.28E-05 |
| 18 | 36365490 | 37684367 | 802 | 0.00023655 | 2.97E-09 | 5.45E-05 | 4.3382 | 1.44E-05 |
| 14 | 32383265 | 34846251 | 2020 | 0.00016797 | 1.53E-09 | 3.91E-05 | 4.3005 | 1.70E-05 |
| 12 | 67909729 | 69826542 | 1413 | 0.00016291 | 1.46E-09 | 3.82E-05 | 4.2665 | 1.99E-05 |
| 6 | 153094496 | 154974120 | 1758 | 0.00017843 | 1.75E-09 | 4.19E-05 | 4.2613 | 2.03E-05 |
| 14 | 98341162 | 99138532 | 840 | 0.00018501 | 1.89E-09 | 4.34E-05 | 4.2602 | 2.04E-05 |
| 18 | 77149991 | 78017158 | 487 | 0.00021447 | 2.54E-09 | 5.04E-05 | 4.2597 | 2.05E-05 |
| 1 | 201589975 | 203334734 | 996 | 0.00021355 | 2.52E-09 | 5.02E-05 | 4.2508 | 2.13E-05 |
| 17 | 49898957 | 50719055 | 582 | 0.00021632 | 2.62E-09 | 5.12E-05 | 4.2237 | 2.40E-05 |
| 8 | 90638162 | 93554386 | 1517 | 0.00024562 | 3.39E-09 | 5.82E-05 | 4.2177 | 2.47E-05 |
| 8 | 116096495 | 119685457 | 2504 | 0.00019848 | 2.22E-09 | 4.71E-05 | 4.2164 | 2.48E-05 |
| 15 | 35083251 | 37456502 | 1771 | 0.00019076 | 2.07E-09 | 4.55E-05 | 4.197 | 2.70E-05 |

1. ES: Ever smoking; GERD: Gastroesophageal reflux disease.

2. K is the number of eigenvectors used in the truncated singular value decomposition (SVD), local_rhog represents local genetic covariance estimates, and var stands for variance estimates.

# Table S5: Local genetic correlation between CPD and GERD

| **chr** | **start** | **end** | **num_snp** | **local_rhog** | **var** | **se** | **z** | **p** |
| --- | --- | --- | --- | --- | --- | --- | --- | --- |
| 18 | 39892648 | 42922106 | 1994 | -0.00052066 | 4.24E-09 | 6.51E-05 | -7.9979 | 1.27E-15 |
| 7 | 31137289 | 33555768 | 1879 | -0.00068238 | 7.34E-09 | 8.57E-05 | -7.9644 | 1.66E-15 |
| 1 | 76728135 | 79661198 | 1844 | -0.00045289 | 4.44E-09 | 6.66E-05 | -6.7974 | 1.06E-11 |
| 2 | 58297315 | 60292000 | 1221 | -0.00041803 | 4.38E-09 | 6.62E-05 | -6.3136 | 2.73E-10 |
| 1 | 74326907 | 76728135 | 1562 | -0.00048084 | 5.99E-09 | 7.74E-05 | -6.2148 | 5.14E-10 |
| 3 | 23804865 | 25461558 | 1269 | -0.00039609 | 4.34E-09 | 6.59E-05 | -6.0104 | 1.85E-09 |
| 4 | 2842979 | 3846040 | 416 | -0.00047434 | 6.67E-09 | 8.17E-05 | -5.8085 | 6.30E-09 |
| 4 | 27965868 | 29762208 | 1306 | -0.00036288 | 4.20E-09 | 6.48E-05 | -5.602 | 2.12E-08 |
| 9 | 117921960 | 121321537 | 2866 | -0.0004336 | 6.24E-09 | 7.90E-05 | -5.4907 | 4.00E-08 |
| 22 | 17674295 | 18296088 | 347 | -0.00026898 | 2.42E-09 | 4.92E-05 | -5.4626 | 4.69E-08 |
| 4 | 66600492 | 68059497 | 914 | -0.00041411 | 6.09E-09 | 7.80E-05 | -5.3081 | 1.11E-07 |
| 16 | 68841363 | 71054028 | 552 | -0.0003485 | 4.47E-09 | 6.69E-05 | -5.2106 | 1.88E-07 |
| 9 | 36743283 | 38641599 | 1223 | -0.00030746 | 3.50E-09 | 5.91E-05 | -5.2 | 1.99E-07 |
| 13 | 111232073 | 112247592 | 764 | -0.00035452 | 4.70E-09 | 6.86E-05 | -5.1706 | 2.33E-07 |
| 1 | 88128631 | 90066303 | 913 | -0.00032063 | 3.94E-09 | 6.28E-05 | -5.1093 | 3.23E-07 |
| 8 | 116096495 | 119685457 | 2358 | -0.00032508 | 4.14E-09 | 6.43E-05 | -5.0532 | 4.34E-07 |
| 12 | 73818454 | 76511314 | 1653 | -0.00031932 | 4.03E-09 | 6.35E-05 | -5.0313 | 4.87E-07 |
| 11 | 112459488 | 114257728 | 1392 | -0.00043394 | 7.52E-09 | 8.67E-05 | -5.0053 | 5.58E-07 |
| 6 | 85209989 | 87069500 | 1348 | -0.00023671 | 2.25E-09 | 4.74E-05 | -4.9954 | 5.87E-07 |
| 4 | 103221356 | 105305294 | 1033 | -0.00034543 | 4.79E-09 | 6.92E-05 | -4.9899 | 6.04E-07 |
| 4 | 20544557 | 22319347 | 1286 | -0.00027739 | 3.23E-09 | 5.68E-05 | -4.881 | 1.06E-06 |
| 12 | 89682122 | 92066155 | 1402 | -0.0003341 | 4.69E-09 | 6.85E-05 | -4.8795 | 1.06E-06 |
| 1 | 65041704 | 66939404 | 1242 | -0.0003815 | 6.12E-09 | 7.82E-05 | -4.8761 | 1.08E-06 |
| 6 | 33236497 | 35455756 | 1124 | -0.00039032 | 6.47E-09 | 8.04E-05 | -4.852 | 1.22E-06 |
| 8 | 41721454 | 42773823 | 288 | -0.00036042 | 5.53E-09 | 7.44E-05 | -4.8447 | 1.27E-06 |
| 19 | 20905757 | 22732896 | 805 | -0.00025053 | 2.71E-09 | 5.20E-05 | -4.8138 | 1.48E-06 |
| 1 | 111741737 | 113273306 | 1230 | -0.00030505 | 4.04E-09 | 6.36E-05 | -4.7984 | 1.60E-06 |
| 4 | 134628434 | 136566407 | 1062 | -0.00029902 | 3.91E-09 | 6.25E-05 | -4.7817 | 1.74E-06 |
| 1 | 49894177 | 51713726 | 464 | -0.00025374 | 2.91E-09 | 5.39E-05 | -4.7041 | 2.55E-06 |
| 10 | 134494673 | 135523865 | 364 | -0.00027414 | 3.46E-09 | 5.88E-05 | -4.6613 | 3.14E-06 |
| 1 | 34799758 | 37549183 | 919 | -0.00033985 | 5.39E-09 | 7.34E-05 | -4.6287 | 3.68E-06 |
| 3 | 106982811 | 109522395 | 1621 | -0.00033739 | 5.36E-09 | 7.32E-05 | -4.6105 | 4.02E-06 |
| 4 | 15147446 | 15927009 | 457 | -0.00023994 | 2.71E-09 | 5.21E-05 | -4.6068 | 4.09E-06 |
| 21 | 19480375 | 20962339 | 1149 | -0.00023667 | 2.65E-09 | 5.15E-05 | -4.598 | 4.27E-06 |
| 8 | 22897057 | 24674718 | 1205 | -0.00024454 | 2.84E-09 | 5.33E-05 | -4.5897 | 4.44E-06 |
| 5 | 91840542 | 93809984 | 740 | -0.00027039 | 3.54E-09 | 5.95E-05 | -4.5446 | 5.50E-06 |
| 14 | 29972145 | 32383265 | 1094 | -0.00028462 | 3.99E-09 | 6.31E-05 | -4.5075 | 6.56E-06 |
| 7 | 78093859 | 79888069 | 1324 | -0.00024194 | 2.94E-09 | 5.42E-05 | -4.4598 | 8.20E-06 |
| 2 | 147277162 | 150210292 | 1375 | -0.00028005 | 4.01E-09 | 6.33E-05 | -4.4214 | 9.81E-06 |
| 6 | 78957728 | 80691170 | 1244 | -0.00029984 | 4.69E-09 | 6.85E-05 | -4.3782 | 1.20E-05 |
| 3 | 68286914 | 69276762 | 644 | -0.00025813 | 3.49E-09 | 5.91E-05 | -4.3695 | 1.25E-05 |
| 14 | 91296860 | 93132299 | 1147 | -0.00022489 | 2.66E-09 | 5.15E-05 | -4.364 | 1.28E-05 |
| 1 | 96150893 | 97885249 | 1053 | -0.00027176 | 3.91E-09 | 6.25E-05 | -4.3447 | 1.39E-05 |
| 4 | 117276929 | 118479918 | 959 | -0.00017184 | 1.57E-09 | 3.96E-05 | -4.3407 | 1.42E-05 |
| 18 | 47730584 | 51062185 | 2334 | -0.00033663 | 6.02E-09 | 7.76E-05 | -4.3379 | 1.44E-05 |
| 9 | 90136246 | 92528698 | 1515 | -0.00023715 | 2.99E-09 | 5.47E-05 | -4.3355 | 1.45E-05 |
| 5 | 144943354 | 147181998 | 1372 | -0.00023274 | 2.93E-09 | 5.41E-05 | -4.3031 | 1.68E-05 |
| 5 | 103320005 | 104851483 | 963 | -0.00028841 | 4.52E-09 | 6.73E-05 | -4.2879 | 1.80E-05 |
| 9 | 85440801 | 86938196 | 1202 | -0.0002544 | 3.55E-09 | 5.95E-05 | -4.2723 | 1.93E-05 |
| 5 | 75798866 | 77623332 | 1200 | -0.00021879 | 2.63E-09 | 5.12E-05 | -4.2693 | 1.96E-05 |
| 10 | 12586797 | 13321600 | 695 | -0.00029166 | 4.69E-09 | 6.85E-05 | -4.2591 | 2.05E-05 |
| 2 | 110572432 | 113921856 | 1302 | -0.00024769 | 3.40E-09 | 5.83E-05 | -4.2506 | 2.13E-05 |
| 7 | 1353067 | 2062398 | 233 | -0.00041265 | 9.43E-09 | 9.71E-05 | -4.2505 | 2.13E-05 |
| 20 | 52472549 | 54055266 | 1155 | -0.0002153 | 2.59E-09 | 5.09E-05 | -4.233 | 2.31E-05 |
| 5 | 3361941 | 4632110 | 1001 | -0.00024207 | 3.32E-09 | 5.76E-05 | -4.2 | 2.67E-05 |
| 4 | 161826435 | 163243679 | 993 | -0.00020843 | 2.48E-09 | 4.98E-05 | -4.1837 | 2.87E-05 |
| 2 | 60292000 | 62429044 | 928 | -0.00033535 | 6.44E-09 | 8.02E-05 | -4.1789 | 2.93E-05 |

1. CPD: Cigarettes smoked per day; GERD: Gastroesophageal reflux disease.

2. K is the number of eigenvectors used in the truncated singular value decomposition (SVD), local_rhog represents local genetic covariance estimates, and var stands for variance estimates.

# Table S6: Local genetic correlation between ASI and GERD

| **chr** | **start** | **end** | **num_snp** | **local_rhog** | **var** | **se** | **z** | **p** |
| --- | --- | --- | --- | --- | --- | --- | --- | --- |
| 11 | 112459488 | 114257728 | 1392 | -0.00070544 | 7.69E-09 | 8.77E-05 | -8.0439 | 8.70E-16 |
| 7 | 113712387 | 116780178 | 1459 | -0.00062301 | 6.67E-09 | 8.17E-05 | -7.6256 | 2.43E-14 |
| 6 | 33236497 | 35455756 | 1124 | -0.00052178 | 6.72E-09 | 8.20E-05 | -6.3642 | 1.96E-10 |
| 2 | 58297315 | 60292000 | 1221 | -0.00040521 | 4.28E-09 | 6.54E-05 | -6.1938 | 5.87E-10 |
| 2 | 144519484 | 146445570 | 796 | -0.00042027 | 5.40E-09 | 7.35E-05 | -5.7186 | 1.07E-08 |
| 10 | 104380410 | 106695048 | 1308 | -0.00048232 | 7.39E-09 | 8.60E-05 | -5.6088 | 2.04E-08 |
| 6 | 164383521 | 165586864 | 1187 | -0.00037635 | 4.58E-09 | 6.77E-05 | -5.5583 | 2.72E-08 |
| 5 | 91840542 | 93809984 | 740 | -0.00034017 | 3.85E-09 | 6.20E-05 | -5.484 | 4.16E-08 |
| 17 | 16412342 | 18856320 | 874 | -0.00032 | 3.46E-09 | 5.88E-05 | -5.4383 | 5.38E-08 |
| 16 | 71054028 | 72935150 | 689 | -0.0003193 | 3.52E-09 | 5.94E-05 | -5.3797 | 7.46E-08 |
| 16 | 1207206 | 2764829 | 518 | -0.00026079 | 2.54E-09 | 5.04E-05 | -5.1773 | 2.25E-07 |
| 3 | 157312028 | 159477890 | 1331 | -0.00032904 | 4.16E-09 | 6.45E-05 | -5.1034 | 3.34E-07 |
| 2 | 228119842 | 229370787 | 843 | -0.00032012 | 3.95E-09 | 6.29E-05 | -5.0919 | 3.54E-07 |
| 4 | 2842979 | 3846040 | 416 | -0.00042603 | 7.18E-09 | 8.47E-05 | -5.0279 | 4.96E-07 |
| 12 | 73818454 | 76511314 | 1653 | -0.00028891 | 3.60E-09 | 6.00E-05 | -4.8149 | 1.47E-06 |
| 12 | 67181144 | 67909729 | 637 | -0.00030538 | 4.25E-09 | 6.52E-05 | -4.687 | 2.77E-06 |
| 3 | 84367479 | 85582231 | 622 | -0.00025092 | 2.90E-09 | 5.39E-05 | -4.6561 | 3.22E-06 |
| 1 | 57021728 | 58865399 | 1437 | -0.00031141 | 4.49E-09 | 6.70E-05 | -4.6469 | 3.37E-06 |
| 22 | 32664986 | 34494914 | 1603 | -0.00025611 | 3.05E-09 | 5.52E-05 | -4.6358 | 3.56E-06 |
| 5 | 35048970 | 36433954 | 942 | 0.00023102 | 2.51E-09 | 5.01E-05 | 4.6099 | 4.03E-06 |
| 12 | 51776494 | 53039004 | 986 | -0.00022376 | 2.36E-09 | 4.86E-05 | -4.6037 | 4.15E-06 |
| 4 | 139556511 | 141084631 | 758 | -0.00039537 | 7.46E-09 | 8.64E-05 | -4.5785 | 4.68E-06 |
| 1 | 74326907 | 76728135 | 1562 | -0.00035771 | 6.14E-09 | 7.83E-05 | -4.5669 | 4.95E-06 |
| 4 | 66600492 | 68059497 | 914 | -0.00033965 | 5.58E-09 | 7.47E-05 | -4.5486 | 5.40E-06 |
| 11 | 37116815 | 39382003 | 1392 | -0.00031872 | 4.93E-09 | 7.02E-05 | -4.5372 | 5.70E-06 |
| 6 | 80691170 | 81929222 | 1016 | -0.00018899 | 1.75E-09 | 4.18E-05 | -4.5198 | 6.19E-06 |
| 11 | 62223771 | 63804569 | 435 | -0.00026094 | 3.34E-09 | 5.78E-05 | -4.5128 | 6.40E-06 |
| 1 | 37549183 | 38731847 | 564 | -0.00031775 | 5.11E-09 | 7.15E-05 | -4.4454 | 8.77E-06 |
| 2 | 199311125 | 201576284 | 1137 | -0.00031516 | 5.05E-09 | 7.11E-05 | -4.4349 | 9.21E-06 |
| 16 | 60745694 | 62229849 | 749 | -0.00025407 | 3.33E-09 | 5.77E-05 | -4.404 | 1.06E-05 |
| 13 | 90521311 | 93129691 | 1909 | -0.00024767 | 3.18E-09 | 5.64E-05 | -4.3905 | 1.13E-05 |
| 17 | 29786491 | 31538425 | 874 | -0.00026224 | 3.59E-09 | 5.99E-05 | -4.379 | 1.19E-05 |
| 4 | 27965868 | 29762208 | 1306 | -0.00029607 | 4.58E-09 | 6.76E-05 | -4.3773 | 1.20E-05 |
| 10 | 106695048 | 108726686 | 1516 | -0.00031809 | 5.33E-09 | 7.30E-05 | -4.3566 | 1.32E-05 |
| 18 | 39892648 | 42922106 | 1994 | -0.00028849 | 4.41E-09 | 6.64E-05 | -4.342 | 1.41E-05 |
| 14 | 96262415 | 98341162 | 1388 | -0.00021116 | 2.38E-09 | 4.88E-05 | -4.3239 | 1.53E-05 |
| 5 | 175450595 | 177251507 | 468 | -0.00026521 | 3.77E-09 | 6.14E-05 | -4.3205 | 1.56E-05 |
| 3 | 167117429 | 168580960 | 708 | -0.00020431 | 2.30E-09 | 4.79E-05 | -4.2629 | 2.02E-05 |
| 5 | 156628700 | 158825698 | 1664 | -0.00028192 | 4.40E-09 | 6.63E-05 | -4.2492 | 2.15E-05 |
| 3 | 88298373 | 94255316 | 1143 | -0.00023675 | 3.12E-09 | 5.59E-05 | -4.2352 | 2.28E-05 |
| 13 | 100574095 | 102300523 | 1368 | -0.00028499 | 4.54E-09 | 6.74E-05 | -4.2283 | 2.35E-05 |
| 17 | 55357541 | 57487512 | 1007 | -0.00026969 | 4.12E-09 | 6.42E-05 | -4.2024 | 2.64E-05 |
| 8 | 144236881 | 146303867 | 514 | -0.00023764 | 3.21E-09 | 5.67E-05 | -4.1919 | 2.77E-05 |
| 2 | 225840004 | 228119842 | 1495 | -0.00027946 | 4.45E-09 | 6.67E-05 | -4.1877 | 2.82E-05 |

1. ASI: Age of smoking initiation; GERD: Gastroesophageal reflux disease.

2. K is the number of eigenvectors used in the truncated singular value decomposition (SVD), local_rhog represents local genetic covariance estimates, and var stands for variance estimates.

# Table S7: Cross-trait GWAS meta-analysis

| **SNP** | **CHR** | **BP** | **ALT/**  **REF** | **MTAG** | | | | | | **CPASSOC** | | | | | |
| --- | --- | --- | --- | --- | --- | --- | --- | --- | --- | --- | --- | --- | --- | --- | --- |
|  |  |  |  | **Beta**  **(X)** | **Se**  **(X)** | **Pval**  **(X)** | **Beta**  **GERD** | **Se**  **GERD** | **Pval**  **GERD** | **Beta**  **(X)** | **Se**  **(X)** | **Pval**  **(X)** | **Beta**  **GERD** | **Se**  **GERD** | **Pval**  **GERD** |
| **SI** |  |  |  |  |  |  |  |  |  |  |  |  |  |  |  |
| rs10212155 | 3 | 117785505 | A/G | 0.02 | 2.27E-03 | 6.21E-15 | -0.05 | 7.21E-03 | 2.58E-10 | 0.03 | 4.93E-03 | 5.57E-11 | -0.03 | 6.79E-03 | 1.13E-05 |
| rs1022732 | 20 | 31152094 | C/T | 0.01 | 1.62E-03 | 4.71E-12 | -0.03 | 5.48E-03 | 3.55E-08 | 0.02 | 3.77E-03 | 5.11E-09 | -0.02 | 5.16E-03 | 0.000147 |
| rs10238965 | 7 | 99145845 | T/C | -0.01 | 1.96E-03 | 2.02E-12 | 0.04 | 7.08E-03 | 3.41E-08 | -0.03 | 4.91E-03 | 2.46E-09 | 0.03 | 6.67E-03 | 0.00016 |
| rs10764330 | 10 | 22286351 | G/A | -0.01 | 1.68E-03 | 4.85E-12 | 0.03 | 5.61E-03 | 4.53E-08 | -0.02 | 3.88E-03 | 5.06E-09 | 0.02 | 5.28E-03 | 0.000173 |
| rs10807752 | 7 | 1883486 | C/T | -0.01 | 1.58E-03 | 4.55E-14 | 0.04 | 5.17E-03 | 4.33E-12 | -0.02 | 3.60E-03 | 9.88E-10 | 0.02 | 4.87E-03 | 3.75E-07 |
| rs11030387 | 11 | 28631484 | T/C | -0.01 | 1.59E-03 | 1.99E-11 | 0.03 | 5.22E-03 | 1.93E-08 | -0.02 | 3.63E-03 | 2.38E-08 | 0.02 | 4.92E-03 | 7.34E-05 |
| rs11191267 | 10 | 104112737 | C/T | -0.01 | 1.61E-03 | 1.15E-12 | 0.03 | 5.28E-03 | 1.25E-08 | -0.02 | 3.66E-03 | 1.97E-09 | 0.02 | 4.97E-03 | 8.38E-05 |
| rs11210177 | 1 | 73655821 | G/A | 0.01 | 1.56E-03 | 9.40E-14 | -0.03 | 5.11E-03 | 2.02E-08 | 0.02 | 3.56E-03 | 1.66E-10 | -0.02 | 4.81E-03 | 0.000172 |
| rs11210860 | 1 | 43982527 | A/G | -0.01 | 1.63E-03 | 1.58E-18 | 0.04 | 5.26E-03 | 3.04E-13 | -0.03 | 3.67E-03 | 2.22E-13 | 0.03 | 4.95E-03 | 2.99E-07 |
| rs11210887 | 1 | 44076019 | A/G | -0.02 | 1.66E-03 | 3.81E-22 | 0.03 | 5.57E-03 | 3.87E-10 | -0.03 | 3.89E-03 | 8.26E-18 | 0.02 | 5.25E-03 | 0.000128 |
| rs11214444 | 11 | 112863795 | T/C | 0.02 | 2.44E-03 | 3.69E-15 | -0.06 | 7.94E-03 | 1.43E-13 | 0.04 | 5.59E-03 | 2.74E-10 | -0.04 | 7.48E-03 | 4.40E-08 |
| rs11599236 | 10 | 106454672 | C/T | -0.01 | 1.57E-03 | 3.89E-09 | 0.04 | 5.24E-03 | 5.02E-14 | -0.01 | 3.61E-03 | 3.38E-05 | 0.03 | 4.92E-03 | 8.13E-10 |
| rs11712680 | 3 | 75009019 | C/A | -0.01 | 2.06E-03 | 2.54E-12 | 0.04 | 6.56E-03 | 2.11E-08 | -0.03 | 4.58E-03 | 3.51E-09 | 0.02 | 6.17E-03 | 0.000108 |
| rs12453010 | 17 | 50316131 | T/C | 0.01 | 1.61E-03 | 3.62E-09 | -0.04 | 5.25E-03 | 1.22E-13 | 0.02 | 3.64E-03 | 2.70E-05 | -0.03 | 4.93E-03 | 1.75E-09 |
| rs13261666 | 8 | 59814666 | T/G | -0.01 | 1.56E-03 | 2.55E-18 | 0.03 | 5.11E-03 | 2.82E-10 | -0.03 | 3.56E-03 | 3.90E-14 | 0.02 | 4.81E-03 | 3.62E-05 |
| rs1431196 | 18 | 50832102 | G/A | 0.01 | 1.59E-03 | 1.74E-10 | -0.04 | 5.18E-03 | 3.12E-16 | 0.02 | 3.58E-03 | 7.16E-06 | -0.03 | 4.86E-03 | 2.65E-11 |
| rs1526480 | 1 | 91209986 | C/T | 0.01 | 1.59E-03 | 7.12E-13 | -0.03 | 5.28E-03 | 8.15E-09 | 0.02 | 3.66E-03 | 1.48E-09 | -0.02 | 4.99E-03 | 6.60E-05 |
| rs17515489 | 11 | 112709813 | T/C | 0.01 | 1.76E-03 | 6.29E-12 | -0.03 | 5.58E-03 | 2.57E-08 | 0.02 | 3.89E-03 | 7.61E-09 | -0.02 | 5.26E-03 | 0.000109 |
| rs17684592 | 7 | 126507903 | A/C | 0.01 | 1.66E-03 | 4.98E-08 | -0.04 | 5.31E-03 | 1.36E-12 | 0.01 | 3.72E-03 | 0.00014 | -0.03 | 5.01E-03 | 6.35E-09 |
| rs200968 | 6 | 27859568 | C/T | -0.01 | 2.09E-03 | 1.96E-10 | 0.05 | 6.45E-03 | 5.24E-16 | -0.02 | 4.62E-03 | 7.13E-06 | 0.04 | 6.08E-03 | 3.94E-11 |
| rs2132660 | 15 | 47679807 | G/A | 0.02 | 1.90E-03 | 5.55E-18 | -0.04 | 6.26E-03 | 1.03E-10 | 0.03 | 4.33E-03 | 1.14E-13 | -0.03 | 5.88E-03 | 1.62E-05 |
| rs2179152 | 6 | 26325888 | C/T | -0.01 | 1.64E-03 | 6.40E-10 | 0.04 | 5.26E-03 | 4.03E-17 | -0.02 | 3.68E-03 | 2.65E-05 | 0.03 | 4.96E-03 | 3.19E-12 |
| rs2232423 | 6 | 28366151 | G/A | -0.02 | 2.95E-03 | 5.33E-11 | 0.07 | 7.95E-03 | 9.74E-17 | -0.03 | 5.87E-03 | 3.36E-06 | 0.05 | 7.49E-03 | 1.37E-11 |
| rs2240326 | 3 | 50128386 | A/G | -0.01 | 1.56E-03 | 4.22E-09 | 0.06 | 5.11E-03 | 1.33E-28 | -0.01 | 3.56E-03 | 0.00195 | 0.05 | 4.81E-03 | 1.13E-22 |
| rs266059 | 2 | 104084908 | G/A | -0.01 | 1.57E-03 | 7.74E-21 | 0.03 | 5.11E-03 | 3.59E-11 | -0.03 | 3.74E-03 | 3.55E-16 | 0.02 | 4.82E-03 | 1.84E-05 |
| rs2815749 | 1 | 72814783 | G/A | 0.01 | 2.04E-03 | 4.03E-11 | -0.05 | 6.40E-03 | 1.04E-15 | 0.02 | 4.48E-03 | 1.84E-06 | -0.04 | 6.02E-03 | 1.07E-10 |
| rs3172494 | 3 | 48731487 | T/G | -0.02 | 2.46E-03 | 1.82E-10 | 0.06 | 8.30E-03 | 2.44E-13 | -0.03 | 5.66E-03 | 2.20E-06 | 0.05 | 7.82E-03 | 6.71E-09 |
| rs329124 | 5 | 133865452 | G/A | -0.01 | 1.58E-03 | 3.64E-08 | 0.04 | 5.18E-03 | 5.91E-13 | -0.01 | 3.59E-03 | 0.000123 | 0.03 | 4.88E-03 | 3.44E-09 |
| rs3766823 | 1 | 32197257 | A/G | 0.01 | 1.97E-03 | 2.87E-10 | -0.05 | 6.78E-03 | 1.87E-14 | 0.02 | 4.70E-03 | 5.22E-06 | -0.04 | 6.39E-03 | 7.09E-10 |
| rs3922717 | 6 | 27030924 | G/A | -0.01 | 1.92E-03 | 1.78E-10 | 0.05 | 5.99E-03 | 3.18E-18 | -0.02 | 4.25E-03 | 1.47E-05 | 0.04 | 5.64E-03 | 5.35E-13 |
| rs4300861 | 2 | 22549441 | T/C | 0.01 | 1.62E-03 | 1.62E-10 | -0.04 | 5.26E-03 | 1.13E-14 | 0.02 | 3.67E-03 | 3.60E-06 | -0.03 | 4.95E-03 | 5.43E-10 |
| rs4382592*# | 9 | 134870755 | G/T | -0.01 | 1.67E-03 | 8.03E-11 | 0.04 | 5.57E-03 | 2.47E-13 | -0.02 | 3.86E-03 | 1.15E-06 | 0.03 | 5.25E-03 | 8.20E-09 |
| rs465646 | 6 | 111620758 | A/G | -0.02 | 2.10E-03 | 4.60E-22 | 0.05 | 6.98E-03 | 9.52E-12 | -0.04 | 4.84E-03 | 4.00E-17 | 0.03 | 6.58E-03 | 1.09E-05 |
| rs4855845 | 3 | 49687043 | C/T | -0.02 | 2.05E-03 | 3.17E-14 | 0.06 | 6.69E-03 | 4.32E-21 | -0.03 | 4.70E-03 | 5.61E-08 | 0.05 | 6.30E-03 | 2.35E-14 |
| rs6265 | 11 | 27679916 | T/C | -0.02 | 1.94E-03 | 9.77E-16 | 0.04 | 6.52E-03 | 4.87E-09 | -0.03 | 4.58E-03 | 3.77E-12 | 0.02 | 6.15E-03 | 0.000118 |
| rs6441814 | 3 | 44049114 | A/G | -0.01 | 1.57E-03 | 6.67E-10 | 0.04 | 5.13E-03 | 1.86E-13 | -0.02 | 3.56E-03 | 6.52E-06 | 0.03 | 4.83E-03 | 3.86E-09 |
| rs6690398 | 1 | 66447394 | A/G | 0.01 | 1.57E-03 | 2.15E-19 | -0.04 | 5.17E-03 | 3.14E-17 | 0.03 | 3.58E-03 | 4.52E-13 | -0.03 | 4.86E-03 | 4.40E-10 |
| rs6728726 | 2 | 623976 | C/T | 0.02 | 2.08E-03 | 6.29E-19 | -0.05 | 6.76E-03 | 9.14E-13 | 0.04 | 4.73E-03 | 6.73E-14 | -0.03 | 6.37E-03 | 7.56E-07 |
| rs6965740 | 7 | 117514840 | G/T | 0.01 | 1.57E-03 | 3.65E-16 | -0.03 | 5.12E-03 | 6.42E-09 | 0.03 | 3.58E-03 | 1.36E-12 | -0.02 | 4.82E-03 | 0.000162 |
| rs7241572* | 18 | 77580712 | A/G | 0.01 | 2.01E-03 | 1.50E-10 | -0.05 | 6.36E-03 | 2.09E-14 | 0.02 | 4.39E-03 | 3.02E-06 | -0.04 | 5.97E-03 | 9.49E-10 |
| rs7309 | 2 | 162092640 | A/G | 0.01 | 1.57E-03 | 1.04E-11 | -0.04 | 5.13E-03 | 6.21E-14 | 0.02 | 3.56E-03 | 2.80E-07 | -0.03 | 4.82E-03 | 4.37E-09 |
| rs773109 | 12 | 56374695 | A/G | -0.01 | 1.70E-03 | 9.52E-12 | 0.05 | 5.41E-03 | 1.36E-19 | -0.02 | 3.78E-03 | 2.59E-06 | 0.04 | 5.10E-03 | 8.71E-14 |
| rs7735362 | 5 | 104006833 | A/G | -0.01 | 1.93E-03 | 1.16E-11 | 0.04 | 6.53E-03 | 7.26E-09 | -0.03 | 4.51E-03 | 1.92E-08 | 0.03 | 6.16E-03 | 3.89E-05 |
| rs7938812 | 11 | 112911004 | G/T | 0.02 | 1.58E-03 | 2.62E-41 | -0.04 | 5.25E-03 | 3.56E-17 | 0.04 | 3.64E-03 | 2.71E-33 | -0.02 | 4.94E-03 | 4.75E-07 |
| rs9379901 | 6 | 26603866 | C/T | -0.01 | 2.45E-03 | 3.64E-09 | 0.06 | 7.51E-03 | 9.03E-14 | -0.02 | 5.43E-03 | 2.89E-05 | 0.04 | 7.07E-03 | 1.34E-09 |
| rs9467800 | 6 | 26578525 | C/A | 0.01 | 1.57E-03 | 4.45E-08 | -0.04 | 5.12E-03 | 2.48E-16 | 0.01 | 3.57E-03 | 0.000457 | -0.03 | 4.82E-03 | 4.00E-12 |
| rs9485410 | 6 | 101261007 | C/T | -0.01 | 1.56E-03 | 2.27E-12 | 0.03 | 5.12E-03 | 6.42E-09 | -0.02 | 3.56E-03 | 4.39E-09 | 0.02 | 4.82E-03 | 4.65E-05 |
| rs9636202 | 19 | 18449238 | A/G | -0.01 | 1.80E-03 | 1.48E-10 | 0.05 | 5.80E-03 | 2.34E-15 | -0.02 | 4.06E-03 | 4.47E-06 | 0.04 | 5.47E-03 | 1.51E-10 |
| rs9671376*# | 14 | 103370163 | T/C | 0.01 | 1.80E-03 | 3.89E-08 | -0.04 | 6.20E-03 | 2.14E-12 | 0.02 | 4.30E-03 | 0.000107 | -0.03 | 5.84E-03 | 1.00E-08 |
| rs9837341 | 3 | 49664767 | G/A | -0.01 | 1.70E-03 | 3.01E-08 | 0.05 | 5.58E-03 | 1.61E-21 | -0.01 | 3.87E-03 | 0.00142 | 0.04 | 5.26E-03 | 1.20E-16 |
| rs993700 | 4 | 67825894 | C/T | -0.01 | 1.85E-03 | 5.00E-13 | 0.04 | 6.15E-03 | 2.34E-09 | -0.03 | 4.29E-03 | 1.53E-09 | 0.02 | 5.78E-03 | 2.80E-05 |
| **NS** |  |  |  |  |  |  |  |  |  |  |  |  |  |  |  |
| rs1017550 | 10 | 63587683 | G/A | 0.02 | 2.19E-03 | 1.36E-13 | 0.03 | 5.20E-03 | 2.27E-10 | 0.01 | 1.18E-03 | 6.17E-11 | 0.02 | 4.88E-03 | 8.08E-06 |
| rs11210887 | 1 | 44076019 | A/G | 0.02 | 2.36E-03 | 9.23E-13 | 0.03 | 5.60E-03 | 1.50E-08 | 0.01 | 1.27E-03 | 8.00E-11 | 0.02 | 5.25E-03 | 0.000128 |
| rs11599236 | 10 | 106454672 | C/T | 0.01 | 2.20E-03 | 1.09E-11 | 0.04 | 5.26E-03 | 7.12E-15 | 0.01 | 1.19E-03 | 2.05E-07 | 0.03 | 4.92E-03 | 8.13E-10 |
| rs11766215 | 7 | 1821725 | A/G | 0.01 | 2.56E-03 | 3.46E-08 | 0.05 | 6.09E-03 | 5.62E-16 | 0.00 | 1.38E-03 | 0.000486 | 0.04 | 5.71E-03 | 7.43E-12 |
| rs12357321 | 10 | 21790476 | A/G | -0.02 | 2.33E-03 | 6.46E-11 | -0.04 | 5.59E-03 | 2.30E-14 | -0.01 | 1.27E-03 | 8.08E-07 | -0.03 | 5.23E-03 | 1.33E-09 |
| rs12453010 | 17 | 50316131 | T/C | -0.01 | 2.21E-03 | 3.42E-08 | -0.04 | 5.27E-03 | 2.77E-13 | 0.00 | 1.19E-03 | 0.000136 | -0.03 | 4.93E-03 | 1.75E-09 |
| rs1357309 | 7 | 3486358 | C/T | 0.02 | 2.38E-03 | 2.57E-12 | 0.04 | 5.66E-03 | 3.96E-10 | 0.01 | 1.28E-03 | 1.20E-09 | 0.02 | 5.30E-03 | 7.13E-06 |
| rs1499982 | 3 | 117820386 | T/C | -0.02 | 3.04E-03 | 6.12E-15 | -0.05 | 7.26E-03 | 9.20E-11 | -0.01 | 1.64E-03 | 3.00E-12 | -0.03 | 6.80E-03 | 7.34E-06 |
| rs1510719 | 4 | 140938116 | C/T | 0.02 | 2.22E-03 | 1.20E-12 | 0.05 | 5.28E-03 | 1.92E-21 | 0.01 | 1.20E-03 | 1.11E-06 | 0.04 | 4.95E-03 | 3.84E-15 |
| rs17114682 | 11 | 112888129 | C/A | -0.03 | 3.36E-03 | 2.43E-16 | -0.06 | 7.98E-03 | 4.51E-14 | -0.01 | 1.80E-03 | 2.03E-12 | -0.04 | 7.48E-03 | 4.07E-08 |
| rs1937450 | 1 | 66478840 | G/T | -0.01 | 2.17E-03 | 5.45E-12 | -0.04 | 5.18E-03 | 3.09E-16 | -0.01 | 1.17E-03 | 2.49E-07 | -0.03 | 4.84E-03 | 7.07E-11 |
| rs200968*# | 6 | 27859568 | C/T | 0.02 | 2.73E-03 | 1.04E-09 | 0.05 | 6.48E-03 | 9.84E-16 | 0.01 | 1.47E-03 | 2.20E-05 | 0.04 | 6.08E-03 | 3.94E-11 |
| rs2179152 | 6 | 26325888 | C/T | 0.01 | 2.23E-03 | 1.15E-09 | 0.04 | 5.29E-03 | 5.69E-17 | 0.00 | 1.20E-03 | 4.47E-05 | 0.03 | 4.96E-03 | 3.19E-12 |
| rs2232423 | 6 | 28366151 | G/A | 0.02 | 3.35E-03 | 2.24E-11 | 0.07 | 7.99E-03 | 7.39E-17 | 0.01 | 1.80E-03 | 1.30E-06 | 0.05 | 7.49E-03 | 1.37E-11 |
| rs2681780 | 3 | 49897830 | T/C | -0.01 | 2.16E-03 | 2.35E-08 | -0.06 | 5.14E-03 | 2.70E-27 | 0.00 | 1.16E-03 | 0.014272 | -0.05 | 4.81E-03 | 3.75E-22 |
| rs2815749 | 1 | 72814783 | G/A | -0.02 | 2.70E-03 | 5.90E-11 | -0.05 | 6.43E-03 | 1.15E-15 | -0.01 | 1.45E-03 | 1.60E-06 | -0.04 | 6.02E-03 | 1.07E-10 |
| rs3766823 | 1 | 32197257 | A/G | -0.02 | 2.86E-03 | 2.96E-09 | -0.05 | 6.81E-03 | 4.08E-14 | -0.01 | 1.54E-03 | 2.37E-05 | -0.04 | 6.39E-03 | 7.09E-10 |
| rs3851570 | 11 | 112742098 | G/A | -0.02 | 2.36E-03 | 7.69E-12 | -0.03 | 5.61E-03 | 1.85E-08 | -0.01 | 1.27E-03 | 7.90E-10 | -0.02 | 5.27E-03 | 0.000106 |
| rs3922717* | 6 | 27030924 | G/A | 0.01 | 2.53E-03 | 3.27E-09 | 0.05 | 6.02E-03 | 1.12E-17 | 0.01 | 1.36E-03 | 0.000148 | 0.04 | 5.64E-03 | 5.35E-13 |
| rs4300861 | 2 | 22549441 | T/C | -0.01 | 2.22E-03 | 3.98E-09 | -0.04 | 5.29E-03 | 3.32E-14 | 0.00 | 1.20E-03 | 3.26E-05 | -0.03 | 4.95E-03 | 5.43E-10 |
| rs4855845 | 3 | 49687043 | C/T | 0.02 | 2.83E-03 | 2.42E-10 | 0.06 | 6.73E-03 | 1.16E-19 | 0.01 | 1.52E-03 | 4.20E-05 | 0.05 | 6.30E-03 | 2.35E-14 |
| rs6265 | 11 | 27679916 | T/C | 0.02 | 2.76E-03 | 8.84E-14 | 0.04 | 6.56E-03 | 7.89E-09 | 0.01 | 1.48E-03 | 7.61E-12 | 0.02 | 6.15E-03 | 0.000118 |
| rs650599 | 9 | 127849318 | C/A | 0.02 | 2.33E-03 | 4.70E-11 | 0.03 | 5.52E-03 | 2.97E-08 | 0.01 | 1.25E-03 | 4.77E-09 | 0.02 | 5.17E-03 | 0.000109 |
| rs6543227 | 2 | 104250376 | G/A | -0.02 | 2.16E-03 | 1.01E-18 | -0.04 | 5.13E-03 | 4.47E-13 | -0.01 | 1.16E-03 | 1.98E-15 | -0.02 | 4.81E-03 | 6.23E-07 |
| rs6965740 | 7 | 117514840 | G/T | -0.02 | 2.17E-03 | 3.69E-12 | -0.03 | 5.15E-03 | 2.81E-08 | -0.01 | 1.17E-03 | 2.85E-10 | -0.02 | 4.82E-03 | 0.000162 |
| rs7241572* | 18 | 77580712 | A/G | -0.02 | 2.66E-03 | 8.37E-11 | -0.05 | 6.40E-03 | 1.64E-14 | -0.01 | 1.45E-03 | 1.14E-06 | -0.04 | 5.97E-03 | 9.49E-10 |
| rs7309 | 2 | 162092640 | A/G | -0.01 | 2.16E-03 | 4.53E-11 | -0.04 | 5.16E-03 | 8.57E-14 | -0.01 | 1.17E-03 | 3.97E-07 | -0.03 | 4.82E-03 | 4.37E-09 |
| rs7567570 | 2 | 615140 | C/T | -0.02 | 2.85E-03 | 2.57E-12 | -0.04 | 6.78E-03 | 5.45E-11 | -0.01 | 1.53E-03 | 2.58E-09 | -0.03 | 6.36E-03 | 1.52E-06 |
| rs7766610 | 6 | 111707821 | A/C | 0.03 | 2.80E-03 | 8.47E-21 | 0.05 | 6.65E-03 | 3.89E-12 | 0.01 | 1.51E-03 | 3.07E-18 | 0.03 | 6.22E-03 | 6.23E-06 |
| rs7948789 | 11 | 112839532 | G/A | -0.03 | 2.22E-03 | 1.57E-43 | -0.05 | 5.28E-03 | 2.59E-18 | -0.02 | 1.20E-03 | 1.33E-41 | -0.03 | 4.95E-03 | 4.00E-07 |
| rs8032333 | 15 | 47680567 | G/A | -0.02 | 2.64E-03 | 9.76E-13 | -0.04 | 6.29E-03 | 9.72E-10 | -0.01 | 1.42E-03 | 2.90E-10 | -0.03 | 5.88E-03 | 1.69E-05 |
| rs883403 | 7 | 99047978 | C/T | 0.02 | 2.99E-03 | 4.98E-11 | 0.04 | 7.10E-03 | 3.81E-08 | 0.01 | 1.61E-03 | 4.55E-09 | 0.03 | 6.66E-03 | 0.00013 |
| rs889398 | 16 | 69556715 | T/C | 0.01 | 2.20E-03 | 1.28E-11 | 0.03 | 5.23E-03 | 1.42E-09 | 0.01 | 1.18E-03 | 4.11E-09 | 0.02 | 4.90E-03 | 1.40E-05 |
| rs9379901 | 6 | 26603866 | C/T | 0.02 | 3.17E-03 | 4.87E-10 | 0.06 | 7.55E-03 | 4.62E-14 | 0.01 | 1.70E-03 | 4.40E-06 | 0.04 | 7.07E-03 | 1.34E-09 |
| rs9381917 | 6 | 50911334 | A/G | 0.02 | 3.57E-03 | 2.76E-12 | 0.06 | 8.47E-03 | 1.51E-11 | 0.01 | 1.92E-03 | 4.47E-09 | 0.04 | 7.94E-03 | 5.53E-07 |
| **ES** |  |  |  |  |  |  |  |  |  |  |  |  |  |  |  |
| rs10212155 | 3 | 117785505 | A/G | 0.02 | 2.87E-03 | 4.76E-15 | -0.04 | 7.49E-03 | 4.52E-09 | 0.01 | 1.42E-03 | 3.40E-13 | -0.03 | 6.79E-03 | 1.13E-05 |
| rs10274465 | 7 | 3493590 | C/T | -0.01 | 2.12E-03 | 3.64E-11 | 0.03 | 5.53E-03 | 1.40E-09 | -0.01 | 1.05E-03 | 3.20E-09 | 0.02 | 5.02E-03 | 2.01E-06 |
| rs10789932 | 11 | 112847196 | A/C | 0.02 | 3.17E-03 | 1.86E-14 | -0.06 | 8.25E-03 | 3.61E-12 | 0.01 | 1.56E-03 | 6.70E-12 | -0.04 | 7.48E-03 | 5.13E-08 |
| rs13196692 | 6 | 27379119 | T/C | -0.02 | 3.25E-03 | 4.63E-08 | 0.06 | 8.39E-03 | 5.77E-13 | -0.01 | 1.60E-03 | 9.60E-06 | 0.05 | 7.61E-03 | 1.84E-09 |
| rs1510719*# | 4 | 140938116 | C/T | -0.01 | 2.10E-03 | 1.41E-11 | 0.05 | 5.46E-03 | 7.75E-21 | -0.01 | 1.04E-03 | 7.60E-08 | 0.04 | 4.95E-03 | 3.84E-15 |
| rs161645 | 5 | 104069917 | G/A | -0.01 | 2.18E-03 | 4.15E-08 | 0.04 | 5.66E-03 | 1.54E-13 | 0.00 | 1.07E-03 | 1.00E-05 | 0.03 | 5.14E-03 | 6.56E-10 |
| rs17488728 | 7 | 117544180 | T/C | 0.02 | 2.04E-03 | 6.16E-14 | -0.03 | 5.32E-03 | 1.84E-08 | 0.01 | 1.01E-03 | 3.10E-12 | -0.02 | 4.83E-03 | 2.47E-05 |
| rs1937450 | 1 | 66478840 | G/T | 0.01 | 2.04E-03 | 4.48E-09 | -0.04 | 5.36E-03 | 5.77E-15 | 0.00 | 1.01E-03 | 2.20E-06 | -0.03 | 4.84E-03 | 7.07E-11 |
| rs2232423 | 6 | 28366151 | G/A | -0.02 | 3.20E-03 | 2.74E-08 | 0.07 | 8.25E-03 | 1.12E-15 | -0.01 | 1.58E-03 | 1.30E-05 | 0.05 | 7.49E-03 | 1.37E-11 |
| rs2624833 | 3 | 50212512 | T/G | 0.01 | 2.15E-03 | 1.62E-08 | -0.05 | 5.61E-03 | 6.55E-20 | 0.00 | 1.07E-03 | 2.40E-05 | -0.04 | 5.09E-03 | 5.82E-15 |
| rs264921 | 2 | 104273554 | G/A | -0.02 | 2.04E-03 | 1.85E-15 | 0.03 | 5.30E-03 | 4.71E-11 | -0.01 | 1.00E-03 | 4.00E-13 | 0.02 | 4.81E-03 | 4.34E-07 |
| rs329122 | 5 | 133864599 | A/G | -0.01 | 2.06E-03 | 2.24E-08 | 0.04 | 5.38E-03 | 9.39E-13 | 0.00 | 1.02E-03 | 4.70E-06 | 0.03 | 4.88E-03 | 3.05E-09 |
| rs359233 | 2 | 60470926 | G/A | 0.01 | 2.11E-03 | 6.29E-11 | -0.03 | 5.50E-03 | 2.13E-08 | 0.01 | 1.04E-03 | 3.10E-09 | -0.02 | 5.00E-03 | 1.46E-05 |
| rs465646 | 6 | 111620758 | A/G | -0.03 | 2.78E-03 | 1.65E-19 | 0.04 | 7.25E-03 | 1.37E-09 | -0.01 | 1.37E-03 | 1.30E-17 | 0.03 | 6.58E-03 | 1.09E-05 |
| rs4855845 | 3 | 49687043 | C/T | -0.02 | 2.67E-03 | 5.09E-09 | 0.06 | 6.95E-03 | 2.80E-19 | -0.01 | 1.32E-03 | 7.90E-06 | 0.05 | 6.30E-03 | 2.35E-14 |
| rs7938812 | 11 | 112911004 | G/T | 0.03 | 2.09E-03 | 2.00E-48 | -0.04 | 5.45E-03 | 2.95E-14 | 0.01 | 1.03E-03 | 2.60E-46 | -0.02 | 4.94E-03 | 4.75E-07 |
| rs929175* | 7 | 126479218 | A/G | 0.01 | 2.12E-03 | 1.94E-08 | -0.04 | 5.53E-03 | 1.69E-12 | 0.00 | 1.05E-03 | 3.80E-06 | -0.03 | 5.02E-03 | 4.98E-09 |
| rs9393735 | 6 | 26582327 | G/A | -0.02 | 2.92E-03 | 3.81E-08 | 0.05 | 7.59E-03 | 2.57E-12 | -0.01 | 1.44E-03 | 6.60E-06 | 0.04 | 6.89E-03 | 6.22E-09 |
| **CPD** |  |  |  |  |  |  |  |  |  |  |  |  |  |  |  |
| rs10519203 | 15 | 78814046 | G/A | -0.07 | 2.47E-03 | 6.55E-170 | -0.04 | 5.55E-03 | 2.75E-14 | -0.18 | 5.87E-03 | 1.00E-200 | 0.00 | 5.09E-03 | 0.488657 |
| rs11762636 | 7 | 2061111 | C/A | -0.02 | 2.84E-03 | 7.54E-11 | -0.07 | 6.82E-03 | 5.60E-22 | -0.03 | 7.15E-03 | 0.000419 | 0.05 | 6.26E-03 | 1.88E-16 |
| rs12062845 | 1 | 98342685 | C/A | 0.02 | 2.82E-03 | 1.28E-08 | 0.06 | 6.36E-03 | 1.29E-18 | 0.02 | 6.62E-03 | 0.00415 | -0.04 | 5.87E-03 | 3.23E-14 |
| rs1392816 | 1 | 66481188 | C/T | -0.01 | 2.40E-03 | 4.38E-08 | -0.04 | 5.41E-03 | 5.57E-14 | -0.02 | 5.70E-03 | 0.00134 | 0.03 | 4.96E-03 | 2.64E-10 |
| rs1431196 | 18 | 50832102 | A/G | 0.01 | 2.35E-03 | 3.73E-08 | 0.04 | 5.31E-03 | 3.77E-15 | 0.02 | 5.54E-03 | 0.00168 | -0.03 | 4.86E-03 | 2.65E-11 |
| rs1941954 | 18 | 35159596 | A/C | -0.01 | 2.45E-03 | 4.60E-08 | -0.05 | 5.60E-03 | 1.49E-16 | -0.02 | 5.87E-03 | 0.00403 | 0.04 | 5.14E-03 | 1.44E-12 |
| rs2179152 | 6 | 26325888 | T/C | -0.02 | 2.43E-03 | 4.23E-10 | -0.05 | 5.40E-03 | 7.26E-17 | -0.02 | 5.68E-03 | 0.000161 | 0.03 | 4.96E-03 | 3.19E-12 |
| rs2350804 | 4 | 67075938 | C/T | -0.02 | 2.52E-03 | 1.36E-12 | -0.04 | 5.97E-03 | 2.07E-10 | -0.04 | 6.13E-03 | 6.76E-09 | 0.03 | 5.46E-03 | 2.61E-06 |
| rs3766823 | 1 | 32197257 | G/A | 0.02 | 2.92E-03 | 2.20E-08 | 0.05 | 6.95E-03 | 1.39E-13 | 0.03 | 7.29E-03 | 0.00044 | -0.04 | 6.39E-03 | 7.09E-10 |
| rs7125588 | 11 | 113436072 | A/G | -0.02 | 2.35E-03 | 2.88E-13 | -0.04 | 5.29E-03 | 7.39E-12 | -0.03 | 5.56E-03 | 4.27E-09 | 0.03 | 4.85E-03 | 2.48E-07 |
| rs8042374 | 15 | 78908032 | A/G | -0.06 | 2.69E-03 | 2.67E-105 | -0.05 | 6.24E-03 | 1.59E-13 | -0.16 | 6.68E-03 | 1.22E-123 | 0.01 | 5.73E-03 | 0.046316 |
| **ASI** |  |  |  |  |  |  |  |  |  |  |  |  |  |  |  |
| rs1021363 | 10 | 106610839 | G/A | 0.01 | 2.01E-03 | 7.01E-13 | 0.04 | 5.41E-03 | 3.36E-15 | 0.01 | 2.89E-03 | 2.55E-06 | 0.03 | 5.02E-03 | 5.10E-10 |
| rs10262103*# | 7 | 114091844 | A/C | -0.01 | 1.90E-03 | 5.71E-10 | -0.04 | 5.28E-03 | 7.04E-13 | -0.01 | 2.80E-03 | 0.000278 | -0.03 | 4.89E-03 | 5.64E-09 |
| rs11081564 | 18 | 77551586 | G/A | -0.01 | 2.24E-03 | 1.62E-08 | -0.04 | 6.01E-03 | 9.47E-13 | -0.01 | 3.12E-03 | 0.00841 | -0.03 | 5.55E-03 | 1.57E-09 |
| rs11762636 | 7 | 2061111 | A/C | 0.01 | 2.33E-03 | 4.49E-10 | 0.06 | 6.75E-03 | 4.10E-20 | 0.01 | 3.58E-03 | 0.123 | 0.05 | 6.26E-03 | 1.88E-16 |
| rs12967855 | 18 | 35138245 | G/A | 0.01 | 1.98E-03 | 6.63E-10 | 0.05 | 5.54E-03 | 1.72E-16 | 0.01 | 2.93E-03 | 0.0145 | 0.04 | 5.13E-03 | 1.09E-12 |
| rs1334297 | 13 | 58335375 | A/G | 0.01 | 2.19E-03 | 4.32E-09 | 0.05 | 5.88E-03 | 4.64E-16 | 0.01 | 3.09E-03 | 0.0433 | 0.04 | 5.46E-03 | 1.14E-12 |
| rs1431196 | 18 | 50832102 | G/A | -0.01 | 1.92E-03 | 1.89E-12 | -0.04 | 5.25E-03 | 2.50E-16 | -0.01 | 2.77E-03 | 4.26E-05 | -0.03 | 4.86E-03 | 2.65E-11 |
| rs1510719*# | 4 | 140938116 | C/T | 0.02 | 1.97E-03 | 4.40E-16 | 0.05 | 5.34E-03 | 8.18E-22 | 0.01 | 2.85E-03 | 4.66E-06 | 0.04 | 4.95E-03 | 3.84E-15 |
| rs215614 | 7 | 32347335 | A/G | 0.01 | 1.95E-03 | 2.96E-09 | 0.04 | 5.37E-03 | 1.13E-14 | 0.01 | 2.87E-03 | 0.00985 | 0.03 | 4.98E-03 | 4.08E-11 |
| rs2179152 | 6 | 26325888 | C/T | 0.01 | 1.98E-03 | 1.08E-11 | 0.04 | 5.34E-03 | 6.63E-17 | 0.01 | 2.84E-03 | 0.000516 | 0.03 | 4.96E-03 | 3.19E-12 |
| rs2240326 | 3 | 50128386 | A/G | 0.01 | 1.89E-03 | 2.59E-13 | 0.06 | 5.19E-03 | 1.18E-27 | 0.00 | 2.76E-03 | 0.0917 | 0.05 | 4.81E-03 | 1.13E-22 |
| rs2396766*# | 7 | 114318071 | A/G | -0.01 | 1.91E-03 | 1.03E-10 | -0.04 | 5.20E-03 | 1.34E-15 | -0.01 | 2.76E-03 | 0.00114 | -0.03 | 4.82E-03 | 2.33E-11 |
| rs2734839*# | 11 | 113286490 | T/C | 0.01 | 1.92E-03 | 5.03E-09 | 0.04 | 5.31E-03 | 2.82E-12 | 0.01 | 2.82E-03 | 0.00131 | 0.03 | 4.93E-03 | 8.79E-09 |
| rs3766823 | 1 | 32197257 | A/G | -0.01 | 2.39E-03 | 4.96E-08 | -0.05 | 6.88E-03 | 7.26E-13 | -0.01 | 3.65E-03 | 0.0235 | -0.04 | 6.39E-03 | 7.09E-10 |
| rs3863241 | 8 | 73890335 | T/C | -0.01 | 1.89E-03 | 9.99E-11 | -0.04 | 5.19E-03 | 8.40E-16 | -0.01 | 2.77E-03 | 0.00134 | -0.03 | 4.82E-03 | 1.49E-11 |
| rs4300861 | 2 | 22549441 | T/C | -0.01 | 1.95E-03 | 4.53E-08 | -0.04 | 5.34E-03 | 5.28E-13 | -0.01 | 2.85E-03 | 0.0237 | -0.03 | 4.95E-03 | 5.43E-10 |
| rs4382592 | 9 | 134870755 | G/T | 0.01 | 2.02E-03 | 3.52E-08 | 0.04 | 5.66E-03 | 6.44E-12 | 0.01 | 2.99E-03 | 0.00646 | 0.03 | 5.25E-03 | 8.20E-09 |
| rs4713692 | 6 | 33807638 | T/C | 0.01 | 2.00E-03 | 2.91E-09 | 0.04 | 5.37E-03 | 7.81E-12 | 0.01 | 2.87E-03 | 0.000414 | 0.03 | 4.99E-03 | 3.07E-08 |
| rs4772087 | 13 | 99115041 | T/C | -0.01 | 1.94E-03 | 2.05E-08 | -0.04 | 5.36E-03 | 3.18E-13 | -0.01 | 2.83E-03 | 0.0147 | -0.03 | 4.97E-03 | 4.79E-10 |
| rs4851239 | 2 | 100489966 | T/C | 0.01 | 1.95E-03 | 5.37E-09 | 0.04 | 5.34E-03 | 1.25E-14 | 0.01 | 2.84E-03 | 0.0168 | 0.03 | 4.95E-03 | 3.24E-11 |
| rs4855845 | 3 | 49687043 | C/T | 0.01 | 2.47E-03 | 4.12E-08 | 0.06 | 6.79E-03 | 4.24E-17 | 0.00 | 3.64E-03 | 0.311 | 0.05 | 6.30E-03 | 2.35E-14 |
| rs624833 | 4 | 2881256 | G/T | 0.01 | 2.04E-03 | 8.21E-11 | 0.03 | 5.63E-03 | 9.37E-09 | 0.02 | 3.01E-03 | 8.61E-09 | 0.02 | 5.24E-03 | 7.66E-05 |
| rs6542923 | 2 | 100892516 | T/C | 0.01 | 2.04E-03 | 2.62E-08 | 0.04 | 5.57E-03 | 1.00E-12 | 0.01 | 2.94E-03 | 0.0118 | 0.03 | 5.16E-03 | 1.36E-09 |
| rs6711584 | 2 | 104421692 | A/G | -0.01 | 1.91E-03 | 3.79E-09 | -0.04 | 5.23E-03 | 8.80E-15 | -0.01 | 2.77E-03 | 0.0145 | -0.03 | 4.84E-03 | 2.66E-11 |
| rs6722661 | 2 | 100806588 | A/G | 0.01 | 1.99E-03 | 1.71E-08 | 0.04 | 5.39E-03 | 7.49E-14 | 0.01 | 2.88E-03 | 0.0227 | 0.03 | 5.00E-03 | 1.15E-10 |
| rs731163 | 10 | 106541900 | A/G | -0.01 | 2.26E-03 | 2.39E-08 | -0.04 | 6.26E-03 | 7.84E-13 | -0.01 | 3.31E-03 | 0.0116 | -0.04 | 5.80E-03 | 1.13E-09 |
| rs761777 | 10 | 134938075 | G/A | -0.01 | 2.20E-03 | 1.35E-08 | -0.04 | 5.96E-03 | 2.67E-13 | -0.01 | 3.21E-03 | 0.0118 | -0.03 | 5.54E-03 | 4.71E-10 |
| rs7685686 | 4 | 3207142 | G/A | 0.01 | 1.90E-03 | 1.63E-12 | 0.04 | 5.25E-03 | 1.25E-13 | 0.01 | 2.79E-03 | 6.12E-07 | 0.03 | 4.89E-03 | 1.14E-08 |
| rs773109 | 12 | 56374695 | A/G | 0.02 | 2.05E-03 | 5.41E-14 | 0.05 | 5.50E-03 | 1.65E-19 | 0.01 | 2.94E-03 | 4.64E-05 | 0.04 | 5.10E-03 | 8.71E-14 |
| rs9358901 | 6 | 26024436 | T/G | 0.01 | 2.01E-03 | 6.86E-10 | 0.04 | 5.62E-03 | 4.93E-14 | 0.01 | 2.97E-03 | 0.00135 | 0.03 | 5.21E-03 | 3.68E-10 |
| rs9372625 | 6 | 98344031 | A/G | 0.01 | 1.96E-03 | 7.93E-10 | 0.05 | 5.35E-03 | 5.57E-18 | 0.01 | 2.85E-03 | 0.0505 | 0.04 | 4.95E-03 | 2.62E-14 |
| rs942065 | 14 | 94032065 | A/G | -0.01 | 1.95E-03 | 1.48E-09 | -0.04 | 5.40E-03 | 1.60E-13 | -0.01 | 2.87E-03 | 0.00166 | -0.03 | 5.01E-03 | 8.45E-10 |
| rs9540720 | 13 | 66922705 | G/A | -0.01 | 1.89E-03 | 3.88E-10 | -0.04 | 5.20E-03 | 3.26E-12 | -0.01 | 2.76E-03 | 6.52E-05 | -0.03 | 4.82E-03 | 3.01E-08 |
| rs9837341 | 3 | 49664767 | G/A | 0.02 | 2.05E-03 | 1.60E-13 | 0.05 | 5.66E-03 | 4.24E-22 | 0.01 | 3.01E-03 | 0.00171 | 0.04 | 5.26E-03 | 1.20E-16 |
| rs9941110 | 16 | 60654164 | T/C | 0.01 | 2.10E-03 | 1.51E-09 | 0.04 | 5.81E-03 | 1.44E-13 | 0.01 | 3.11E-03 | 0.00177 | 0.03 | 5.39E-03 | 7.48E-10 |

1. X represents the exposure factors, which are five smoking behavior. Those marked with '*' indicate 'risk SNPs' at hess loci significantly associated with GERD within smoking behavior. '#' signifies exclusion of multi-functional SNPs in LD (LD r^2^ ≥ 0.02) with any previously reported significant SNP from single-trait GWAS of SI, NS, ES, ASI and GERD.

2. ALT/ REF: Effect allele/ Other allele; MTAG: Multi-trait analysis of GWAS; CPASSOC: Cross Phenotype Association; SI: Smoking initiation; NS: Never Smoking; ES: Ever Smoking; ASI: Age of smoking initiation; GERD: Gastroesophageal reflux disease.

# Figure S1. Visualization of Genome-wide Loci Distribution for GERD


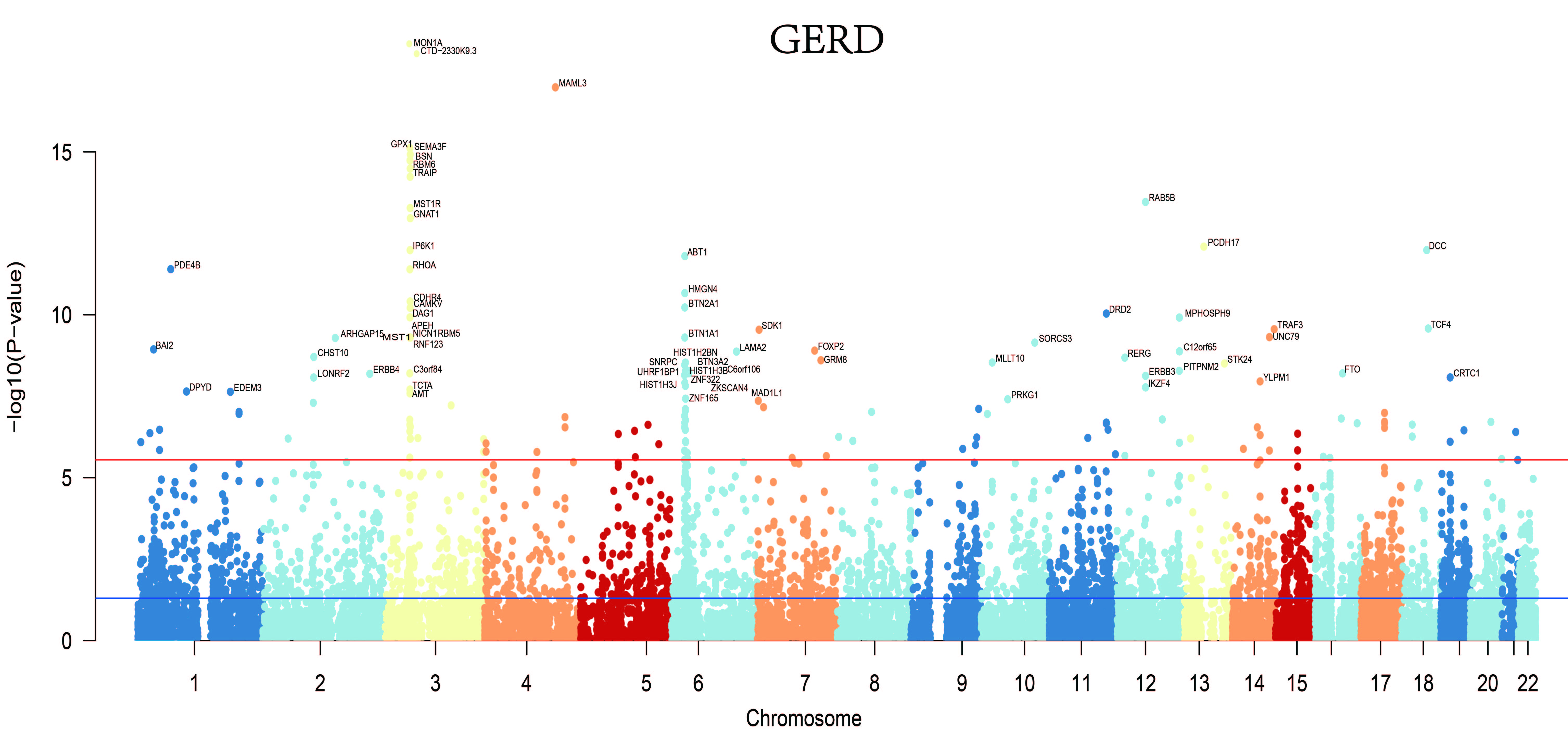


The Manhattan plot illustrates the distribution of genes within chromosomal regions for Gastroesophageal reflux disease (GERD), with genes labeled as those with P > 5E-08 on each chromosome. Additionally, blue and red solid lines represent significance thresholds of P < 0.05 and P < 0.05/ NUMSNP (number of genes in loci), respectively.

# Figure S2. Visualization of Genome-wide Loci Distribution for SI and Mapped Genes of "pleiotropic SNPs"


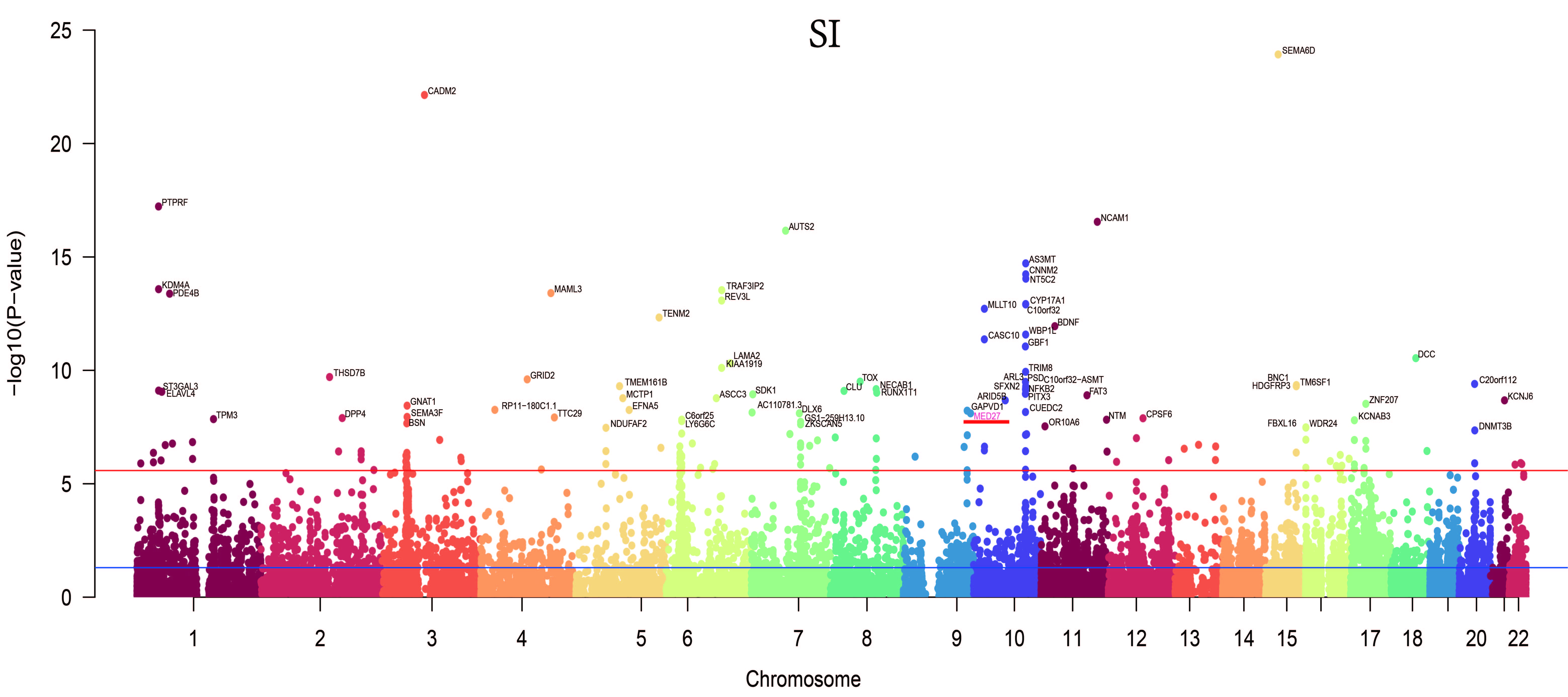


The Manhattan plot illustrates the distribution of genes within chromosomal regions for Smoking initiation (SI), with genes labeled as those with P > 5E-08 on each chromosome. Additionally, blue and red solid lines represent significance thresholds of P < 0.05 and P < 0.05/ NUMSNP (number of genes in loci), respectively. Genes highlighted in red are those mapped from "pleiotropic SNPs" identified through Heritability estimation from summary statistics (HESS) analysis.

# Figure S3. Visualization of Genome-wide Loci Distribution for NS and Mapped Genes of "pleiotropic SNPs"


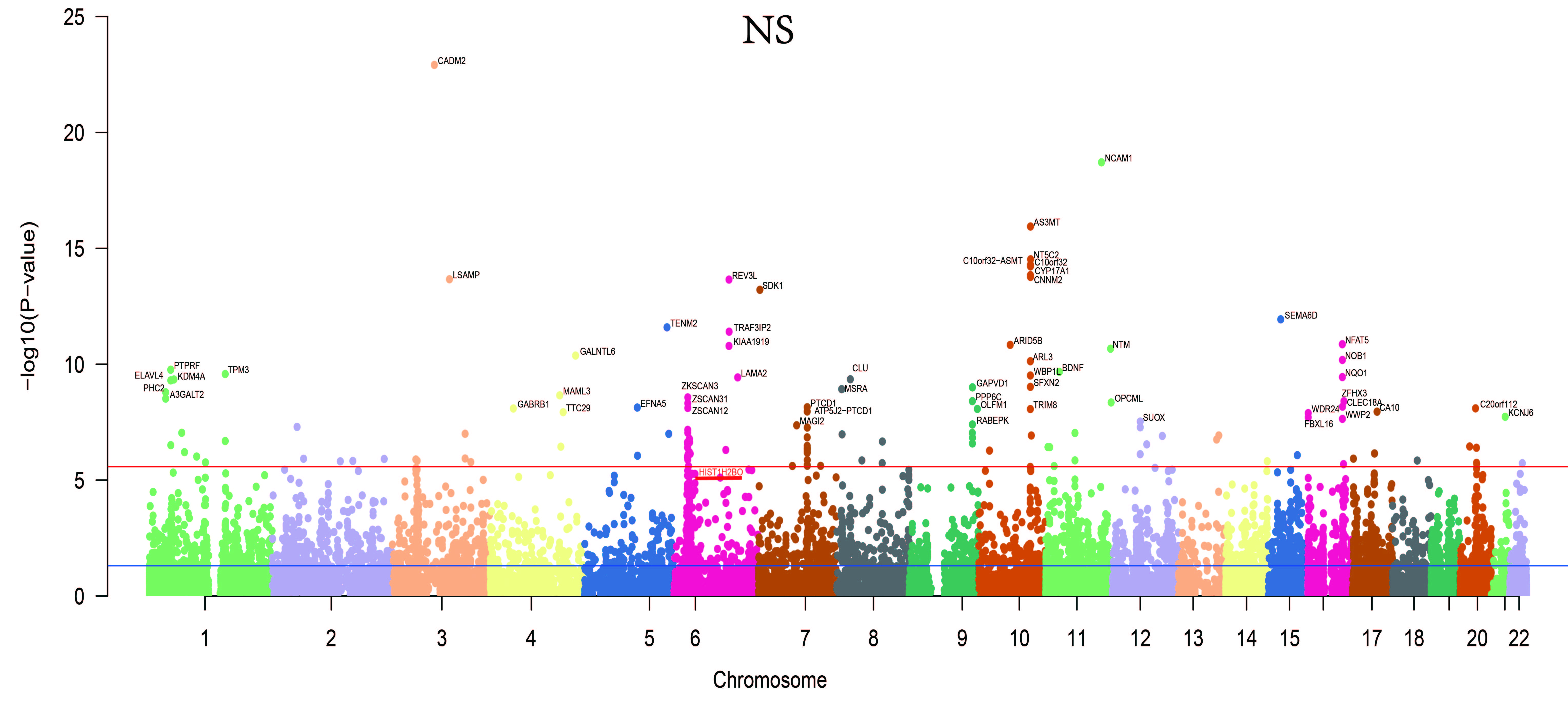


The Manhattan plot illustrates the distribution of genes within chromosomal regions for Never smoking (NS), with genes labeled as those with P > 5E-08 on each chromosome. Additionally, blue and red solid lines represent significance thresholds of P < 0.05 and P < 0.05/ NUMSNP (number of genes in loci), respectively. Genes highlighted in red are those mapped from "pleiotropic SNPs" identified through Heritability estimation from summary statistics (HESS) analysis.

# Figure S4. Visualization of Genome-wide Loci Distribution for ES and Mapped Genes of "pleiotropic SNPs"


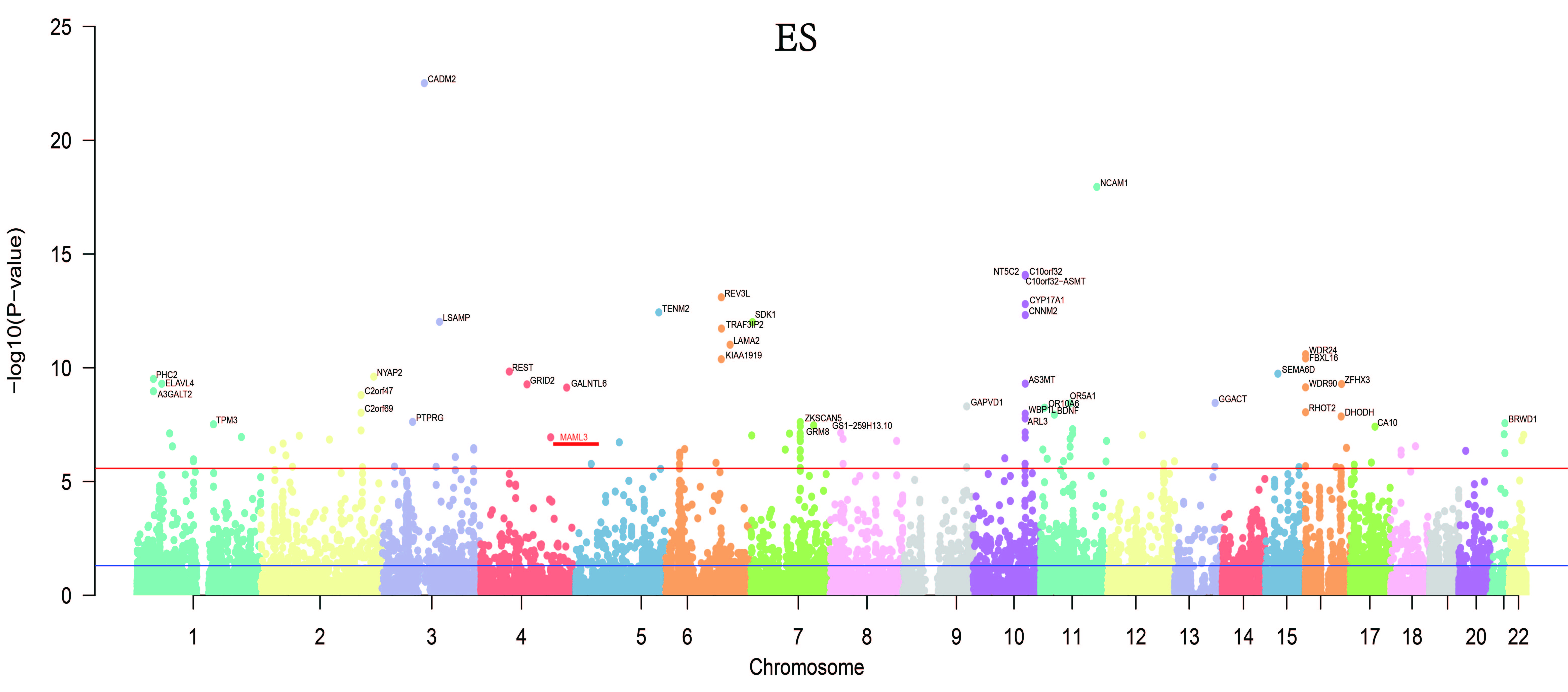


The Manhattan plot illustrates the distribution of genes within chromosomal regions for Ever smoking (ES), with genes labeled as those with P > 5E-08 on each chromosome. Additionally, blue and red solid lines represent significance thresholds of P < 0.05 and P < 0.05/ NUMSNP (number of genes in loci), respectively. Genes highlighted in red are those mapped from "pleiotropic SNPs" identified through Heritability estimation from summary statistics (HESS) analysis.

# Figure S5. Visualization of Genome-wide Loci Distribution for CPD


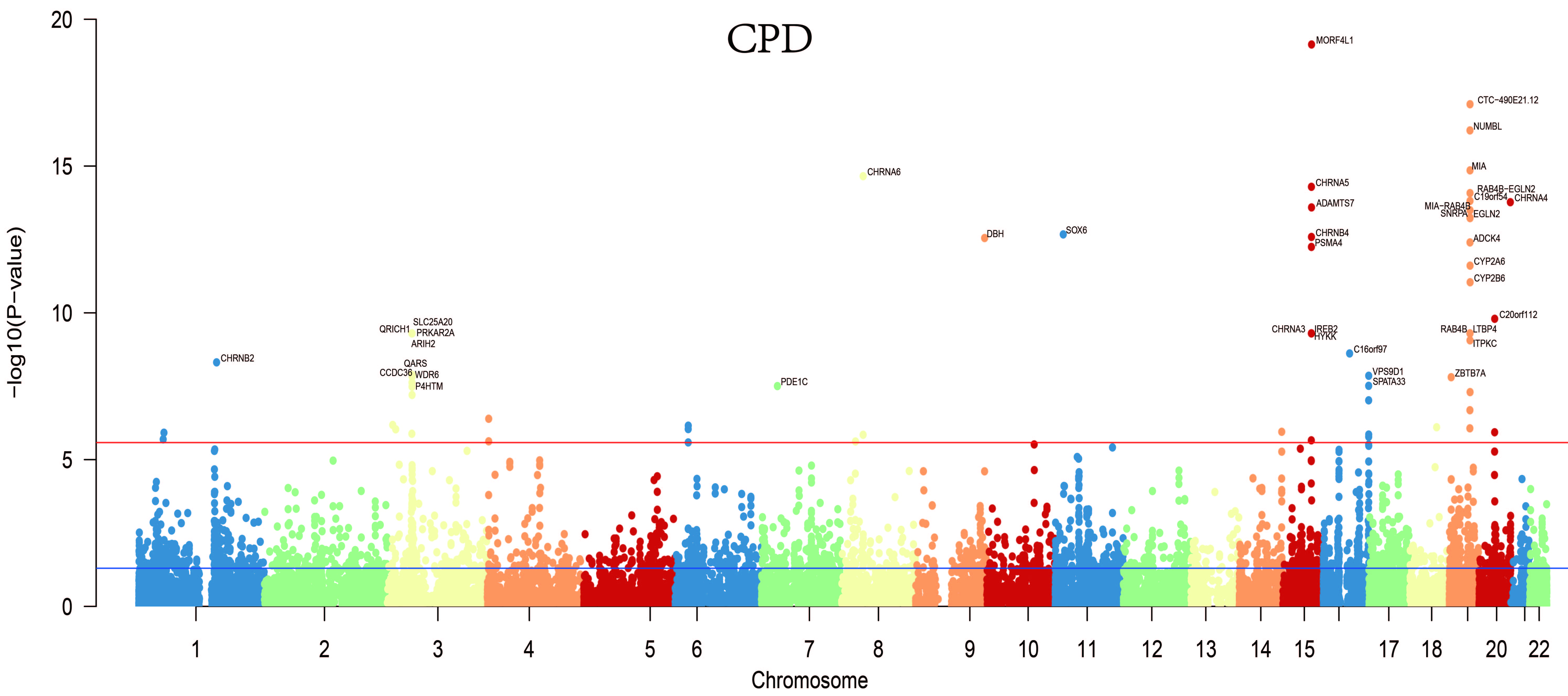


The Manhattan plot illustrates the distribution of genes within chromosomal regions for Cigarettes smoked per day (CPD), with genes labeled as those with P > 5E-08 on each chromosome. Additionally, blue and red solid lines represent significance thresholds of P < 0.05 and P < 0.05/ NUMSNP (number of genes in loci).

# Figure S6. Visualization of Genome-wide Loci Distribution for ASI and Mapped Genes of "pleiotropic SNPs"


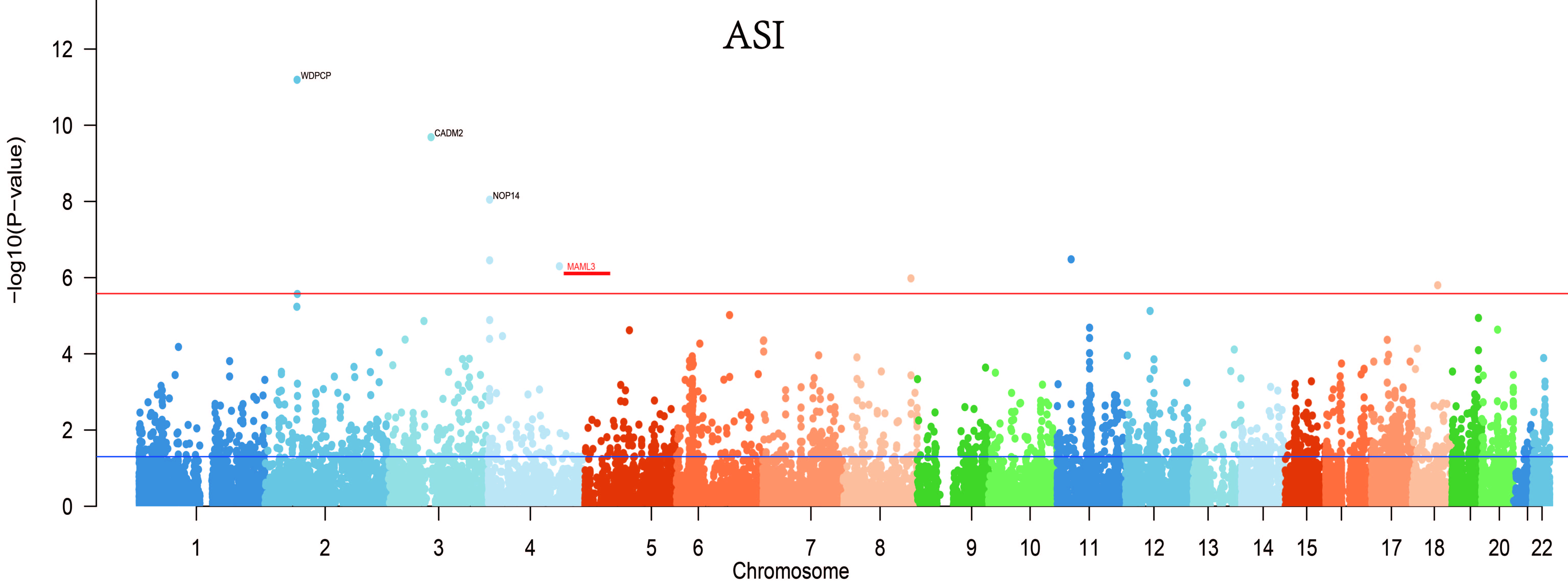


The Manhattan plot illustrates the distribution of genes within chromosomal regions for Age of smoking initiation (ASI), with genes labeled as those with P > 5E-08 on each chromosome. Additionally, blue and red solid lines represent significance thresholds of P < 0.05 and P < 0.05/ NUMSNP (number of genes in loci). Genes highlighted in red are those mapped from "pleiotropic SNPs" identified through Heritability estimation from summary statistics (HESS) analysis.

# Table S8: Tissue specific enrichment for five smoking behavior and GERD

| **VARIABLE** | **NGENES** | **BETA** | **BETA_STD** | **SE** | **P** |
| --- | --- | --- | --- | --- | --- |
| **GERD** |  |  |  |  |  |
| Brain_Cerebellum* | 16172 | 0.058865 | 0.11588 | 0.0078974 | 4.79E-14 |
| Brain_Cerebellar_Hemisphere* | 16172 | 0.056683 | 0.11289 | 0.0076429 | 6.34E-14 |
| Brain_Frontal_Cortex_BA9* | 16172 | 0.049967 | 0.09107 | 0.0086652 | 4.13E-09 |
| Brain_Cortex* | 16172 | 0.048612 | 0.087653 | 0.0090069 | 3.44E-08 |
| Brain_Anterior_cingulate_cortex_BA24* | 16172 | 0.046218 | 0.080711 | 0.0091241 | 2.06E-07 |
| Brain_Nucleus_accumbens_basal_ganglia* | 16172 | 0.0394 | 0.067534 | 0.0096113 | 2.08E-05 |
| Brain_Hypothalamus* | 16172 | 0.041332 | 0.070206 | 0.010259 | 2.82E-05 |
| Brain_Hippocampus* | 16172 | 0.036219 | 0.060547 | 0.010076 | 1.63E-04 |
| Brain_Amygdala* | 16172 | 0.035494 | 0.059733 | 0.009922 | 1.74E-04 |
| Brain_Caudate_basal_ganglia* | 16172 | 0.03305 | 0.056399 | 0.0099465 | 4.47E-04 |
| Brain_Putamen_basal_ganglia | 16172 | 0.029776 | 0.05007 | 0.010028 | 1.50E-03 |
| Pituitary | 16172 | 0.029128 | 0.053565 | 0.011299 | 4.97E-03 |
| Brain_Substantia_nigra | 16172 | 0.019114 | 0.032501 | 0.010804 | 0.038444 |
| Brain_Spinal_cord_cervical_c-1 | 16172 | 0.014784 | 0.026273 | 0.010675 | 0.083057 |
| Testis | 16172 | 0.0037361 | 0.0063653 | 0.0070336 | 0.29765 |
| Colon_Sigmoid | 16172 | 0.0077983 | 0.015096 | 0.01574 | 0.31015 |
| Cervix_Endocervix | 16172 | 0.0033205 | 0.0064945 | 0.014588 | 0.40998 |
| Cells_Cultured_fibroblasts | 16172 | 0.00095722 | 0.0020381 | 0.008258 | 0.45386 |
| Uterus | 16172 | 0.0011298 | 0.0022783 | 0.013677 | 0.46708 |
| Cervix_Ectocervix | 16172 | -0.00097266 | -0.001885 | 0.015288 | 0.52536 |
| Muscle_Skeletal | 16172 | -0.0010371 | -0.0019488 | 0.0087743 | 0.54704 |
| Artery_Tibial | 16172 | -0.0033204 | -0.0067802 | 0.012536 | 0.60444 |
| Nerve_Tibial | 16172 | -0.0046585 | -0.0092726 | 0.012717 | 0.64294 |
| Esophagus_Gastroesophageal_Junction | 16172 | -0.010016 | -0.019514 | 0.016137 | 0.73259 |
| Heart_Atrial_Appendage | 16172 | -0.007719 | -0.013707 | 0.011958 | 0.74069 |
| Artery_Aorta | 16172 | -0.010005 | -0.020238 | 0.012872 | 0.78149 |
| Adrenal_Gland | 16172 | -0.0099372 | -0.018992 | 0.012687 | 0.78325 |
| Cells_EBV-transformed_lymphocytes | 16172 | -0.0060506 | -0.013153 | 0.0061353 | 0.83797 |
| Esophagus_Muscularis | 16172 | -0.016978 | -0.033156 | 0.015632 | 0.86128 |
| Ovary | 16172 | -0.015141 | -0.030399 | 0.01211 | 0.89439 |
| Prostate | 16172 | -0.024462 | -0.045815 | 0.015155 | 0.94673 |
| Artery_Coronary | 16172 | -0.024231 | -0.047749 | 0.014908 | 0.94794 |
| Whole_Blood | 16172 | -0.011902 | -0.021342 | 0.007073 | 0.95377 |
| Bladder | 16172 | -0.028039 | -0.054037 | 0.016427 | 0.95607 |
| Heart_Left_Ventricle | 16172 | -0.020989 | -0.034641 | 0.011433 | 0.96679 |
| Vagina | 16172 | -0.029712 | -0.056143 | 0.013967 | 0.98329 |
| Fallopian_Tube | 16172 | -0.033342 | -0.063907 | 0.014848 | 0.98763 |
| Liver | 16172 | -0.018927 | -0.033783 | 0.0079186 | 0.99158 |
| Skin_Sun_Exposed_Lower_leg | 16172 | -0.025278 | -0.048532 | 0.0098368 | 0.99491 |
| Colon_Transverse | 16172 | -0.039386 | -0.071527 | 0.014627 | 0.99645 |
| Adipose_Subcutaneous | 16172 | -0.037269 | -0.073675 | 0.013528 | 0.99706 |
| Skin_Not_Sun_Exposed_Suprapubic | 16172 | -0.027127 | -0.051783 | 0.0098058 | 0.99716 |
| Pancreas | 16172 | -0.029686 | -0.049097 | 0.010528 | 0.99759 |
| Stomach | 16172 | -0.044963 | -0.080743 | 0.015128 | 0.99852 |
| Spleen | 16172 | -0.027576 | -0.054085 | 0.0092696 | 0.99853 |
| Esophagus_Mucosa | 16172 | -0.031524 | -0.060292 | 0.00968 | 0.99943 |
| Minor_Salivary_Gland | 16172 | -0.040726 | -0.074886 | 0.012127 | 0.99961 |
| Breast_Mammary_Tissue | 16172 | -0.053514 | -0.10178 | 0.015808 | 0.99964 |
| Lung | 16172 | -0.041946 | -0.08014 | 0.0116 | 0.99985 |
| Small_Intestine_Terminal_Ileum | 16172 | -0.047841 | -0.086851 | 0.012023 | 0.99997 |
| Adipose_Visceral_Omentum | 16172 | -0.05767 | -0.1109 | 0.014001 | 0.99998 |
| Kidney_Medulla | 16172 | -0.050222 | -0.091708 | 0.011685 | 0.99999 |
| Thyroid | 16172 | -0.051719 | -0.10128 | 0.012176 | 0.99999 |
| Kidney_Cortex | 16172 | -0.053588 | -0.093018 | 0.011292 | 1 |
| **SI** |  |  |  |  |  |
| Brain_Cerebellar_Hemisphere* | 17305 | 0.069749 | 0.13993 | 0.007328 | 9.99E-22 |
| Brain_Cerebellum* | 17305 | 0.071298 | 0.1415 | 0.0075558 | 2.18E-21 |
| Brain_Frontal_Cortex_BA9* | 17305 | 0.067381 | 0.12411 | 0.0082984 | 2.50E-16 |
| Brain_Cortex* | 17305 | 0.068098 | 0.12415 | 0.0086098 | 1.37E-15 |
| Brain_Nucleus_accumbens_basal_ganglia* | 17305 | 0.067318 | 0.11676 | 0.0091576 | 1.03E-13 |
| Brain_Anterior_cingulate_cortex_BA24* | 17305 | 0.063337 | 0.11185 | 0.0087019 | 1.76E-13 |
| Brain_Caudate_basal_ganglia* | 17305 | 0.063972 | 0.11047 | 0.009481 | 7.78E-12 |
| Brain_Hypothalamus* | 17305 | 0.065343 | 0.11243 | 0.0097846 | 1.25E-11 |
| Brain_Putamen_basal_ganglia* | 17305 | 0.059238 | 0.10078 | 0.0095554 | 2.90E-10 |
| Brain_Amygdala* | 17305 | 0.057467 | 0.097877 | 0.0094344 | 5.73E-10 |
| Brain_Hippocampus* | 17305 | 0.057571 | 0.097468 | 0.0095993 | 1.02E-09 |
| Brain_Substantia_nigra* | 17305 | 0.043345 | 0.074594 | 0.010333 | 1.37E-05 |
| Pituitary* | 17305 | 0.040092 | 0.074708 | 0.010811 | 1.05E-04 |
| Brain_Spinal_cord_cervical_c-1* | 17305 | 0.032546 | 0.058483 | 0.0104 | 8.77E-04 |
| Muscle_Skeletal | 17305 | 0.0097607 | 0.018464 | 0.0083705 | 0.1218 |
| Ovary | 17305 | 0.0065838 | 0.013345 | 0.011585 | 0.28492 |
| Colon_Sigmoid | 17305 | 0.00032874 | 0.00064299 | 0.01509 | 0.49131 |
| Testis | 17305 | 8.58E-05 | 0.00014814 | 0.0067198 | 0.4949 |
| Cells_EBV-transformed_lymphocytes | 17305 | -0.0014086 | -0.0030733 | 0.0059151 | 0.59411 |
| Cells_Cultured_fibroblasts | 17305 | -0.0092 | -0.019709 | 0.0078402 | 0.87968 |
| Uterus | 17305 | -0.018281 | -0.037237 | 0.013129 | 0.91808 |
| Esophagus_Muscularis | 17305 | -0.024222 | -0.047771 | 0.014981 | 0.94703 |
| Nerve_Tibial | 17305 | -0.022945 | -0.046171 | 0.012183 | 0.97016 |
| Heart_Left_Ventricle | 17305 | -0.020809 | -0.034739 | 0.010948 | 0.97132 |
| Adrenal_Gland | 17305 | -0.02327 | -0.044909 | 0.012055 | 0.97321 |
| Esophagus_Gastroesophageal_Junction | 17305 | -0.0303 | -0.059639 | 0.01546 | 0.97499 |
| Cervix_Endocervix | 17305 | -0.029708 | -0.058794 | 0.013952 | 0.98338 |
| Cervix_Ectocervix | 17305 | -0.032156 | -0.063012 | 0.014653 | 0.9859 |
| Pancreas | 17305 | -0.023401 | -0.039138 | 0.010085 | 0.98983 |
| Whole_Blood | 17305 | -0.016958 | -0.030652 | 0.0068059 | 0.99364 |
| Artery_Tibial | 17305 | -0.031026 | -0.063871 | 0.012133 | 0.99472 |
| Heart_Atrial_Appendage | 17305 | -0.02985 | -0.053593 | 0.011449 | 0.99543 |
| Skin_Sun_Exposed_Lower_leg | 17305 | -0.027947 | -0.054215 | 0.0094015 | 0.99852 |
| Liver | 17305 | -0.022853 | -0.04115 | 0.0075849 | 0.9987 |
| Skin_Not_Sun_Exposed_Suprapubic | 17305 | -0.030061 | -0.05796 | 0.0093703 | 0.99933 |
| Thyroid | 17305 | -0.042818 | -0.084773 | 0.011657 | 0.99988 |
| Fallopian_Tube | 17305 | -0.053244 | -0.10326 | 0.014212 | 0.99991 |
| Colon_Transverse | 17305 | -0.05412 | -0.099481 | 0.013877 | 0.99995 |
| Spleen | 17305 | -0.035002 | -0.069361 | 0.0089287 | 0.99996 |
| Prostate | 17305 | -0.059579 | -0.113 | 0.014485 | 0.99998 |
| Vagina | 17305 | -0.054754 | -0.10468 | 0.013399 | 0.99998 |
| Esophagus_Mucosa | 17305 | -0.039909 | -0.077135 | 0.0092216 | 0.99999 |
| Adipose_Subcutaneous | 17305 | -0.057739 | -0.11521 | 0.012995 | 1 |
| Adipose_Visceral_Omentum | 17305 | -0.077538 | -0.15058 | 0.013505 | 1 |
| Artery_Aorta | 17305 | -0.062069 | -0.12665 | 0.012372 | 1 |
| Artery_Coronary | 17305 | -0.077359 | -0.15397 | 0.014347 | 1 |
| Bladder | 17305 | -0.071631 | -0.13957 | 0.015801 | 1 |
| Breast_Mammary_Tissue | 17305 | -0.086354 | -0.16602 | 0.015306 | 1 |
| Kidney_Cortex | 17305 | -0.054768 | -0.09623 | 0.010846 | 1 |
| Kidney_Medulla | 17305 | -0.05993 | -0.1108 | 0.011151 | 1 |
| Lung | 17305 | -0.062897 | -0.12156 | 0.011071 | 1 |
| Minor_Salivary_Gland | 17305 | -0.065106 | -0.12114 | 0.011607 | 1 |
| Small_Intestine_Terminal_Ileum | 17305 | -0.05215 | -0.095864 | 0.011537 | 1 |
| Stomach | 17305 | -6.43E-02 | -0.11678 | 0.014481 | 1 |
| **NS** |  |  |  |  |  |
| Brain_Cerebellar_Hemisphere* | 18002 | 0.052483 | 0.10558 | 0.0070005 | 3.42E-14 |
| Brain_Cerebellum* | 18002 | 0.052491 | 0.10448 | 0.0072197 | 1.87E-13 |
| Brain_Frontal_Cortex_BA9* | 18002 | 0.055287 | 0.10213 | 0.0078917 | 1.27E-12 |
| Brain_Cortex* | 18002 | 0.053365 | 0.097559 | 0.0081779 | 3.48E-11 |
| Brain_Anterior_cingulate_cortex_BA24* | 18002 | 0.049702 | 0.087997 | 0.0082697 | 9.45E-10 |
| Brain_Nucleus_accumbens_basal_ganglia* | 18002 | 0.050347 | 0.08756 | 0.0086885 | 3.48E-09 |
| Brain_Caudate_basal_ganglia* | 18002 | 0.046217 | 0.080011 | 0.0090189 | 1.51E-07 |
| Brain_Hypothalamus* | 18002 | 0.045394 | 0.078365 | 0.0092523 | 4.69E-07 |
| Brain_Putamen_basal_ganglia* | 18002 | 0.044174 | 0.075315 | 0.0090983 | 6.07E-07 |
| Brain_Amygdala* | 18002 | 0.04223 | 0.072099 | 0.0089649 | 1.25E-06 |
| Brain_Hippocampus* | 18002 | 0.040546 | 0.068824 | 0.0091352 | 4.56E-06 |
| Pituitary* | 18002 | 0.034702 | 0.064953 | 0.010253 | 3.57E-04 |
| Brain_Substantia_nigra* | 18002 | 0.031944 | 0.055117 | 0.0098375 | 5.84E-04 |
| Brain_Spinal_cord_cervical_c-1 | 18002 | 0.021514 | 0.038769 | 0.0099205 | 0.015062 |
| Ovary | 18002 | 0.011475 | 0.023301 | 0.011093 | 0.15046 |
| Uterus | 18002 | 0.0043828 | 0.0089457 | 0.01251 | 0.36304 |
| Colon_Sigmoid | 18002 | 1.27E-05 | 2.50E-05 | 0.014425 | 0.49965 |
| Testis | 18002 | -0.0011191 | -0.0019342 | 0.0063177 | 0.5703 |
| Cells_EBV-transformed_lymphocytes | 18002 | -0.0031271 | -0.0068335 | 0.0055987 | 0.71176 |
| Muscle_Skeletal | 18002 | -0.0044519 | -0.0084236 | 0.0078936 | 0.71361 |
| Esophagus_Muscularis | 18002 | -0.011649 | -0.02302 | 0.014311 | 0.79218 |
| Cervix_Endocervix | 18002 | -0.010934 | -0.021702 | 0.013249 | 0.79539 |
| Esophagus_Gastroesophageal_Junction | 18002 | -0.013541 | -0.026704 | 0.014751 | 0.82067 |
| Nerve_Tibial | 18002 | -0.012823 | -0.025874 | 0.011592 | 0.86567 |
| Thyroid | 18002 | -0.012365 | -0.024558 | 0.011129 | 0.86672 |
| Heart_Left_Ventricle | 18002 | -0.016742 | -0.027966 | 0.01038 | 0.9466 |
| Prostate | 18002 | -0.023105 | -0.043956 | 0.013789 | 0.95309 |
| Cervix_Ectocervix | 18002 | -0.025132 | -0.049383 | 0.013898 | 0.96472 |
| Heart_Atrial_Appendage | 18002 | -0.021455 | -0.038578 | 0.010965 | 0.9748 |
| Cells_Cultured_fibroblasts | 18002 | -0.014716 | -0.031574 | 0.0075039 | 0.97506 |
| Adrenal_Gland | 18002 | -0.025974 | -0.050262 | 0.011507 | 0.988 |
| Artery_Tibial | 18002 | -0.026077 | -0.053749 | 0.011541 | 0.98806 |
| Fallopian_Tube | 18002 | -0.03142 | -0.061118 | 0.013579 | 0.98966 |
| Stomach | 18002 | -0.032907 | -0.059911 | 0.013783 | 0.99151 |
| Pancreas | 18002 | -0.025223 | -0.042233 | 0.0096376 | 0.99556 |
| Liver | 18002 | -0.021637 | -0.038968 | 0.0072704 | 0.99854 |
| Spleen | 18002 | -0.025343 | -0.050352 | 0.0084864 | 0.99859 |
| Colon_Transverse | 18002 | -0.039929 | -0.073625 | 0.013306 | 0.99865 |
| Skin_Sun_Exposed_Lower_leg | 18002 | -0.027271 | -0.053058 | 0.0089231 | 0.99888 |
| Skin_Not_Sun_Exposed_Suprapubic | 18002 | -0.027581 | -0.053335 | 0.0088988 | 0.99903 |
| Bladder | 18002 | -0.048636 | -0.09504 | 0.015039 | 0.99939 |
| Whole_Blood | 18002 | -0.021532 | -0.038946 | 0.0064612 | 0.99957 |
| Artery_Coronary | 18002 | -0.046835 | -0.093389 | 0.013704 | 0.99968 |
| Minor_Salivary_Gland | 18002 | -0.038217 | -0.071309 | 0.011082 | 0.99972 |
| Kidney_Medulla | 18002 | -0.036822 | -0.068284 | 0.010623 | 0.99974 |
| Small_Intestine_Terminal_Ileum | 18002 | -0.03889 | -0.071762 | 0.011009 | 0.99979 |
| Kidney_Cortex | 18002 | -0.037209 | -0.065542 | 0.010333 | 0.99984 |
| Artery_Aorta | 18002 | -0.043594 | -0.089076 | 0.011831 | 0.99989 |
| Adipose_Subcutaneous | 18002 | -0.046318 | -0.09262 | 0.012278 | 0.99992 |
| Breast_Mammary_Tissue | 18002 | -0.056053 | -0.10806 | 0.014535 | 0.99994 |
| Vagina | 18002 | -0.048757 | -0.093521 | 0.012677 | 0.99994 |
| Adipose_Visceral_Omentum | 18002 | -0.052026 | -0.10127 | 0.012767 | 0.99998 |
| Lung | 18002 | -0.043689 | -0.084793 | 0.01054 | 0.99998 |
| Esophagus_Mucosa | 18002 | -0.037786 | -0.073224 | 0.0087476 | 0.99999 |
| **ES** |  |  |  |  |  |
| Brain_Cerebellar_Hemisphere* | 17283 | 0.039501 | 0.079244 | 0.006983 | 7.84E-09 |
| Brain_Cerebellum* | 17283 | 0.039786 | 0.078955 | 0.0071967 | 1.64E-08 |
| Brain_Frontal_Cortex_BA9* | 17283 | 0.039475 | 0.072695 | 0.0079158 | 3.10E-07 |
| Brain_Cortex* | 17283 | 0.037805 | 0.068907 | 0.0082095 | 2.08E-06 |
| Brain_Anterior_cingulate_cortex_BA24* | 17283 | 0.036476 | 0.0644 | 0.0083072 | 5.68E-06 |
| Brain_Nucleus_accumbens_basal_ganglia* | 17283 | 0.036394 | 0.063113 | 0.0087342 | 1.55E-05 |
| Brain_Hypothalamus* | 17283 | 0.035402 | 0.0609 | 0.009317 | 7.27E-05 |
| Brain_Caudate_basal_ganglia* | 17283 | 0.032158 | 0.05552 | 0.0090368 | 1.87E-04 |
| Brain_Amygdala* | 17283 | 0.030327 | 0.051642 | 0.0090186 | 3.87E-04 |
| Pituitary* | 17283 | 0.033854 | 0.063074 | 0.010319 | 5.18E-04 |
| Brain_Putamen_basal_ganglia* | 17283 | 0.029853 | 0.05078 | 0.0091087 | 5.25E-04 |
| Brain_Hippocampus* | 17283 | 0.029528 | 0.049982 | 0.0091637 | 6.37E-04 |
| Brain_Substantia_nigra | 17283 | 0.022763 | 0.039167 | 0.0098606 | 0.010492 |
| Brain_Spinal_cord_cervical_c-1 | 17283 | 0.018136 | 0.032581 | 0.009897 | 0.033449 |
| Testis | 17283 | 0.0065237 | 0.011253 | 0.0064058 | 0.15425 |
| Ovary | 17283 | 0.0097816 | 0.019824 | 0.011089 | 0.18888 |
| Uterus | 17283 | 0.0060521 | 0.012325 | 0.012495 | 0.31407 |
| Adrenal_Gland | 17283 | -0.00084623 | -0.0016331 | 0.011479 | 0.52938 |
| Muscle_Skeletal | 17283 | -0.0007566 | -0.0014313 | 0.0079445 | 0.53794 |
| Cells_EBV-transformed_lymphocytes | 17283 | -0.0024762 | -0.005403 | 0.0056135 | 0.67043 |
| Nerve_Tibial | 17283 | -0.0053513 | -0.010764 | 0.011654 | 0.67694 |
| Colon_Sigmoid | 17283 | -0.0072797 | -0.014235 | 0.014437 | 0.69295 |
| Cervix_Endocervix | 17283 | -0.0090535 | -0.017911 | 0.013279 | 0.75231 |
| Cervix_Ectocervix | 17283 | -0.0097651 | -0.019129 | 0.013943 | 0.75814 |
| Esophagus_Gastroesophageal_Junction | 17283 | -0.01334 | -0.026252 | 0.014749 | 0.81712 |
| Esophagus_Muscularis | 17283 | -0.013049 | -0.02573 | 0.014283 | 0.81952 |
| Artery_Tibial | 17283 | -0.010565 | -0.021746 | 0.011503 | 0.82081 |
| Thyroid | 17283 | -0.010849 | -0.021475 | 0.011128 | 0.8352 |
| Heart_Atrial_Appendage | 17283 | -0.010815 | -0.019415 | 0.010882 | 0.83985 |
| Cells_Cultured_fibroblasts | 17283 | -0.007655 | -0.016398 | 0.0074726 | 0.84717 |
| Heart_Left_Ventricle | 17283 | -0.010909 | -0.018212 | 0.010387 | 0.8532 |
| Pancreas | 17283 | -0.010067 | -0.016839 | 0.0095448 | 0.85422 |
| Liver | 17283 | -0.0087817 | -0.015813 | 0.007235 | 0.88757 |
| Fallopian_Tube | 17283 | -0.026064 | -0.050526 | 0.013619 | 0.97217 |
| Colon_Transverse | 17283 | -0.025631 | -0.047101 | 0.013219 | 0.97374 |
| Prostate | 17283 | -0.031325 | -0.059386 | 0.013783 | 0.98847 |
| Minor_Salivary_Gland | 17283 | -0.026026 | -0.048409 | 0.011046 | 0.99076 |
| Artery_Aorta | 17283 | -0.02826 | -0.057651 | 0.011751 | 0.9919 |
| Vagina | 17283 | -0.030517 | -0.058321 | 0.01266 | 0.99203 |
| Kidney_Cortex | 17283 | -0.02591 | -0.045513 | 0.010296 | 0.99407 |
| Skin_Sun_Exposed_Lower_leg | 17283 | -0.023126 | -0.044848 | 0.0088798 | 0.99539 |
| Skin_Not_Sun_Exposed_Suprapubic | 17283 | -0.024277 | -0.046795 | 0.008858 | 0.99693 |
| Esophagus_Mucosa | 17283 | -0.024072 | -0.046516 | 0.008732 | 0.99708 |
| Bladder | 17283 | -0.041672 | -0.08117 | 0.014999 | 0.99726 |
| Artery_Coronary | 17283 | -0.037957 | -0.075524 | 0.013652 | 0.99728 |
| Adipose_Subcutaneous | 17283 | -0.034833 | -0.069484 | 0.012378 | 0.99755 |
| Kidney_Medulla | 17283 | -0.031042 | -0.057374 | 0.010663 | 0.9982 |
| Whole_Blood | 17283 | -0.01976 | -0.035726 | 0.006457 | 0.99889 |
| Small_Intestine_Terminal_Ileum | 17283 | -0.034661 | -0.063697 | 0.010955 | 0.99922 |
| Spleen | 17283 | -0.028085 | -0.055642 | 0.0084893 | 0.99953 |
| Breast_Mammary_Tissue | 17283 | -0.049514 | -0.095156 | 0.014554 | 0.99966 |
| Stomach | 17283 | -0.049682 | -0.090228 | 0.013714 | 0.99985 |
| Adipose_Visceral_Omentum | 17283 | -0.048291 | -0.093751 | 0.012839 | 0.99992 |
| Lung | 17283 | -0.04947 | -0.095569 | 0.010582 | 1 |
| **CPD** |  |  |  |  |  |
| Brain_Cerebellar_Hemisphere* | 17301 | 0.045956 | 0.092188 | 0.0067451 | 4.94E-12 |
| Brain_Cerebellum* | 17301 | 0.047025 | 0.093322 | 0.0069558 | 7.10E-12 |
| Brain_Cortex* | 17301 | 0.045697 | 0.083302 | 0.0079152 | 3.96E-09 |
| Brain_Frontal_Cortex_BA9* | 17301 | 0.043769 | 0.080609 | 0.0076259 | 4.83E-09 |
| Brain_Anterior_cingulate_cortex_BA24* | 17301 | 0.040367 | 0.071276 | 0.0080055 | 2.32E-07 |
| Brain_Hypothalamus* | 17301 | 0.043217 | 0.074346 | 0.0089954 | 7.83E-07 |
| Brain_Nucleus_accumbens_basal_ganglia* | 17301 | 0.03984 | 0.069091 | 0.0084334 | 1.17E-06 |
| Brain_Caudate_basal_ganglia* | 17301 | 0.035738 | 0.061704 | 0.0087341 | 2.15E-05 |
| Brain_Hippocampus* | 17301 | 0.033971 | 0.057507 | 0.0088276 | 5.97E-05 |
| Brain_Amygdala* | 17301 | 0.031485 | 0.053619 | 0.0086898 | 1.46E-04 |
| Brain_Putamen_basal_ganglia* | 17301 | 0.029762 | 0.050627 | 0.0087965 | 3.59E-04 |
| Pituitary* | 17301 | 0.033294 | 0.062033 | 0.0099502 | 4.11E-04 |
| Brain_Substantia_nigra | 17301 | 0.024392 | 0.041971 | 0.0095078 | 5.16E-03 |
| Brain_Spinal_cord_cervical_c-1 | 17301 | 0.017635 | 0.031686 | 0.0095449 | 3.23E-02 |
| Muscle_Skeletal | 17301 | 0.0074747 | 0.014139 | 0.0076477 | 0.1642 |
| Colon_Sigmoid | 17301 | 0.007424 | 0.01452 | 0.013912 | 0.2968 |
| Testis | 17301 | 9.49E-04 | 0.0016382 | 0.0061747 | 0.43891 |
| Pancreas | 17301 | -0.0009564 | -0.0015994 | 0.0092593 | 0.54113 |
| Esophagus_Muscularis | 17301 | -0.0040787 | -0.0080436 | 0.013796 | 0.61625 |
| Adrenal_Gland | 17301 | -0.0038458 | -0.007422 | 0.011121 | 0.63526 |
| Nerve_Tibial | 17301 | -0.0040528 | -0.0081543 | 0.011207 | 0.64119 |
| Heart_Left_Ventricle | 17301 | -0.0037705 | -0.0062943 | 0.010081 | 0.64581 |
| Cells_Cultured_fibroblasts | 17301 | -0.0027883 | -0.0059731 | 0.0071668 | 0.65138 |
| Esophagus_Gastroesophageal_Junction | 17301 | -0.0067919 | -0.013368 | 0.014236 | 0.68335 |
| Liver | 17301 | -0.0038733 | -0.0069743 | 0.0069985 | 0.71002 |
| Heart_Atrial_Appendage | 17301 | -0.0065165 | -0.011699 | 0.010539 | 0.73182 |
| Ovary | 17301 | -0.0069928 | -0.014172 | 0.010716 | 0.74297 |
| Whole_Blood | 17301 | -0.007281 | -0.013161 | 0.0062072 | 0.87959 |
| Cells_EBV-transformed_lymphocytes | 17301 | -0.0087706 | -0.019135 | 0.0054094 | 0.94752 |
| Skin_Not_Sun_Exposed_Suprapubic | 17301 | -0.014666 | -0.028275 | 0.0086088 | 0.95576 |
| Artery_Tibial | 17301 | -0.019434 | -0.040003 | 0.011105 | 0.95994 |
| Skin_Sun_Exposed_Lower_leg | 17301 | -0.016424 | -0.031859 | 0.0086325 | 0.97145 |
| Thyroid | 17301 | -2.54E-02 | -0.050323 | 0.010733 | 0.99106 |
| Cervix_Ectocervix | 17301 | -0.032004 | -0.062707 | 0.013463 | 0.99127 |
| Uterus | 17301 | -0.032955 | -0.067122 | 0.012078 | 0.99682 |
| Colon_Transverse | 17301 | -0.035233 | -0.064753 | 0.012718 | 0.9972 |
| Stomach | 17301 | -0.037222 | -0.067599 | 0.013288 | 0.99745 |
| Minor_Salivary_Gland | 17301 | -0.030018 | -0.055846 | 0.010705 | 0.99747 |
| Artery_Aorta | 17301 | -0.03192 | -0.06513 | 0.011364 | 0.99751 |
| Cervix_Endocervix | 17301 | -0.036266 | -0.071764 | 0.012822 | 0.99766 |
| Kidney_Cortex | 17301 | -0.028891 | -0.050759 | 0.010005 | 0.99806 |
| Esophagus_Mucosa | 17301 | -0.024711 | -0.047756 | 0.0084697 | 0.99823 |
| Artery_Coronary | 17301 | -0.043189 | -0.085951 | 0.013139 | 0.99949 |
| Small_Intestine_Terminal_Ileum | 17301 | -0.036763 | -0.067568 | 0.01057 | 0.99975 |
| Vagina | 17301 | -0.043353 | -0.082873 | 0.012295 | 0.99979 |
| Fallopian_Tube | 17301 | -0.046755 | -0.09066 | 0.013105 | 0.99982 |
| Prostate | 17301 | -0.048796 | -0.092537 | 0.013299 | 0.99988 |
| Adipose_Subcutaneous | 17301 | -0.045001 | -0.089791 | 0.011906 | 0.99992 |
| Kidney_Medulla | 17301 | -0.039333 | -0.072712 | 0.010265 | 0.99994 |
| Bladder | 17301 | -0.057933 | -0.11287 | 0.014516 | 0.99997 |
| Adipose_Visceral_Omentum | 17301 | -0.050329 | -0.097731 | 0.012329 | 0.99998 |
| Spleen | 17301 | -0.033425 | -0.066229 | 0.0081653 | 0.99998 |
| Breast_Mammary_Tissue | 17301 | -0.063257 | -0.1216 | 0.014051 | 1 |
| Lung | 17301 | -0.045642 | -0.088203 | 0.010117 | 1 |
| **ASI** |  |  |  |  |  |
| Brain_Cerebellar_Hemisphere* | 17300 | 0.031966 | 0.064122 | 6.37E-03 | 2.62E-07 |
| Brain_Cerebellum* | 17300 | 0.032679 | 0.064849 | 0.0065658 | 3.26E-07 |
| Pituitary* | 17300 | 0.039428 | 0.073457 | 0.0093875 | 1.34E-05 |
| Brain_Frontal_Cortex_BA9 | 17300 | 0.02198 | 0.040479 | 7.20E-03 | 1.14E-03 |
| Brain_Cortex | 17300 | 0.022345 | 0.040732 | 7.48E-03 | 1.40E-03 |
| Brain_Anterior_cingulate_cortex_BA24 | 17300 | 0.019023 | 0.033589 | 7.56E-03 | 5.92E-03 |
| Brain_Hypothalamus | 17300 | 0.020611 | 0.035455 | 0.0084888 | 7.60E-03 |
| Brain_Nucleus_accumbens_basal_ganglia | 1.73E+04 | 0.017231 | 0.029882 | 0.0079596 | 0.015207 |
| Brain_Caudate_basal_ganglia | 17300 | 0.01608 | 0.027762 | 8.24E-03 | 0.025536 |
| Brain_Hippocampus | 17300 | 0.015986 | 0.027061 | 0.0083355 | 0.027574 |
| Brain_Amygdala | 17300 | 0.013883 | 0.023642 | 8.20E-03 | 0.045283 |
| Brain_Putamen_basal_ganglia | 17300 | 0.013113 | 0.022305 | 0.008303 | 0.057139 |
| Ovary | 17300 | 0.015175 | 0.030754 | 0.010107 | 0.066636 |
| Brain_Spinal_cord_cervical_c-1 | 17300 | 0.012511 | 0.022477 | 0.009008 | 0.082449 |
| Adrenal_Gland | 17300 | 0.010531 | 0.020323 | 1.05E-02 | 0.15731 |
| Brain_Substantia_nigra | 17300 | 0.0086425 | 0.014871 | 0.0089694 | 0.16764 |
| Liver | 17300 | 0.0030367 | 0.0054678 | 0.0066135 | 0.32306 |
| Spleen | 17300 | 0.0033177 | 0.0065734 | 0.0077161 | 0.33361 |
| Muscle_Skeletal | 17300 | 0.00067684 | 0.0012803 | 0.0072163 | 0.46264 |
| Cells_Cultured_fibroblasts | 17300 | -0.0013552 | -0.0029029 | 0.0067756 | 0.57926 |
| Testis | 17300 | -0.0018787 | -0.0032423 | 0.0058454 | 0.62604 |
| Whole_Blood | 17300 | -0.0020751 | -0.0037509 | 0.005878 | 0.63796 |
| Cells_EBV-transformed_lymphocytes | 17300 | -0.0018077 | -0.0039438 | 0.0050987 | 0.63853 |
| Kidney_Cortex | 17300 | -0.0040812 | -0.00717 | 0.009433 | 0.66736 |
| Kidney_Medulla | 17300 | -0.0047464 | -0.008774 | 0.0096863 | 0.68793 |
| Thyroid | 17300 | -0.0056634 | -0.011211 | 0.010183 | 0.71095 |
| Heart_Atrial_Appendage | 17300 | -0.0057003 | -0.010233 | 0.0099569 | 0.7165 |
| Uterus | 17300 | -0.0077027 | -0.015688 | 0.011451 | 0.74942 |
| Cervix_Ectocervix | 17300 | -0.011891 | -0.023298 | 0.012741 | 0.82466 |
| Heart_Left_Ventricle | 1.73E+04 | -0.010396 | -0.017355 | 0.0095193 | 0.86261 |
| Colon_Sigmoid | 17300 | -0.015166 | -0.02966 | 0.013131 | 0.87595 |
| Skin_Not_Sun_Exposed_Suprapubic | 17300 | -0.0095041 | -0.018323 | 0.0081286 | 0.87883 |
| Cervix_Endocervix | 17300 | -0.014796 | -0.029277 | 0.012113 | 0.88904 |
| Pancreas | 17300 | -0.011922 | -0.019937 | 0.0087636 | 0.91313 |
| Fallopian_Tube | 17300 | -0.017408 | -0.033753 | 0.012422 | 0.91945 |
| Skin_Sun_Exposed_Lower_leg | 17300 | -0.012187 | -0.023638 | 0.0081506 | 0.93256 |
| Esophagus_Muscularis | 17300 | -0.020559 | -0.040544 | 0.013024 | 0.94277 |
| Esophagus_Gastroesophageal_Junction | 17300 | -0.021247 | -0.041817 | 0.013432 | 0.94314 |
| Esophagus_Mucosa | 17300 | -0.013178 | -0.025467 | 0.0079774 | 0.95071 |
| Nerve_Tibial | 17300 | -0.017944 | -0.036102 | 0.010572 | 0.95517 |
| Prostate | 17300 | -0.022047 | -0.041808 | 0.012564 | 0.96034 |
| Artery_Tibial | 17300 | -0.020439 | -0.042071 | 1.05E-02 | 0.97456 |
| Stomach | 17300 | -0.024781 | -0.045005 | 0.012558 | 0.97576 |
| Colon_Transverse | 17300 | -0.027605 | -0.050732 | 0.012021 | 0.98916 |
| Adipose_Visceral_Omentum | 17300 | -0.027468 | -0.053338 | 1.16E-02 | 0.99083 |
| Artery_Aorta | 17300 | -0.026176 | -0.053407 | 1.07E-02 | 0.99267 |
| Lung | 17300 | -0.024133 | -0.046635 | 0.0095974 | 0.99404 |
| Artery_Coronary | 17300 | -0.031613 | -0.06291 | 1.24E-02 | 0.99459 |
| Small_Intestine_Terminal_Ileum | 17300 | -0.025763 | -0.047349 | 0.009977 | 0.99509 |
| Vagina | 17300 | -0.031176 | -0.059593 | 1.16E-02 | 0.99634 |
| Adipose_Subcutaneous | 17300 | -0.030195 | -0.060246 | 1.12E-02 | 0.99637 |
| Bladder | 17300 | -0.03961 | -0.077168 | 1.37E-02 | 0.99807 |
| Minor_Salivary_Gland | 17300 | -0.03514 | -0.065374 | 0.010088 | 0.99975 |
| Breast_Mammary_Tissue | 17300 | -0.052352 | -0.10064 | 0.013281 | 0.99996 |

1. Sites enriched in specific tissues with significant differences after Bonferroni correction are marked with "*".

2. SI: Smoking initiation; NS: Never Smoking; CPD: Cigarettes smoked per day; ES: Ever Smoking; ASI: Age of smoking initiation; GERD: Gastroesophageal reflux disease.

# Figure S7: Visualization of tissue-specific enrichment of genetic heritability of GERD's SNP


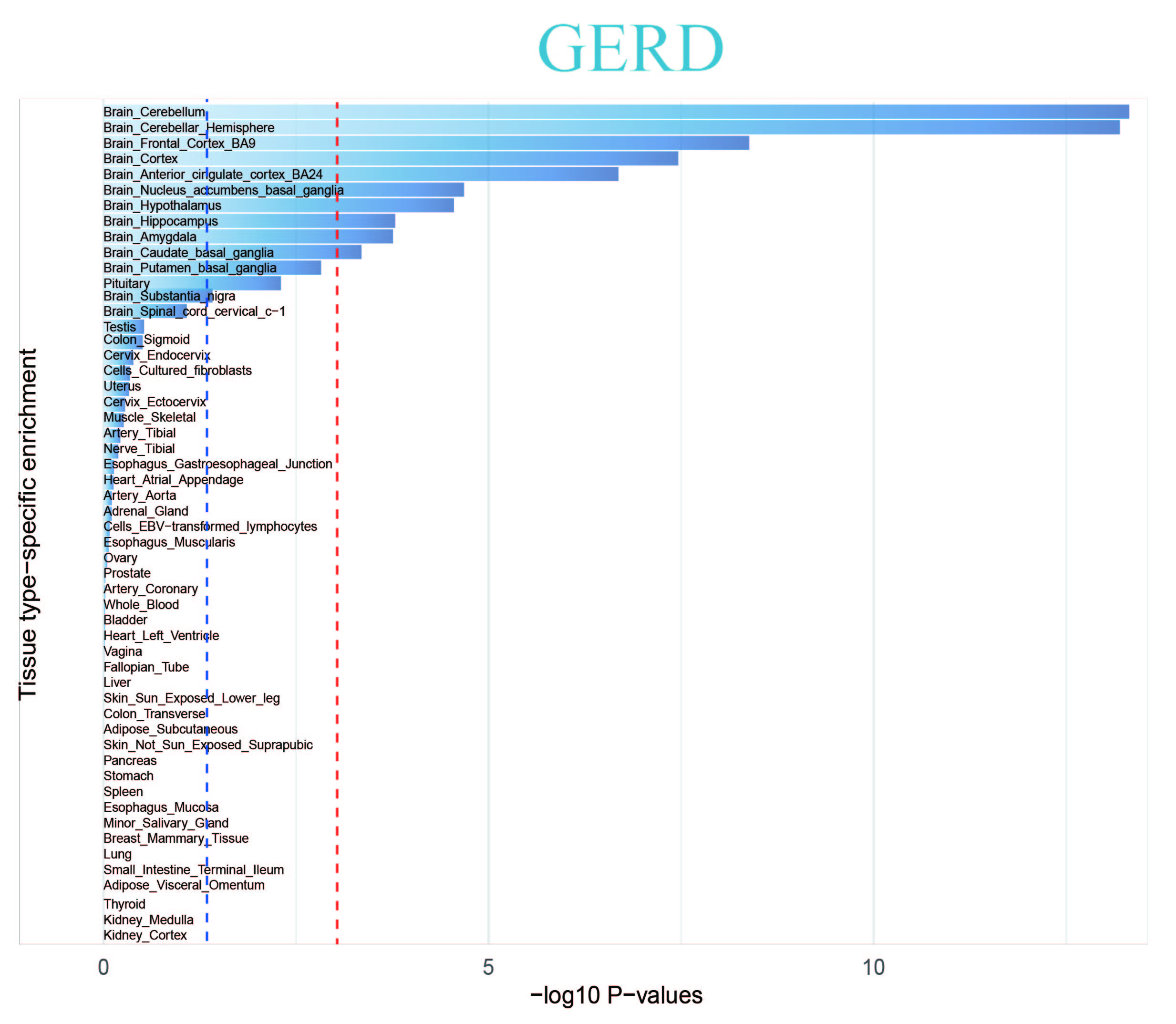


The blue dashed line represents the statistical significance threshold (0.05), while the red dashed line represents the Bonferroni-corrected significance threshold (0.05/53=9.43E-04).

# Table S9: Statistical differences due to potential sample overlap rates

| **Using specified value of concentration parameter** | | | **Using conservative value of concentration parameter** | | |
| --- | --- | --- | --- | --- | --- |
| **Overlap proportion** | **Bias** | **Type 1 error rate** | **Overlap proportion** | **Bias** | **Type 1 error rate** |
| **SI**  Concentration parameter (expected value of F statistic) = 13.50  Conservative value of concentration parameter (lower limit of one-sided 95% confidence interval) = 13.51 | | | | | |
| 0 | 0 | 0.05 | 0 | 0 | 0.05 |
| 0.1 | 0.004 | 0.05 | 0.1 | 0.004 | 0.05 |
| 0.2 | 0.007 | 0.05 | 0.2 | 0.007 | 0.05 |
| 0.3 | 0.011 | 0.05 | 0.3 | 0.011 | 0.05 |
| 0.4 | 0.015 | 0.05 | 0.4 | 0.015 | 0.05 |
| 0.5 | 0.019 | 0.05 | 0.5 | 0.019 | 0.05 |
| 0.6 | 0.022 | 0.06 | 0.6 | 0.022 | 0.06 |
| 0.7 | 0.026 | 0.06 | 0.7 | 0.026 | 0.06 |
| 0.8 | 0.03 | 0.06 | 0.8 | 0.03 | 0.06 |
| 0.9 | 0.033 | 0.07 | 0.9 | 0.033 | 0.07 |
| 1 | 0.037 | 0.07 | 1 | 0.037 | 0.07 |
| **NS**  Concentration parameter (expected value of F statistic) = 6.60  Conservative value of concentration parameter (lower limit of one-sided 95% confidence interval) = 9.73 | | | | | |
| 0 | 0 | 0.05 | 0 | 0 | 0.05 |
| 0.1 | 0.005 | 0.05 | 0.1 | 0.008 | 0.05 |
| 0.2 | 0.01 | 0.05 | 0.2 | 0.015 | 0.05 |
| 0.3 | 0.015 | 0.05 | 0.3 | 0.023 | 0.06 |
| 0.4 | 0.021 | 0.06 | 0.4 | 0.03 | 0.06 |
| 0.5 | 0.026 | 0.06 | 0.5 | 0.038 | 0.07 |
| 0.6 | 0.031 | 0.06 | 0.6 | 0.045 | 0.08 |
| 0.7 | 0.036 | 0.07 | 0.7 | 0.053 | 0.09 |
| 0.8 | 0.041 | 0.07 | 0.8 | 0.061 | 0.1 |
| 0.9 | 0.046 | 0.08 | 0.9 | 0.068 | 0.12 |
| 1 | 0.051 | 0.09 | 1 | 0.076 | 0.13 |
| **ES**  Concentration parameter (expected value of F statistic) = 7.11  Conservative value of concentration parameter (lower limit of one-sided 95% confidence interval) = 9.61 | | | | | |
| 0 | 0 | 0.05 | 0 | 0 | 0.05 |
| 0.1 | 0.005 | 0.05 | 0.1 | 0.007 | 0.05 |
| 0.2 | 0.01 | 0.05 | 0.2 | 0.014 | 0.05 |
| 0.3 | 0.016 | 0.05 | 0.3 | 0.021 | 0.06 |
| 0.4 | 0.021 | 0.06 | 0.4 | 0.028 | 0.06 |
| 0.5 | 0.026 | 0.06 | 0.5 | 0.035 | 0.07 |
| 0.6 | 0.031 | 0.06 | 0.6 | 0.042 | 0.07 |
| 0.7 | 0.036 | 0.07 | 0.7 | 0.049 | 0.08 |
| 0.8 | 0.042 | 0.07 | 0.8 | 0.056 | 0.09 |
| 0.9 | 0.047 | 0.08 | 0.9 | 0.063 | 0.11 |
| 1 | 0.052 | 0.09 | 1 | 0.07 | 0.12 |
| **CPD**  Concentration parameter (expected value of F statistic) =28.65  Conservative value of concentration parameter (lower limit of one-sided 95% confidence interval) = 29.55 | | | | | |
| 0 | 0 | 0.05 | 0 | 0 | 0.05 |
| 0.1 | 0.001 | 0.05 | 0.1 | 0.001 | 0.05 |
| 0.2 | 0.001 | 0.05 | 0.2 | 0.001 | 0.05 |
| 0.3 | 0.002 | 0.05 | 0.3 | 0.002 | 0.05 |
| 0.4 | 0.003 | 0.05 | 0.4 | 0.003 | 0.05 |
| 0.5 | 0.003 | 0.05 | 0.5 | 0.003 | 0.05 |
| 0.6 | 0.004 | 0.05 | 0.6 | 0.004 | 0.05 |
| 0.7 | 0.005 | 0.05 | 0.7 | 0.005 | 0.05 |
| 0.8 | 0.005 | 0.05 | 0.8 | 0.005 | 0.05 |
| 0.9 | 0.006 | 0.05 | 0.9 | 0.006 | 0.05 |
| 1 | 0.006 | 0.05 | 1 | 0.006 | 0.05 |
| **ASI**  Concentration parameter (expected value of F statistic) = 22.60  Conservative value of concentration parameter (lower limit of one-sided 95% confidence interval) = 28.35 | | | | | |
| 0 | 0 | 0.05 | 0 | 0 | 0.05 |
| 0.1 | 0 | 0.05 | 0.1 | 0 | 0.05 |
| 0.2 | 0.001 | 0.05 | 0.2 | 0.001 | 0.05 |
| 0.3 | 0.001 | 0.05 | 0.3 | 0.001 | 0.05 |
| 0.4 | 0.001 | 0.05 | 0.4 | 0.001 | 0.05 |
| 0.5 | 0.002 | 0.05 | 0.5 | 0.002 | 0.05 |
| 0.6 | 0.002 | 0.05 | 0.6 | 0.002 | 0.05 |
| 0.7 | 0.002 | 0.05 | 0.7 | 0.002 | 0.05 |
| 0.8 | 0.003 | 0.05 | 0.8 | 0.003 | 0.05 |
| 0.9 | 0.003 | 0.05 | 0.9 | 0.003 | 0.05 |
| 1 | 0.003 | 0.05 | 1 | 0.003 | 0.05 |

Data comes from https://sb452.shinyapps.io/overlap/, showing the possibility of type 1 errors that may occur in different sample overlaps, as well as the expected F-statistic value.

# Table S10: Genetic instrumental variables for five smoking behavior used in Mendelian randomization analysis

| SNP | Chromosome | Position | ALT/REF | Beta | Se | Eaf | P-value | F-statistics |
| --- | --- | --- | --- | --- | --- | --- | --- | --- |
| **SI** |  |  |  |  |  |  |  |  |
| rs10001365 | 4 | 147797214 | G/A | 0.02 | 3.64E-03 | 0.60 | 6.65E-12 | 47.10 |
| rs10114490 | 9 | 11070165 | G/A | 0.03 | 4.53E-03 | 0.80 | 1.81E-08 | 31.70 |
| rs10233018 | 7 | 117545837 | G/A | 0.03 | 3.56E-03 | 0.50 | 2.75E-14 | 57.90 |
| rs10279261 | 7 | 133589846 | G/A | 0.02 | 3.66E-03 | 0.38 | 5.00E-09 | 34.20 |
| rs10498846 | 6 | 67475484 | T/C | 0.02 | 3.56E-03 | 0.47 | 6.62E-09 | 33.60 |
| rs1050847 | 16 | 87443734 | C/T | 0.02 | 3.59E-03 | 0.50 | 1.67E-09 | 36.30 |
| rs10905461 | 10 | 8803551 | T/C | 0.02 | 4.15E-03 | 0.28 | 7.35E-09 | 33.40 |
| rs11057005 | 12 | 16748721 | A/G | 0.02 | 3.58E-03 | 0.57 | 4.85E-09 | 34.20 |
| rs11658881 | 17 | 2072949 | G/A | 0.02 | 3.61E-03 | 0.42 | 2.43E-08 | 31.10 |
| rs11712680 | 3 | 75009019 | A/C | 0.03 | 4.58E-03 | 0.83 | 3.51E-09 | 34.90 |
| rs12025237 | 1 | 154205120 | A/C | 0.03 | 5.34E-03 | 0.88 | 6.52E-10 | 38.20 |
| rs12186738 | 5 | 103822511 | G/T | 0.03 | 5.02E-03 | 0.85 | 3.42E-11 | 43.90 |
| rs12333760 | 7 | 99185747 | T/C | 0.03 | 4.80E-03 | 0.80 | 1.44E-09 | 36.60 |
| rs12474587 | 2 | 162802993 | T/G | 0.03 | 3.58E-03 | 0.40 | 1.25E-14 | 59.50 |
| rs13261666 | 8 | 59814666 | G/T | 0.03 | 3.56E-03 | 0.48 | 3.90E-14 | 57.20 |
| rs134529 | 22 | 28781758 | T/C | 0.02 | 3.66E-03 | 0.65 | 4.85E-08 | 29.80 |
| rs1385108 | 5 | 154843438 | T/C | 0.02 | 4.16E-03 | 0.24 | 3.00E-09 | 35.20 |
| rs1869243 | 3 | 5724536 | C/T | 0.02 | 3.56E-03 | 0.48 | 2.97E-08 | 30.70 |
| rs1899896 | 8 | 93201036 | T/C | 0.03 | 3.89E-03 | 0.29 | 1.04E-11 | 46.30 |
| rs2046850 | 1 | 210304319 | C/T | 0.02 | 4.48E-03 | 0.81 | 3.03E-08 | 30.70 |
| rs2140114 | 7 | 3407568 | C/T | 0.02 | 3.73E-03 | 0.48 | 4.70E-10 | 38.80 |
| rs2378662 | 9 | 86707289 | A/G | 0.02 | 3.57E-03 | 0.56 | 4.16E-09 | 34.50 |
| rs2631024 | 8 | 92021036 | A/G | 0.02 | 4.03E-03 | 0.26 | 1.18E-08 | 32.50 |
| rs266047 | 2 | 104132805 | G/A | 0.03 | 3.74E-03 | 0.47 | 3.36E-16 | 66.60 |
| rs3001723 | 1 | 44037685 | A/G | 0.03 | 3.90E-03 | 0.32 | 8.12E-18 | 73.90 |
| rs3800227 | 6 | 108994161 | G/A | 0.02 | 4.06E-03 | 0.70 | 1.93E-08 | 31.60 |
| rs3904512 | 13 | 38357471 | G/A | 0.02 | 3.58E-03 | 0.57 | 3.23E-09 | 35.00 |
| rs4044321 | 5 | 166989513 | A/G | 0.03 | 3.71E-03 | 0.36 | 6.08E-14 | 56.30 |
| rs4543592 | 9 | 3014254 | C/T | 0.02 | 3.56E-03 | 0.47 | 7.46E-10 | 37.90 |
| rs4674993 | 2 | 226332033 | A/G | 0.03 | 4.44E-03 | 0.79 | 1.32E-08 | 32.30 |
| rs4781977 | 16 | 17572674 | T/C | 0.02 | 4.36E-03 | 0.80 | 4.54E-08 | 29.90 |
| rs6265 | 11 | 27679916 | C/T | 0.03 | 4.58E-03 | 0.80 | 3.77E-12 | 48.20 |
| rs6433897 | 2 | 182034448 | C/T | 0.02 | 4.06E-03 | 0.75 | 3.16E-08 | 30.60 |
| rs66680800 | 3 | 86037162 | G/T | 0.02 | 3.65E-03 | 0.60 | 2.83E-08 | 30.80 |
| rs6669839 | 1 | 50625979 | T/C | 0.03 | 4.40E-03 | 0.20 | 3.36E-09 | 35.00 |
| rs6893752 | 5 | 60374912 | A/G | 0.02 | 4.07E-03 | 0.23 | 3.25E-09 | 35.00 |
| rs7197072 | 16 | 746884 | C/T | 0.02 | 4.17E-03 | 0.76 | 2.77E-09 | 35.30 |
| rs7224742 | 17 | 30742310 | C/T | 0.02 | 3.66E-03 | 0.41 | 1.43E-08 | 32.10 |
| rs72789632 | 5 | 106836310 | C/T | 0.03 | 5.29E-03 | 0.88 | 5.02E-10 | 38.70 |
| rs7555507 | 1 | 73860631 | C/T | 0.02 | 3.56E-03 | 0.50 | 1.14E-11 | 46.10 |
| rs7929518 | 11 | 85980958 | G/A | 0.02 | 4.28E-03 | 0.77 | 1.56E-08 | 32.00 |
| rs7938812 | 11 | 112911004 | G/T | 0.04 | 3.64E-03 | 0.42 | 2.71E-33 | 145.00 |
| rs7969559 | 12 | 69655167 | A/G | 0.02 | 3.96E-03 | 0.31 | 7.31E-10 | 37.90 |
| rs962625 | 4 | 28473524 | G/A | 0.02 | 4.04E-03 | 0.24 | 4.37E-09 | 34.50 |
| rs993700 | 4 | 67825894 | T/C | 0.03 | 4.29E-03 | 0.23 | 1.53E-09 | 36.50 |
| **NS** |  |  |  |  |  |  |  |  |
| rs10774625 | 12 | 112007756 | A/G | 0.00659074 | 1.00E-03 | 0.50 | 5.00E-11 | 43.16 |
| rs10956808 | 8 | 92777433 | T/G | 0.00671424 | 1.02E-03 | 0.58 | 4.70E-11 | 43.31 |
| rs10988799 | 9 | 102158106 | T/C | 0.00594728 | 1.01E-03 | 0.53 | 3.60E-09 | 34.82 |
| rs11165623 | 1 | 96893000 | A/G | 0.00549631 | 1.00E-03 | 0.50 | 4.40E-08 | 29.96 |
| rs1124639 | 2 | 200775744 | C/T | 0.00659743 | 1.01E-03 | 0.55 | 6.70E-11 | 42.61 |
| rs1150023 | 10 | 9360823 | A/C | 0.00645478 | 1.14E-03 | 0.26 | 1.60E-08 | 31.88 |
| rs1174864 | 7 | 53127559 | A/G | 0.00620597 | 1.01E-03 | 0.55 | 8.40E-10 | 37.66 |
| rs12209519 | 6 | 67549140 | G/A | 0.00569138 | 1.03E-03 | 0.41 | 2.90E-08 | 30.76 |
| rs12333760 | 7 | 99185747 | T/C | 0.00828091 | 1.36E-03 | 0.83 | 9.90E-10 | 37.34 |
| rs1324481 | 1 | 33909508 | G/T | 0.00689667 | 1.08E-03 | 0.68 | 1.60E-10 | 40.87 |
| rs1373178 | 18 | 49967811 | T/G | 0.00624613 | 1.03E-03 | 0.41 | 1.20E-09 | 36.96 |
| rs1549212 | 5 | 166996722 | C/T | 0.00662977 | 1.04E-03 | 0.37 | 1.80E-10 | 40.71 |
| rs17584022 | 11 | 59190650 | G/A | 0.00659301 | 1.11E-03 | 0.71 | 2.70E-09 | 35.36 |
| rs1876066 | 10 | 10043822 | T/C | 0.00569426 | 1.01E-03 | 0.50 | 1.90E-08 | 31.57 |
| rs1899896 | 8 | 93201036 | T/C | 0.00723263 | 1.10E-03 | 0.30 | 5.50E-11 | 43.00 |
| rs2155292 | 11 | 112912303 | G/A | 0.0147459 | 1.03E-03 | 0.39 | 2.00E-46 | 204.69 |
| rs2175207 | 10 | 87357738 | G/A | 0.00760405 | 1.37E-03 | 0.16 | 2.90E-08 | 30.75 |
| rs28809490 | 22 | 46422938 | G/A | 0.00633021 | 1.09E-03 | 0.69 | 5.90E-09 | 33.86 |
| rs35498642 | 3 | 85997368 | C/T | 0.00624463 | 1.03E-03 | 0.61 | 1.30E-09 | 36.87 |
| rs3783177 | 13 | 101151360 | T/G | 0.00728636 | 1.18E-03 | 0.76 | 7.30E-10 | 37.93 |
| rs3790286 | 20 | 19655938 | C/T | 0.00559103 | 1.01E-03 | 0.54 | 3.40E-08 | 30.47 |
| rs41513151 | 7 | 121976078 | A/G | 0.00725566 | 1.22E-03 | 0.22 | 2.80E-09 | 35.31 |
| rs465646 | 6 | 111620758 | G/A | 0.0117393 | 1.37E-03 | 0.16 | 1.30E-17 | 72.97 |
| rs528301 | 2 | 45154908 | A/G | 0.00759775 | 1.01E-03 | 0.55 | 5.10E-14 | 56.71 |
| rs61785503 | 1 | 50528591 | T/C | 0.00704819 | 1.18E-03 | 0.24 | 2.50E-09 | 35.52 |
| rs7024687 | 9 | 3176796 | A/G | 0.00582717 | 1.02E-03 | 0.48 | 1.00E-08 | 32.84 |
| rs7162423 | 15 | 74052756 | C/T | 0.00561656 | 1.01E-03 | 0.56 | 2.70E-08 | 30.89 |
| rs73058737 | 3 | 34536095 | T/G | 0.00615596 | 1.11E-03 | 0.29 | 3.30E-08 | 30.55 |
| rs7572027 | 2 | 226286009 | T/C | 0.00709608 | 1.29E-03 | 0.81 | 3.40E-08 | 30.48 |
| rs75919030 | 17 | 50200035 | T/C | 0.0067996 | 1.15E-03 | 0.74 | 2.90E-09 | 35.23 |
| rs763053 | 16 | 735921 | T/C | 0.00842129 | 1.21E-03 | 0.77 | 2.90E-12 | 48.73 |
| rs7870475 | 9 | 128134034 | C/T | 0.00565884 | 1.01E-03 | 0.48 | 1.80E-08 | 31.69 |
| rs7901348 | 10 | 63679281 | T/G | 0.00602763 | 1.02E-03 | 0.45 | 3.10E-09 | 35.13 |
| rs7969559 | 12 | 69655167 | A/G | 0.0062217 | 1.12E-03 | 0.28 | 2.60E-08 | 30.97 |
| rs899632 | 4 | 57793751 | T/C | 0.00702938 | 1.03E-03 | 0.61 | 9.90E-12 | 46.34 |
| rs904592 | 3 | 25193102 | T/C | 0.00569212 | 1.01E-03 | 0.48 | 1.90E-08 | 31.56 |
| rs905871 | 11 | 4661285 | A/G | 0.00583757 | 1.06E-03 | 0.66 | 3.70E-08 | 30.28 |
| **ES** |  |  |  |  |  |  |  |  |
| rs10179482 | 2 | 81004626 | A/G | 0.01 | 1.01E-03 | 0.49 | 9.90E-09 | 32.86 |
| rs10212155 | 3 | 117785505 | A/G | 0.01 | 1.42E-03 | 0.85 | 3.40E-13 | 52.97 |
| rs10233018 | 7 | 117545837 | G/A | 0.01 | 1.00E-03 | 0.50 | 9.20E-15 | 60.07 |
| rs1040070 | 1 | 74977870 | C/G | -0.01 | 1.02E-03 | 0.57 | 6.20E-09 | 33.78 |
| rs10774625 | 12 | 112007756 | G/A | -0.01 | 1.00E-03 | 0.50 | 5.00E-11 | 43.16 |
| rs10952199 | 7 | 1688369 | T/C | -0.01 | 1.02E-03 | 0.43 | 1.40E-11 | 45.68 |
| rs10956808 | 8 | 92777433 | G/T | -0.01 | 1.02E-03 | 0.42 | 4.70E-11 | 43.31 |
| rs10988799 | 9 | 102158106 | T/C | 0.01 | 1.01E-03 | 0.53 | 3.60E-09 | 34.82 |
| rs11165623 | 1 | 96893000 | A/G | 0.01 | 1.00E-03 | 0.50 | 4.40E-08 | 29.96 |
| rs1124639 | 2 | 200775744 | C/T | 0.01 | 1.01E-03 | 0.55 | 6.70E-11 | 42.61 |
| rs1150023 | 10 | 9360823 | C/A | -0.01 | 1.14E-03 | 0.74 | 1.60E-08 | 31.88 |
| rs1174864 | 7 | 53127559 | A/G | 0.01 | 1.01E-03 | 0.55 | 8.40E-10 | 37.66 |
| rs12209519 | 6 | 67549140 | G/A | 0.01 | 1.03E-03 | 0.41 | 2.90E-08 | 30.76 |
| rs12244388 | 10 | 104760752 | A/G | 0.01 | 1.06E-03 | 0.34 | 6.80E-16 | 65.19 |
| rs12272735 | 11 | 7953053 | G/A | 0.01 | 1.03E-03 | 0.39 | 1.80E-08 | 31.67 |
| rs12333760 | 7 | 99185747 | C/T | -0.01 | 1.36E-03 | 0.17 | 9.90E-10 | 37.34 |
| rs13162305 | 5 | 12122698 | T/A | 0.01 | 1.07E-03 | 0.34 | 2.20E-08 | 31.28 |
| rs1324481 | 1 | 33909508 | G/T | 0.01 | 1.08E-03 | 0.68 | 1.60E-10 | 40.87 |
| rs13246563 | 7 | 3464896 | G/C | -0.01 | 1.01E-03 | 0.54 | 5.20E-11 | 43.11 |
| rs1373178 | 18 | 49967811 | G/T | -0.01 | 1.03E-03 | 0.59 | 1.20E-09 | 36.96 |
| rs1549212 | 5 | 166996722 | T/C | -0.01 | 1.04E-03 | 0.63 | 1.80E-10 | 40.71 |
| rs1718705 | 3 | 61823653 | C/G | 0.01 | 1.08E-03 | 0.32 | 2.50E-09 | 35.52 |
| rs17584022 | 11 | 59190650 | A/G | -0.01 | 1.11E-03 | 0.29 | 2.70E-09 | 35.36 |
| rs1876066 | 10 | 10043822 | C/T | -0.01 | 1.01E-03 | 0.50 | 1.90E-08 | 31.57 |
| rs1899896 | 8 | 93201036 | T/C | 0.01 | 1.10E-03 | 0.30 | 5.50E-11 | 43.00 |
| rs2155292 | 11 | 112912303 | G/A | 0.01 | 1.03E-03 | 0.39 | 2.00E-46 | 204.69 |
| rs2175207 | 10 | 87357738 | G/A | 0.01 | 1.37E-03 | 0.16 | 2.90E-08 | 30.75 |
| rs2183573 | 21 | 40574305 | G/A | -0.01 | 1.02E-03 | 0.57 | 9.20E-09 | 33.00 |
| rs28809490 | 22 | 46422938 | A/G | -0.01 | 1.09E-03 | 0.31 | 5.90E-09 | 33.86 |
| rs34335016 | 1 | 154205120 | T/A | -0.01 | 1.54E-03 | 0.12 | 2.20E-08 | 31.32 |
| rs35498642 | 3 | 85997368 | T/C | -0.01 | 1.03E-03 | 0.39 | 1.30E-09 | 36.87 |
| rs3783177 | 13 | 101151360 | G/T | -0.01 | 1.18E-03 | 0.24 | 7.30E-10 | 37.93 |
| rs3790286 | 20 | 19655938 | C/T | 0.01 | 1.01E-03 | 0.54 | 3.40E-08 | 30.47 |
| rs41513151 | 7 | 121976078 | A/G | 0.01 | 1.22E-03 | 0.22 | 2.80E-09 | 35.31 |
| rs4422110 | 2 | 146114898 | T/C | -0.01 | 1.01E-03 | 0.53 | 1.50E-18 | 77.21 |
| rs465646 | 6 | 111620758 | A/G | -0.01 | 1.37E-03 | 0.84 | 1.30E-17 | 72.97 |
| rs528301 | 2 | 45154908 | A/G | 0.01 | 1.01E-03 | 0.55 | 5.10E-14 | 56.71 |
| rs61785503 | 1 | 50528591 | T/C | 0.01 | 1.18E-03 | 0.24 | 2.50E-09 | 35.52 |
| rs6265 | 11 | 27679916 | T/C | -0.01 | 1.28E-03 | 0.19 | 6.70E-11 | 42.60 |
| rs6499595 | 16 | 72934443 | C/G | -0.01 | 1.03E-03 | 0.40 | 1.80E-10 | 40.64 |
| rs67716713 | 2 | 104279490 | A/C | -0.01 | 1.00E-03 | 0.49 | 4.00E-13 | 52.67 |
| rs7024687 | 9 | 3176796 | G/A | -0.01 | 1.02E-03 | 0.52 | 1.00E-08 | 32.84 |
| rs7162423 | 15 | 74052756 | T/C | -0.01 | 1.01E-03 | 0.44 | 2.70E-08 | 30.89 |
| rs73058737 | 3 | 34536095 | T/G | 0.01 | 1.11E-03 | 0.29 | 3.30E-08 | 30.55 |
| rs7572027 | 2 | 226286009 | T/C | 0.01 | 1.29E-03 | 0.81 | 3.40E-08 | 30.48 |
| rs7585579 | 2 | 60024857 | G/C | 0.01 | 1.01E-03 | 0.49 | 1.80E-10 | 40.73 |
| rs75919030 | 17 | 50200035 | C/T | -0.01 | 1.15E-03 | 0.26 | 2.90E-09 | 35.23 |
| rs763053 | 16 | 735921 | C/T | -0.01 | 1.21E-03 | 0.23 | 2.90E-12 | 48.73 |
| rs7758291 | 6 | 129346046 | C/A | 0.01 | 1.08E-03 | 0.68 | 2.40E-08 | 31.11 |
| rs7870475 | 9 | 128134034 | C/T | 0.01 | 1.01E-03 | 0.48 | 1.80E-08 | 31.69 |
| rs7901348 | 10 | 63679281 | G/T | -0.01 | 1.02E-03 | 0.55 | 3.10E-09 | 35.13 |
| rs7969559 | 12 | 69655167 | G/A | -0.01 | 1.12E-03 | 0.72 | 2.60E-08 | 30.97 |
| rs899632 | 4 | 57793751 | C/T | -0.01 | 1.03E-03 | 0.39 | 9.90E-12 | 46.34 |
| rs904592 | 3 | 25193102 | T/C | 0.01 | 1.01E-03 | 0.48 | 1.90E-08 | 31.56 |
| rs905871 | 11 | 4661285 | G/A | -0.01 | 1.06E-03 | 0.34 | 3.70E-08 | 30.28 |
| rs9597810 | 13 | 59283765 | G/C | -0.01 | 1.07E-03 | 0.33 | 4.30E-08 | 30.01 |
| **CPD** |  |  |  |  |  |  |  |  |
| rs1579233 | 16 | 52082467 | A/G | 0.03 | 5.56E-03 | 0.43 | 1.07E-08 | 32.70 |
| rs2424888 | 20 | 31062176 | A/G | 0.03 | 5.64E-03 | 0.41 | 2.76E-09 | 35.30 |
| rs58379124 | 8 | 42579739 | C/T | 0.07 | 6.50E-03 | 0.75 | 9.00E-25 | 106.00 |
| rs632811 | 15 | 59170964 | A/G | 0.04 | 6.41E-03 | 0.65 | 1.03E-08 | 32.80 |
| rs7431710 | 3 | 48935583 | G/A | 0.03 | 5.81E-03 | 0.36 | 1.82E-09 | 36.20 |
| rs75494138 | 11 | 46520302 | T/C | 0.06 | 1.06E-02 | 0.06 | 1.45E-08 | 32.10 |
| rs790564 | 8 | 64604218 | A/C | 0.04 | 6.19E-03 | 0.28 | 3.97E-11 | 43.60 |
| rs7951365 | 11 | 16377119 | C/T | 0.04 | 5.97E-03 | 0.31 | 6.63E-11 | 42.60 |
| **ASI** |  |  |  |  |  |  |  |  |
| rs10200107 | 2 | 63622470 | G/A | 0.02 | 2.78E-03 | 0.44 | 1.84E-12 | 49.60 |
| rs11780471 | 8 | 27344719 | A/G | 0.04 | 5.81E-03 | 0.06 | 7.00E-11 | 42.50 |
| rs319748 | 17 | 31554533 | G/A | 0.02 | 3.07E-03 | 0.29 | 3.08E-08 | 30.70 |
| rs624833 | 4 | 2881256 | G/T | 0.02 | 3.01E-03 | 0.31 | 8.61E-09 | 33.10 |

ALT/REF: Effect allele/ Other allele; SI: Smoking initiation; NS: Never smoking; ES: Ever smoking; CPD: Cigarettes smoked per day; ASI: Age of smoking initiation.

# Figure S8: Sensitivity analysis of Smoking initiation and GERD



Scatter plot (top left) and funnel plot (top right)

Leave-one-out analysis (bottom left) and forest plot (bottom right)

The forest plot displays the individual Wald ratios for each SNP, with mean values not exceeding the 0 scale line.

"Leave-One-Out" Analysis of SI and GERD. Leave-one-out analysis suggests that no SNP had a significant impact on the outcome (all bars are on the right side of 0)."

#

Figure S9: Sensitivity analysis of Never smoking and GERD

Scatter plot (top left) and funnel plot (top right)

Leave-one-out analysis (bottom left) and forest plot (bottom right)

The forest plot displays the individual Wald ratios for each SNP, with mean values not exceeding the 0 scale line.

"Leave-One-Out" Analysis of NS and GERD. Leave-one-out analysis suggests that no SNP had a significant impact on the outcome (all bars are on the right side of 0)."

# Figure S10: Sensitivity analysis of Ever Smoking and GERD



Scatter plot (top left) and funnel plot (top right)

Leave-one-out analysis (bottom left) and forest plot (bottom right)

The forest plot displays the individual Wald ratios for each SNP, with mean values not exceeding the 0 scale line.

"Leave-One-Out" Analysis of ES and GERD. Leave-one-out analysis suggests that no SNP had a significant impact on the outcome (all bars are on the right side of 0)."

# Figure S11: Sensitivity analysis of Age of smoking initiation and GERD



Scatter plot (top left) and funnel plot (top right)

Leave-one-out analysis (bottom left) and forest plot (bottom right)

The forest plot displays the individual Wald ratios for each SNP, with mean values not exceeding the 0 scale line.

"Leave-One-Out" Analysis of ASI and GERD. Leave-one-out analysis suggests that no SNP had a significant impact on the outcome (all bars are on the right side of 0)."

#

Figure S12: Sensitivity analysis of Cigarettes smoked per day and GERD

Scatter plot (top left) and funnel plot (top right)

Leave-one-out analysis (bottom left) and forest plot (bottom right)

The forest plot displays the individual Wald ratios for each SNP, with mean values not exceeding the 0 scale line.

"Leave-One-Out" Analysis of CPD and GERD. Leave-one-out analysis suggests that no SNP had a significant impact on the outcome (all bars are on the right side of 0)."

# Table S11: Multivariate Mendelian randomization of Smoking behavior and GERD after adjustment for alcohol

| **Exposure** | **Outcome** | **SNP** | **OR (95% CI)** | **P** |
| --- | --- | --- | --- | --- |
| SI  Alcoholic drinks per week | Gastroesophageal reflux disease (GERD) | 52  17 | 1.60(1.48,1.72)  1.15(1.06,1.24) | 8.40E-15  1.27E-02 |
| NS  Alcoholic drinks per week |  | 54  18 | 0.27(0.13,0.41)  1.23(0.71,1.35) | 4.72E-10  0.12 |
| ES  Alcoholic drinks per week |  | 58  15 | 2.91(2.42,3.40)  1.23(0.71,1.35) | 1.81E-05  0.97 |
| CPD  Alcoholic drinks per week |  | 12  20 | 1.15(1.04,1.27)  1.17(0.47,2.25) | 0.02  0.99 |
| ASI  Alcoholic drinks per week |  | 4  20 | 0.45(0.22,0.68)  1.63(0.51,3.15) | 7.35E-03  0.62 |

Alcoholic drinks per week (DPW) (n = 335,394) was sourced from GWAS and the Alcohol and Nicotine use Sequencing Consortium (GSCAN) for a comprehensive whole-genome association study (GWAS). To create genetic instrumental variables (IVs) for drinking behavior, we identified variants with genome-wide significance for the three lipid traits (P < 5×10^−8^), with a linkage disequilibrium threshold (LD r^2^≤0.001) and a minimum physical distance of 10 Mb in the GWAS summary statistics.

# Figure S13: Mendelian Randomization Analysis between Smoking Behavior and GERD (FinnGen)


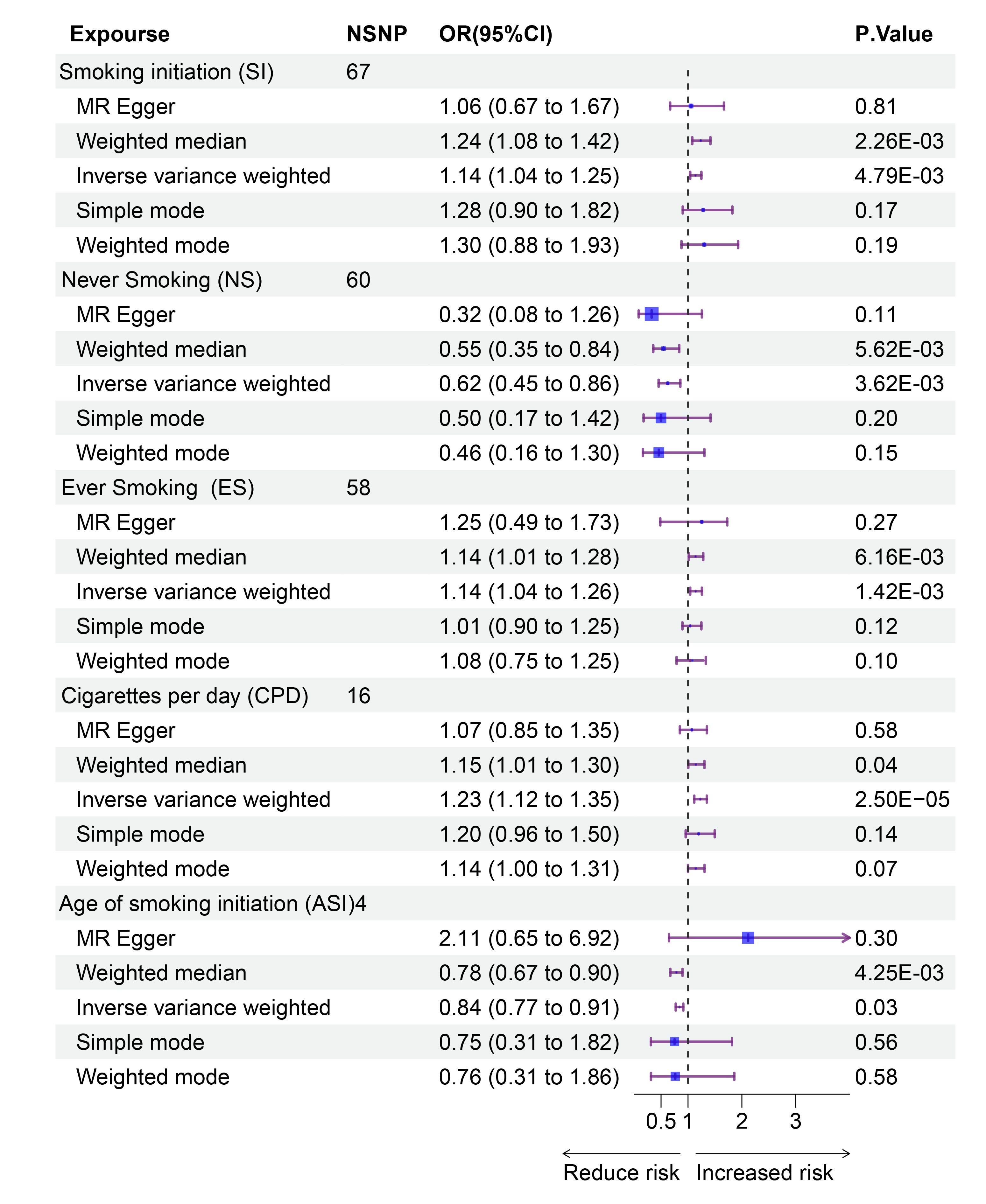


Using "inverse variance weighting (IVW)" as the primary analysis method, with MR-eggr regression, weighted median (WM) and weighted model analysis as supplements. MR: Mendelian randomization; SI: Smoking initiation; NS: Never Smoking; ES: Ever Smoking; CPD: Cigarettes smoked per day; ASI: Age of smoking initiation; GERD: Gastroesophageal reflux disease.

# Table S12: Sensitivity analysis of Mendelian randomization (FinnGen)

| **Exposure** | **Outcome** | **Cochran Q value** | **Q-pval** | **MR-Egger Intercept** | **P*-*value** | **MR-PRESSO**  **P-value** |
| --- | --- | --- | --- | --- | --- | --- |
| SI | GERD  (FinnGen) | 66.32 | 0.49 | 2.01E-03 | 0.74 | 0.77 |
| NS |  | 69.13 | 0.17 | 5.77E-03 | 0.33 | 0.04 |
| ES |  | 51.09 | 0.70 | 2.07E-03 | 0.71 | 0.69 |
| CPD |  | 28.03 | 0.06 | 1.46E-03 | 0.08 | 0.07 |
| ASI |  | 4.88 | 0.30 | -2.22E-02 | 0.20 | NA |

SI: Smoking initiation; NS: Never Smoking; ES: Ever Smoking; CPD: Cigarettes smoked per day; ASI: Age of smoking initiation; GERD: Gastroesophageal reflux disease; MR-PRESSO: Mendelian Randomization Pleiotropy RESidual Sum and Outlier.
